# Socioeconomic inequalities in cervical precancer screening among women in Ethiopia, Malawi, Rwanda, Tanzania, Zambia and Zimbabwe: analysis of Population-Based HIV Impact Assessment surveys

**DOI:** 10.1136/bmjopen-2022-067948

**Published:** 2023-06-20

**Authors:** David Chipanta, Sharon Kapambwe, Alinane Linda Nyondo-Mipando, Margaret Pascoe, Silas Amo-Agyei, Julia Bohlius, Janne Estill, Olivia Keiser

**Affiliations:** 1 ERA, UNAIDS, Geneve, Switzerland; 2 Faculty of Medicine, University of Geneva, Geneve, Switzerland; 3 World Health Organization, Harare, Zimbabwe; 4 Kamuzu University of Health Sciences, Blantyre, Malawi; 5 Clinic, Newlands Clinic, Harare, Zimbabwe; 6 Department of Economics, University of Lausanne, Lausanne, Switzerland; 7 Swiss Tropical and Public Health Institute, University of Bern, Basel, Switzerland

**Keywords:** Cancer pain, HIV & AIDS, PUBLIC HEALTH, Health policy, Health economics, Reproductive medicine

## Abstract

**Objectives:**

We examined age, residence, education and wealth inequalities and their combinations on cervical precancer screening probabilities for women. We hypothesised that inequalities in screening favoured women who were older, lived in urban areas, were more educated and wealthier.

**Design:**

Cross-sectional study using Population-Based HIV Impact Assessment data.

**Setting:**

Ethiopia, Malawi, Rwanda, Tanzania, Zambia and Zimbabwe. Differences in screening rates were analysed using multivariable logistic regressions, controlling for age, residence, education and wealth. Inequalities in screening probability were estimated using marginal effects models.

**Participants:**

Women aged 25–49 years, reporting screening.

**Outcome measures:**

Self-reported screening rates, and their inequalities in percentage points, with differences of 20%+ defined as high inequality, 5%–20% as medium, 0%–5% as low.

**Results:**

The sample size of participants ranged from 5882 in Ethiopia to 9186 in Tanzania. The screening rates were low in the surveyed countries, ranging from 3.5% (95% CI 3.1% to 4.0%) in Rwanda to 17.1% (95% CI 15.8% to 18.5%) and 17.4% (95% CI 16.1% to 18.8%) in Zambia and Zimbabwe. Inequalities in screening rates were low based on covariates. Combining the inequalities led to significant inequalities in screening probabilities between women living in rural areas aged 25–34 years, with a primary education level, from the lowest wealth quintile, and women living in urban areas aged 35–49 years, with the highest education level, from the highest wealth quintile, ranging from 4.4% in Rwanda to 44.6% in Zimbabwe.

**Conclusions:**

Cervical precancer screening rates were inequitable and low. No country surveyed achieved one-third of the WHO’s target of screening 70% of eligible women by 2030. Combining inequalities led to high inequalities, preventing women who were younger, lived in rural areas, were uneducated, and from the lowest wealth quintile from screening. Governments should include and monitor equity in their cervical precancer screening programmes.

**STRENGTHS AND LIMITATIONS OF THIS STUDY**

- Collinearity was present, especially between the wealth and residence variables which tend to correlate with each other, consistent with how the wealth variable is constructed.
- Recall bias was present as cervical precancer screening is self-reported.
- We did not control for the type of cervical precancer screening because we did not have information on the types of screening women received.

## Introduction

Although all countries are affected by cervical cancer, its incidence and mortality are more than twice and three times as high in low-income and middle-income countries as in high-income countries.^1^ In 2020, it was the leading cause of cancer deaths in 36 countries. Most of these countries were in sub-Saharan Africa, Melanesia, South America and South-Eastern Asia.^2^ Cervical cancer occurs in the lower part of the uterus that connects to the vagina. It is caused in over 70% of cases by the human papillomavirus (HPV), the most common sexually transmitted infection (STI).^1^ Most HPV strains are harmless in people with a healthy immune system.^1^ HIV-positive women, whose immunity may be compromised,^1 2^ are six times more likely to develop cervical cancer than HIV-negative women.^1^ In 2014, invasive cervical cancer was 11 times higher among women living with HIV in South Africa than in Europe 5 years after antiretroviral therapy (ART) initiation.^3^ Cervical cancer harms girls, women and their families with significant health and development consequences, yet it is preventable and curable if detected and treated early.^1 2^ HPV vaccination and regular precancer screening stop cervical precancer. Cervical precancer screening identifies precancerous cells to treat before they become cancer.^1^ Comprehensive HPV vaccination and cervical precancer screening could prevent an estimated 5.2 million cases and 3.7 million deaths over 10 years and would cost around US$3.2 billion in 50 low-income and lower-middle-income countries.^4^ In 2020, the WHO proposed the 90–70–90 target, which, if achieved by 2030, would put the world on course to eliminate cervical cancer.^1^ The 90–70–90 target seeks to ensure that 90% of girls are vaccinated against HPV by age 15 years, 70% of eligible women are screened by age 35 years and again by 45, and 90% of women identified with cervical cancer receive treatment.^1^

Cervical cancer is a disease of inequality. Its burden reflects inequalities in society, including those related to socioeconomic status. Inequality in health and healthcare is defined as the unequal distribution of health goods, services and outcomes across income or other measures of wealth. In contrast, equity is the lack of avoidable and remedial differences in the distribution of health goods and services or outcomes due to people’s socioeconomic status in society.^5–7^ Cervical precancer screening is a critical component of the 90–70–90 target.^8^ Socioeconomic inequalities are preventing women from accessing cervical precancer screening. Studies show that residence, geography, education, wealth, age, health insurance status and the capacity of health systems influence access to cervical precancer screening. Other factors include, a history of multiple sexual partners, HIV-positive status and women’s social interactions.^9–18^ These studies showed that women who were older, resided in urban areas, and were more educated and wealthier were screened more often than women who were younger, resided in rural areas, were uneducated and poor, with a few exceptions.^9 19–22^ However, these studies did not estimate the magnitude of inequalities and their combined impact on cervical precancer screening. Such studies are needed to scale up cervical precancer screening and support the prioritisation of inequalities to eliminate cervical cancer, especially in east and southern African countries, where HIV has worsened the cervical cancer burden.^23^ This study aimed to examine age, residence, education, and wealth related inequalities and their combined impacts on cervical precancer screening in Ethiopia, Malawi, Rwanda, Tanzania, Zambia and Zimbabwe. We hypothesised that significant inequalities in cervical precancer screening rates would favour older women, women who resided in urban areas, and more educated and wealthier women.

## Methods

### Data and sample

This is cross-sectional study of a secondary data analysis of available Population-Based HIV Impact Assessment (PHIA) data for countries that included cervical precancer screening variables. The countries and the years in which the PHIA surveys were conducted include Ethiopia (2017–2018),^24^ Malawi (2015–2016),^25^ Rwanda (2018–2019),^26^ Tanzania (2016–2017),^27^ Zambia (2016)^28^ and Zimbabwe (2015–2016).^29^ The PHIA surveys collected a range of health and sociodemographic data to evaluate the impact of HIV programmes in countries supported by US President’s Emergency Plan for AIDS Relief. We used the Household, and Adult data sets. In participating households, a household questionnaire was administered to the head-of-household, who provided information on the household relating to wealth, and other sociodemographic characteristics. Then, an individual questionnaire was administered to eligible and consenting adults aged 15 years or older in the household. The individual interviews assessed a range of HIV-related variables, including cervical precancer screening and HIV testing.

We restricted our analysis to women aged 25–49 years who responded to the cervical precancer questions. This is the group WHO recommends for cervical precancer screening—ages 30–49 years for the general population and 25–49 years for women living with HIV—including all gender diverse people at risk of cervical cancer.^1^

### Patient and public involvement

No patient involved.

### Variables and outcome descriptions

We assessed inequalities related to age, residence, education and wealth in cervical precancer screening according to women’s self-reports. These dimensions of inequalities are familiar sources of disadvantage or discrimination.^30^ Our primary outcome was self-reported ever testing for cervical precancer. The main predictors were age, residence, education and wealth. Other covariates were having ever been married, ever been tested for HIV, (also binary coded) and regions. Regions, provinces or zones were coded as dummy variables and included to capture the geographical variation in cervical precancer screening. Ethiopia’s data set had an urban variable, which we coded as rural if the population size was smaller than 50 000 people and urban otherwise. Online supplemental table 1 describes the variables. We used percentages to measure the magnitude of inequality between subgroups. We denoted a difference of 20% or more between two subgroups as high inequality, less than 20% but greater than 5% as medium inequality and below 5% as low inequality, as used by the WHO.^30^

10.1136/bmjopen-2022-067948.supp1 Supplementary data

### Analysis

We assessed inequalities in self-reported cervical precancer screening in three steps. For each country, we first presented sample characteristics descriptively. Second, we determined the associations between screening for cervical precancer and each dimension of inequality, and analysed the differences using multivariable logistics regression, controlling for covariates. We corrected the p values of odds ratios (OR) for multiple hypothesis testing with sharpened q’s. In step 3, we used marginal effects models to estimate the probabilities of reporting cervical precancer screening for each inequality alone and combined with other inequalities, holding the other inequalities constant at their mean values. We applied survey weights to account for complex survey design. Using jack-knife replicate weights, 95% CIs were estimated.^31^ We excluded the observation from the analysis if the gender value was missing. We did not impute the missing data since all variables had less than 2% of missing values. We used Stata V.14 for the analyses.^32^ This study conforms to the Standards for Quality Improvement Reporting Excellence (SQUIRE) reporting guidelines.^33^

## Results

The sample size of females aged 25–49 years ranged from 5882 in Ethiopia to 9186 in Tanzania. The median age and Interquartile ranges (IQRs) were 32 years^28–39^ in Ethiopia, 33 years^29–40^ in Zambia and 34 years^29–40^ in Malawi, Rwanda and Tanzania. The rate of self-reported cervical precancer screening was lowest in Rwanda (3.5% (95% CI 3.1% to 4.0%)) and highest in Zambia (17.1% (95% CI 15.8% to 18.5%)) and Zimbabwe (17.4% (95% CI 16.1% to 18.8%)). The HIV prevalence among females aged 25–49 years old ranged from 4.7% (95% CI 4.1% to 5.4%) in Rwanda to 22.6% (95% CI 21.6% to 23.7%) in Zimbabwe, and the rate of cervical precancer screening among HIV-positive females ranged from 0.4% (95% CI 0.3% to 0.5%) in Rwanda to 5.4% (95% CI 4.7% to 6.1%) in Zambia. Females aged 25–34 years comprised half or more of the sample in surveyed countries. Eighty-six per cent or more of the respondents reported ever having been married, and 86% or more reported ever having been tested for HIV. More than half of the respondents resided in rural areas, excluding Ethiopia, where all respondents resided in urban areas, 54% of them in areas with <50 000 residents. Less than 20% of the respondents were uneducated, and nearly 30% had attained a primary school education. Only in Ethiopia, Malawi and Zambia was the proportion of respondents in the top two wealth quintiles greater than that in the bottom two quintiles (table 1).

**Table 1:** Survey weighted sample characteristics by country (PHIA 2015–2019), (percentage (95% CI) sample size)

	Ethiopia	Malawi	Rwanda	Tanzania	Zambia	Zimbabwe
Cervical precancer screened	6.1 (5.3 to 6.9), 343	11.8 (10.6 to 13.1), 926	3.5 (3.1 to 4.0), 353	7.0 (6.1 to 8.1), 586	17.1 (15.8 to 18.5), 1066	17.4 (16.1 to 18.8), 1129
HIV positive	6.1 (5.3 to 7.0), 362	18.8 (17.3 to 20.3), 1152	4.7 (4.1 to 5.4), 416	9.2 (8.4 to 10.0), 897	21.2 (19.8 to 22.6), 1263	22.6 (21.6 to 23.7), 1602
Cervical precancer screened	0.9 (0.7 to 1.45), 52	3.4 (2.8 to 4.1), 249	0.4 (0.3 to 0.5), 43§	1.5 (1.1 to 2.0), 132	5.4 (4.7 to 6.1), 318	4.5 (4.0 to 5.0), 305
Age (years): median (IQR) years	32 (28 to 39)	34 years (29 to 40)	34 years (29 to 40)	34 years (29 to 40)	33 years (29 to 40)	34 years (29 to 40)
25–34	55.9 (55.7 to 56.2), 3517	53.0 (52.9 to 53.1), 3342	52.1 (52.1 to 52.2), 4508	52.2 (52.2 to 52.3), 4812	55.3 (55.2 to 55.4), 3386	54.5 (54.4 to 54.6), 3403
35–49	44.1 (43.8 to 44.3), 2365	47.0 (46.9 to 47.1), 2720	47.9 (47.8 to 47.9), 4063	47.8 (47.7 to 47.8), 4374	44.7 (44.6 to 44.8), 3027	45.5 (45.4 to 45.6), 3381
Ever married	89.7 (88.4 to 90.9), 5228	96.6 (95.9 to 97.1), 5794	86.0 (85.0 to 87.0), 7320	92.6 (91.6 to 93.6), 8565	91.7 (90.6 to 92.7), 5901	94.0 (93.3 to 94.6), 6373
Ever tested for HIV	86.4 (84.9 to 87.7), 5018	94.8 (94.0 to 95.4), 5756	95.7 (95.2 to 96.1), 8194	89.2 (88.2 to 90.1), 8206	92.3 (91.5 to 93.1), 5878	94.8 (94.1 to 95.4), 6375
Location urban	54.0 (48.8 to 59.1), 3215	19.0 (16.3 to 21.9), 2289	20.2 (16.5 to 24.5), 2159	39.4 (35.7 to 43.4), 3299	45.9 (42.4 to 49.4), 2867	38.9 (37.0 to 40.9), 2317
Education						
No education	22.1 (20.0 to 24.3), 1307	14.5 (13.2 to 15.9), 747	11.5 (10.7 to 12.4), 953	17.8 (16.3 to 19.5), 1816	8.5 (7.2 to 9.9), 545	1.8 (1.4 to 2.3), 135
Primary	38.7 (36.7 to 40.7), 2210	66.2 (64.4 to 68.0), 3672	68.1 (66.4 to 69.7), 5666	65.8 (64.2 to 67.4), 5931	51.8 (49.5 to 54.1), 3329	27.6 (26.1 to 29.2), 2044
Secondary	21.1 (19.6 to 22.7), 1230	16.7 (15.3 to 18.3), 1351	15.8 (14.7 to 16.9), 1434	14.7 (13.5 to 15.9), 1297	30.3 (28.4 to 32.3), 1953	62.8 (61.1 to 64.4), 4146
Higher	18.1 (16.3 to 20.0), 1122	2.5 (2.1 to 3.0), 288	4.6 (3.9 to 5.5), 510	1.7 (1.3 to 2.1), 137	9.4 (8.0 to 11.1), 583	7.8 (6.5 to 9.2), 455
Wealth quintiles						
1, lowest	14.6 (12.5 to 17.0), 910	17.3 (15.7 to 19.1), 748	20.4 (18.3 to 22.7), 1600	18.3 (16.1 to 20.8), 1821	16.3 (14.6 to 18.1), 1055	18.9 (17.3 to 20.6), 1530
2	16.1 (14.5 to 17.8), 972	18.5 (17.0 to 20.1), 863	19.2 (17.5 to 21.0), 1542	19.6 (18.0 to 21.4), 1807	17.5 (16.1 to 19.0), 1140	18.4 (17.2 to 19.6), 1318
3	19.1 (17.6 to 20.6), 1144	19.7 (18.3 to 21.2), 957	19.5 (18.1 to 21.0), 1580	20.0 (18.3 to 21.8), 2063	19.7 (17.9 to 21.7), 1258	17.5 (16.0 to 19.1), 1180
4	23.1 (21.3 to 25.0), 1356	20.7 (19.3 to 22.2), 1207	20.2 (18.7 to 21.9), 1662	20.5 (18.6 to 22.5), 1798	22.2 (20 to 24.6), 1424	21.4 (19.1 to 23.8), 1282
5, highest	27.1 (24.5 to 29.9), 1500	23.7 (21.6 to 25.9), 2287	20.6 (18.1 to 23.3), 2182	21.6 (19.4 to 24.0), 1694	24.3 (21.7 to 27.1), 1506	23.9 (21.4 to 26.5), 1474
N	5882	6062	8571	9186	6413	6784

Self-reported cervical precancer screening, proportions in percentages, Pearson’s test with jack-knife 95% CIs, N is number of observations. Ethiopia dataset had an urban variable which was divided into population sizes <50 000 and >50 000. We classified a population size of <50 000 as rural and >50 000 as urban.

§ Estimate based on 25–49 observations and should be interpreted with caution.

IQR, Interquartile Range; PHIA, Population-Based HIV Impact Assessment.

**Figure 1:**
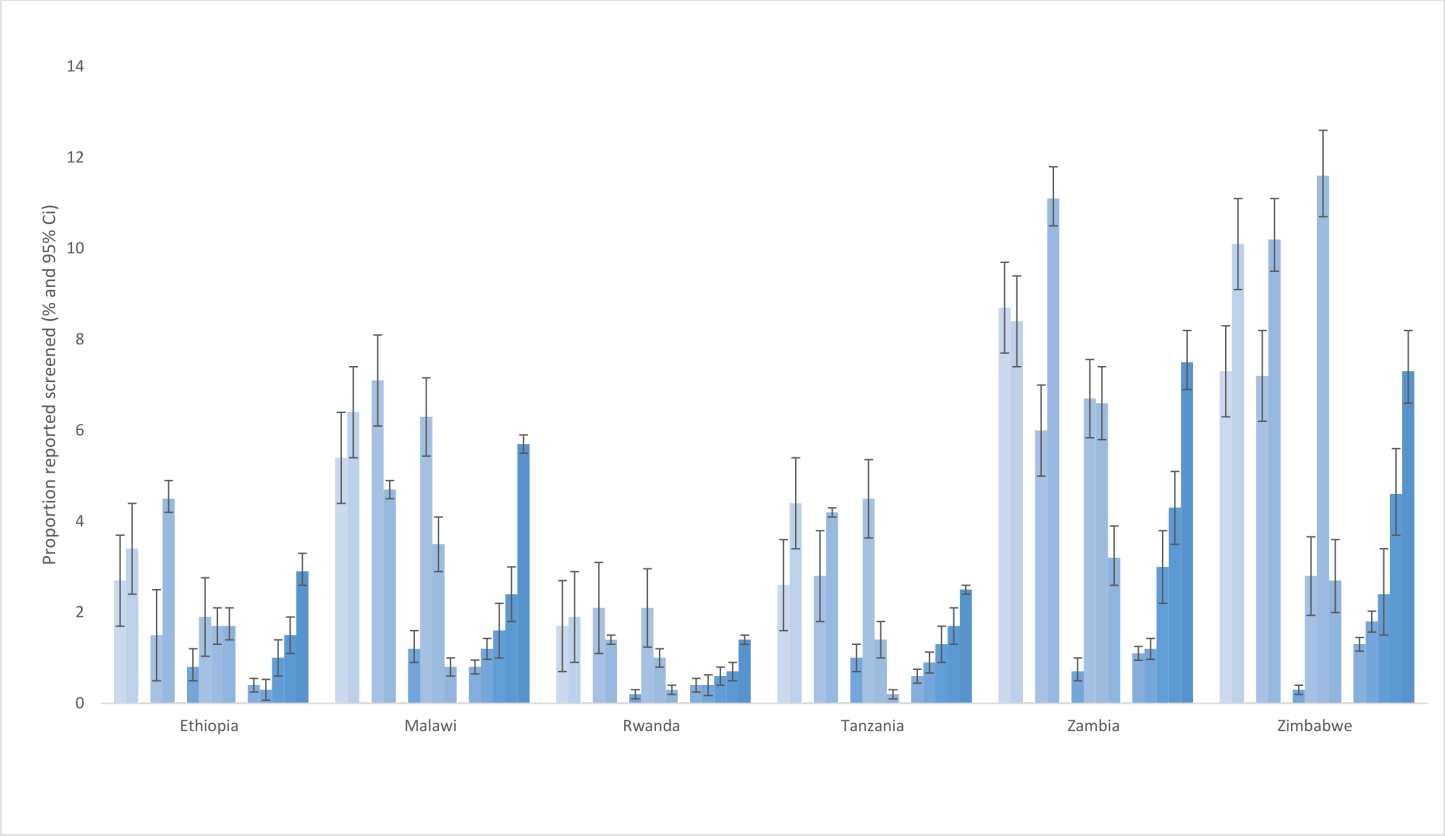
Survey weighted proportions of 25–49-year-olds reporting being screened for cervical precancer by age groups, rural versus urban residence, education and wealth quintile by country (percentage, 95% CI) (PHIA 2015–2019).

Figure 1 shows the proportion of respondents reporting screening for cervical precancer by age group (25–34 vs 35–49-year-olds), residence (rural vs urban), education level and wealth quintile by country and table 2 presents adjusted ORs. In all countries except Zambia, more 35–49-year-olds than 25–34-year-olds reported screening for cervical precancer (figure 1). Age-related differences in reported screening varied and ranged in percentage points from 0.2% (1.7% among 25–34-year-olds vs 1.9% among 35–49-year-olds) in Rwanda to 2.8% (7.3% among 25–34-year-olds vs 10.1% among 35–49-year-olds) in Zimbabwe. After adjusting for covariates, 35–49-year-olds in all countries surveyed reported being more likely to be screened for cervical precancer than 25–34-year-olds (table 2).

**Table 2:** Survey weighted differences in self-reported cervical precancer screening among 25–49 years old by age groups, rural–urban location, education and wealth quintile by country—(ORs((95% CI)) (PHIA 2015–2019)

Inequality dimensions	Ethiopia	Malawi	Rwanda	Tanzania	Zambia	Zimbabwe
Age (years) 35–49 (ref 25–34)	1.90 (1.48 to 2.43)	1.77 (1.39 to 2.27)	1.46 (1.11 to 1.93)	2.32 (1.85 to 2.91)	1.50 (1.28 to 1.75)	2.31 (1.92 to 2.78)
Residence urban (ref: rural)	1.65 (1.02 to 2.65)	1.91 (1.29 to 2.82)	1.67 (1.05 to 2.65)	1.57 (1.03 to 2.41)	1.27 (0.96 to 1.68)	0.90 (0.65 to 1.24)
Education primary (ref: no education)	0.97 (0.60 to 1.54)	1.04 (0.74 to 1.48)	1.84 (1.04 to 3.26)	0.91 (0.61 to 1.36)	1.45 (0.97 to 2.16)	0.78 (0.44 to 1.38)
Secondary	1.32 (0.77 to 2.29)	1.69 (1.11 to 2.58)	3.11 (1.69 to 5.71)	1.19 (0.70 to 2.00)	1.81 (1.19 to 2.75)	1.10 (0.63 to 1.94)
Higher	1.73 (1.02 to 2.93)*	2.10 (1.28 to 3.44)	2.08 (0.99 to 4.38)	1.38 (0.71 to 2.67)	2.52 (1.61 to 3.94)	1.95 (1.05 to 3.60)
Wealth quintiles						
Q2 (ref: lowest Q1)	0.92 (0.46 to 1.85)	1.42 (0.85 to 2.36)	1.00 (0.60 to 1.66)	1.39 (0.78 to 2.49)	0.98 (0.61 to 1.58)	1.38 (1.00 to 1.91)
Q3	1.63 (0.75 to 3.51)	1.57 (0.97 to 2.55)	1.32 (0.81 to 2.15)	1.72 (1.07 to 2.77)	2.24 (1.51 to 3.34)	2.04 (1.47 to 2.85)
Q4	1.78 (0.80 to 3.97)	2.16 (1.32 to 3.53)	1.25 (0.77 to 2.04)	1.93 (1.08 to 3.44)	2.40 (1.54 to 3.74)	3.24 (2.19 to 4.82)
Q5 highest quintile	2.13 (0.96 to 4.75)	3.53 (2.15 5.80)	1.34 (0.74 to 2.43)	2.44 (1.31 to 4.54)7	4.01 (2.51 to 6.40)	4.95 (3.17 to 7.70)
Goodness-of-fit Prob>F	0.4317	0.9611	0.9176	0.9082	0.9569	0.9791
	F (9, 17) = 1.07	F (9, 17) = 0.31	F (9, 17) = 0.40	F (9, 17) = 0.42	F (9, 17) = 0.32	F (9, 17) = 0.25
N	5850	6053	8553	9167	6375	6777

Pearson test with jack-knife 95% CIs for cell proportions, n=number of observations. Significance was set at p=0.05. Goodness-of-fit test (Prob>). Ethiopia data set only had an urban variable. We classified an urban population size <50 000 as rural and >50 000 as urban. The regressions also controlled for the dummy variables province for Rwanda, Zambia and Zimbabwe; region for Ethiopia; zone for Malawi; and mainlandzanziba for Tanzania. The results are not included. Q 1—lowest wealth quintile, Q 2, Q 3, Q 4, Q 5—highest wealth quintile.

*Not significant after correcting for multiple hypothesis testing with the sharpened Qs.

N, number of observations; PHIA, Population-Based HIV Impact Assessment.

More urban residents than rural residents reported screening for cervical precancer in Ethiopia, Tanzania, Zambia and Zimbabwe before adjusting for covariates (figure 1). In these countries, the rural–urban inequalities in cervical precancer screening were low, except in Zambia (5.1%, moderate). After adjustment, more urban residents than rural residents in Ethiopia, Malawi, Rwanda and Tanzania reported screening for cervical precancer (table 2).

Respondents in Malawi, Rwanda, Zambia and Zimbabwe had education-related inequalities (figure 1). Education-related inequalities were also low in these countries except Zambia, where it was moderate between the uneducated and those with secondary schooling (5.9%). Respondents in Malawi, Tanzania, Zambia and Zimbabwe had significant wealth-related inequalities in cervical precancer screening (figure 1 and table 2). In Malawi and Tanzania, the inequalities between respondents in the lowest and highest wealth quintiles were low—4.9% and 1.9%, respectively. They were moderate in Zambia (5.7%) and Zimbabwe (5.3%) (figure 1). The goodness-of-fit test showed that our models fit the data well (table 2).

**Figure 2:**
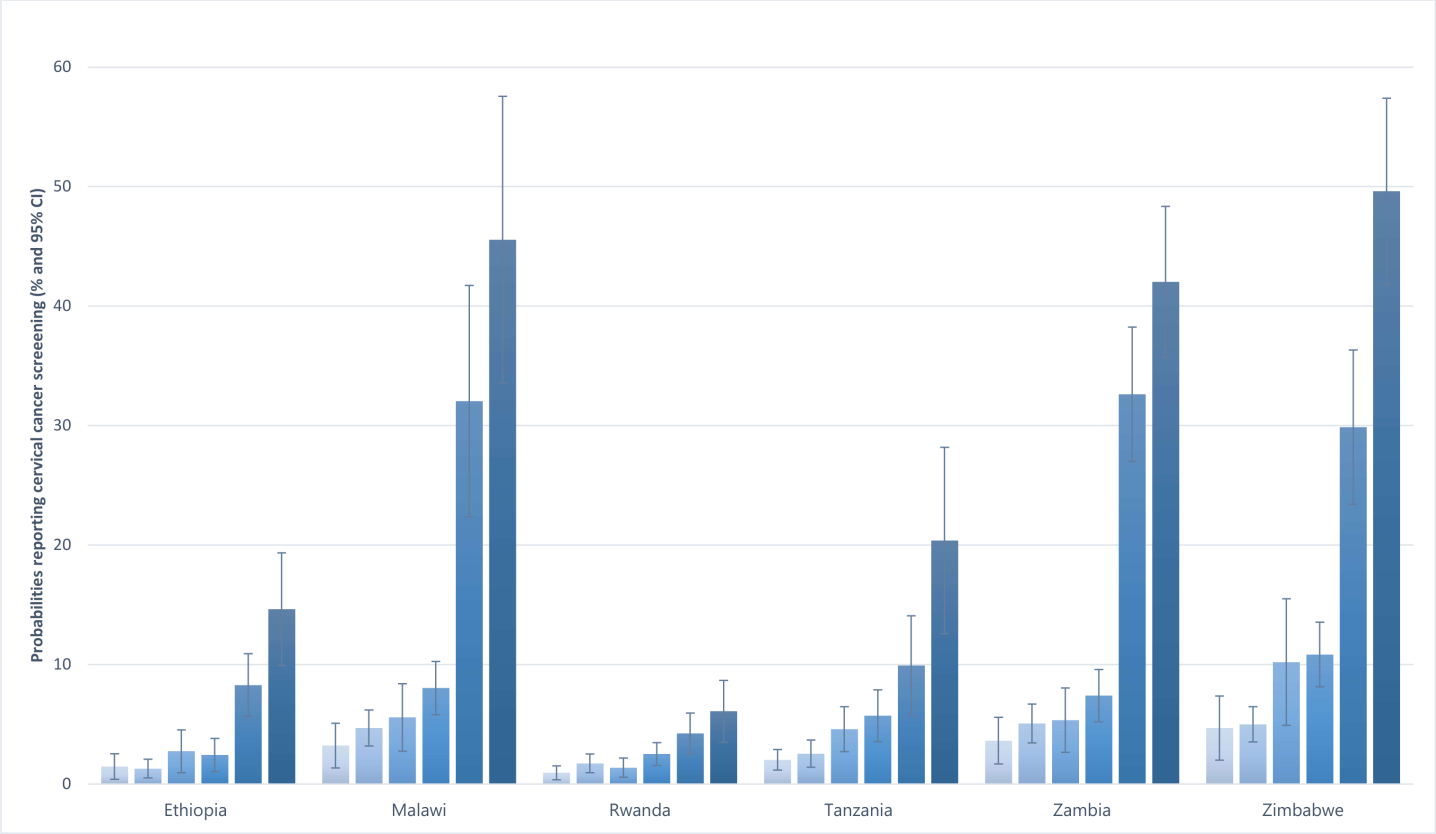
Survey weighted differences in probabilities of self-reported cervical precancer screening by combinations of age group, rural–urban residence, no education, higher education and lowest to highest wealth quintiles by country. Marginal effects (percentages, 95% CI) (PHIA 2015–2019). PHIA, Population-Based HIV Impact Assessment. 1=lowest wealth quintile, 2, 3, 4, 5=highest wealth quintile. 25-34# = 25 - 34-year-olds; 35-49# = 35 - 49-year-olds. #Rural=rural. #Rich=rich. Noed=no education, Prim=primary, High=higher education. PHIA, Population-Based HIV Impact Assessment.

Figure 2 shows the probabilities of self-reported cervical precancer screening for selected combinations of age group (25–34, 35–49-year-olds), residence (rural, urban), education level and wealth quintile in percentage points by country. There were significant differences in cervical screening probabilities between the rural residents in the lowest wealth quintile with no education aged 25–34 years (25–34#Rural#Noed#1 in figure 2) and their urban residents peers in the highest wealth quintile, highest educated (25–34#Urban#High#5) in figure 2, ranging from 3.3% in Rwanda to 29.0% in Zambia. When similar comparisons were made for 35–49-year-olds, the differences ranged from 3.6% in Rwanda to 38.8% in Zimbabwe. A comparison of rural residents aged 25–34 years old, with primary education in the second wealth quintile (25–34#Rural#Prim#2) and urban residents aged 35–49 years old, with the highest education in the highest wealth quintile (35–49#Urban#High#5), showed substantially increased differences, ranging from 4.4% in Rwanda to 44.6% in Zimbabwe. Inequalities between urban residents aged 25–34 years old, with the highest education, in the highest wealth quintile (25–34#Urban#High#5) and urban residents aged 35–49 years old, with the highest education, in the highest wealth quintile (35–49#Urban#High#5) were moderate in magnitude, except in Rwanda where they were low (figure 2).

## Discussion

This study examined cervical precancer screening inequalities related to age, residence, education and wealth in six sub-Saharan African countries—Ethiopia, Malawi, Rwanda, Tanzania, Zambia and Zimbabwe. We found that self-reported cervical precancer screening was low and inequitable. None of the countries reached one-third of 70% WHO cervical precancer screening target, 8 years away from the 2030 deadline. Self-reported cervical precancer screening rates ranged from 3.5% (95% CI 3.1% to 4.0%) in Rwanda to 17.1% (95% CI 15.8% to 18.5%) in Zambia and Zimbabwe (17.4% (95% CI 16.1% to 18.8%). However, women who were older, residing in urban areas, who were more educated and in the highest wealth quintile reported a higher uptake of cervical precancer screening than women who were younger, living in rural areas, who were uneducated and in the lowest wealth quintile. Age-related inequalities were the most common—observed in all six surveyed countries. Rural–urban, education and wealth-related inequalities were each observed in four countries, ranging from low to moderate in magnitude. We also observed high inequalities from combinations of inequalities in age group, residence (rural, urban), education level and wealth in a few countries. These results have policy implications for the rapid scale-up of equitable life-saving cervical precancer screening.

In this study, self-reported cervical precancer screening was lower than one-third of the WHO target of screening 70% of eligible women for cervical precancer by 2030, consistent with other studies conducted in the surveyed countries. Studies found that cervical precancer screening uptake ranged from 11% in Tanzania to 26.5% in Malawi, despite differences in methodologies used to estimate cervical precancer screening.^9 10^ Countries should urgently scale up cervical precancer screening to meet the WHO cervical precancer screening target, removing inequalities that prevent some women from accessing screening for cervical precancer, and integrate cervical precancer screening in maternal, HIV, sexual and reproductive health, and social protection programmes.

Evidence supports the finding that inequalities in these underserved cervical precancer screening countries favoured women who were older, resided in urban areas, were more educated and in the highest wealth quintile than women who were younger, who lived in rural areas, were uneducated and in the lowest wealth quintile.^9 17 18^ Studies conducted in Ethiopia, Rwanda, Tanzania, Zambia and Zimbabwe, also support this finding.^10–15 34–36^ A study of 18 resource-constrained countries, of which eight were from sub-Saharan Africa further found that wealth status increased socioeconomic inequalities in cervical precancer screening, whereas being married, unemployed and living in urban communities reduced it.^9^ The results of this study agree with existing evidence on socioeconomic inequalities in maternal health. A study in Malawi found that rural–urban residence, education and wealth status significantly impacted the uptake of three types of maternal health services in the study population. It found that compared with women residing in rural areas, who had lower education and were in the poorest wealth quintile, women living in urban areas, who had higher education and were in the higher wealth quintiles had significantly higher odds of receiving four antenatal care visits, skilled birth attendance and postnatal care.^37^ A study from Zambia reported similar findings.^38^ So did a study from 36 sub-Saharan African countries, concluding that prioritising quality education of women in sub-Saharan Africa would reduce disparities in antenatal service utilisation.^39^ Countries should urgently scale-up cervical precancer screening, addressing inequalities that prevent some women from accessing services.

This study also determined the magnitude of the socioeconomic inequalities in reported cervical precancer screening to aid policy-making in the prioritisation of inequalities to eliminate in the scale-up of cervical precancer screening. We found low to moderate age, rural–urban, education and wealth-related inequalities in cervical precancer screening in the surveyed countries. Age-related inequalities were reported in all the surveyed countries and residence, education and wealth-related inequalities in four of the six surveyed countries. Although the inequalities were low to moderate, our study found that their combined effects on cervical precancer screening probabilities were substantial, especially in countries with higher rates of self-reported cervical precancer screening. The inequalities in cervical precancer screening rates between rural residents aged 25-34, with no education in the lowest wealth quintile, and their urban resident peers, with highest education in the highest wealth quintile, ranged from low inequalities in Rwanda, to moderate inequalities in Ethiopia and Tanzania to high inequalities in Malawi, Zambia and Zimbabwe. Comparing primary educated rural residents aged 25-35 years old, in the lowest wealth quintile with the highest educated urban residents aged 35–49 years old, in the highest wealth quintile showed substantial differences. Inequalities did not disappear between the 25–34 years old most affluent, highest educated, urban residents, and 35–49 years old, most affluent, highest educated, urban residents. These results suggest that inequalities persist. Minor or moderate inequalities compound into high inequalities, further leaving women behind in cervical precancer screening. Starting with age-related inequalities, eliminating socioeconomic inequalities should be prioritised in the urgent scale-up of cervical precancer screening interventions.

Comparing the proportion of HIV-positive women in the sample and their reported cervical precancer screening showed cervical precancer screening gaps among HIV-positive women. In our study, the proportion of HIV-positive 25–49 years old women ranged from 4.7% (95% CI 4.1% to 5.4%) in Rwanda to 22.6% (95% CI 21.6% to 23.7%) in Zimbabwe. By contrast, self-reported cervical precancer screening among this population ranged from 0.4% (95% CI 0.3% to 0.5%) in Rwanda to 5.4% (95% CI 4.7% to 6.1%) in Zambia. Not all HIV-positive women were screened for cervical precancer for several reasons. One reason may be the perception that linking women testing HIV-positive to HIV treatment eliminates the risk of cervical precancer for such women. A study in Zimbabwe found that many women living with HIV on ART screened positive for cervical precancer, despite the country attaining HIV treatment targets.^40^ Another reason women may have not been screened for cervical precancer is that they may have tested HIV-positive at a facility with a limited capacity to conduct cervical precancer screening. Alternatively, women might have been unable to access health facilities with the capacity to test for cervical precancer for various reasons, including transport costs. A large proportion of HIV-negative women who were tested for HIV in this study were not screened for cervical precancer. One reason is the perception that HIV-negative women may be at low risk of cervical precancer.^14^ Integration between HIV testing and cervical precancer screening is required and is feasible in the scale up of cervical precancer screening.^41^ Increased resources for HIV testing, training and orientation of healthcare staff to screen eligible women for cervical precancer, including HIV-negative women, is required to scale up screening services and reduce inequalities in the uptake of services.

Besides intensifying cervical precancer screening in HIV programmes, governments should fulfil their obligations to the right to sexual and reproductive health by increasing the accessibility of their health systems.^8^ They should reorient the building blocks of health systems—governance, financing, human resources, information, medical technologies and service delivery—towards addressing inequalities and reaching all eligible women.^8 42^ They should establish, fund and scale-up population-level public-sector screening and treatment services and integrate them into HIV, STI, maternal and reproductive health services.^43^ Governments could build cervical precancer screening around a target population, such as eligible schoolgirls and college students, which would assist in addressing socioeconomic inequalities. For example, Rwanda vaccinated more than 90% of schoolgirls against HPV from 2011 to 2012, through designated ‘health days’ adapted for other sexual reproductive and maternal health purposes.^44 45^ This approach reduced time, money and other costs of accessing HPV vaccination for girls, and other maternal health services for women.^44^ In contrast to other countries surveyed in this study, none of the wealth levels were associated with cervical precancer screening in Rwanda. However, the cervical precancer screening rate in Rwanda in this study was low at 3.5% (3.1%–4.0%), illustrating that cervical precancer screening did not scale-up as did the HPV vaccination.^45^ Additional activities may be added to such programmes to increase access and reduce inequalities. These include multiple screening over the lifetime, reducing the perception of high economic costs associated with cervical precancer screening, and instituting same-day cervical precancer screening and treatment.^46^ Other recommendations are the removal of economic barriers and focused outreach to women less likely to be screened and allowing them to self-collect samples.^1 15 46^ Women’s ability to benefit from these programmes may depend on their social health protection, such as whether they have childcare responsibilities that prevent them from accessing services.^21^ Women with multiple inequalities depend on social protection and may be vulnerable under systems with low social protection.^21^ Governments should expand their social protection coverage and enhance their health system accessibility to scale up cervical precancer screening, addressing the socioeconomic inequalities that prevent women from accessing services.^21^

This study has several limitations. Collinearity was present, especially between the wealth and residence variables which tend to correlate with each other, consistent with how the wealth variable is constructed. Recall bias was present as cervical precancer screening is self-reported. We did not control for the type of cervical precancer screening because we did not have information on the types of screening women received. Cervical precancer screening uptake may have worsened because of the COVID-19 pandemic. We did not determine if women had received two screenings by age 45 years as recommended by WHO, which could change our results. However, we used high-quality population-based data providing useful study findings to countries in sub-Saharan Africa.

Cervical precancer screening in the surveyed countries was inequitable and low—less than one-third of the WHO target to screen 70% of eligible women by 2030 for cervical precancerous lesions. Women who were younger, lived in rural areas, had no education and were from poor backgrounds were left behind in cervical precancer screening. Age-related inequalities were the most common type of inequalities observed. In combination, inequalities related to age, residence, education and wealth left further behind women who were younger, resided in rural areas, were uneducated, and poor. Focused and inclusive national cervical precancer interventions are required to scale-up equitable cervical precancer screening. Governments should include and monitor equity in their cervical precancer screening programmes. Research is needed to understand how to scale-up equitable cervical precancer screening in sub-Saharan Africa countries.

## Supplementary Material

Reviewer comments

Author's manuscript

## Data Availability

Data are available in a public, open access repository. The data are deidentified and publicly available at https://phia-data.icap.columbia.edu/files.
