# Supplementary material for: Socioeconomic inequalities in cervical precancer screening among women in Ethiopia, Malawi, Rwanda, Tanzania, Zambia and Zimbabwe: analysis of Population-Based HIV Impact Assessment surveys
Source: BMJ Open. 2023 Jun 20;13(6):e067948. doi: 10.1136/bmjopen-2022-067948 (PMC10314495; doi:10.1136/bmjopen-2022-067948)
Supplement: Supplementary data [file bmjopen-2022-067948supp001.pdf]

Supplementary Table 1. Variable descriptions.

Variable name	Description	Measure
Cervical pre-cancer screening	Self-reported from the adult interview question on ever testing for cervical pre-cancer, coded no, yes.	Binary
Sex	Self-reported sex (i.e., male, female).	Binary
Age	Self-reported linear years categorized in years (25–34, 35–49).	Categorical
Location, rural, urban	Self-reported residence (i.e., rural, urban), from the household interview. The data from Ethiopia had only urban variables. For Ethiopia, we coded size of area <=50,000 as rural = 0, and >50k =1; urban, from the household data set.	Binary
Ever married	Was derived from the question 'Have you ever been married or lived together with a (man/woman) as if married?' coded no, yes.	Binary
Ever tested for HIV	From the question 'Has been tested for HIV', coded never tested, ever tested.	Binary
HIV prevalence	The proportion of people 15 years or older testing HIV positive using laboratory-confirmed HIV test results from the adult biomarker questionnaire.	Binary
Educational status	Level of school respondent ever attended (i.e., not educated, primary school education, secondary school education, or higher education, from the adult interview. Tanzania's educations variables were standardized as follows: No education; Primary combined pre-primary, primary and post-primary; Secondary combined secondary, post-secondary training, secondary A); More than secondary (included post-secondary A levels and university).	Categorical
Wealth	Assessed household wealth with the wealth quintiles variable, which was derived from a wealth index. Wealth quintiles ranked households depending on their household characteristics and asset ownership from Q1 representing the poorest households to Q5 representing the wealthiest. (i.e., poorest, poor, rich, richer, and richest). The variable is provided in the PHIA household data set.	Ordered ranking
Regions, province, or zone	Defined as a political area, which is the primary sampling unit. It was province for Rwanda, Zambia, and Zimbabwe; region for Ethiopia, zone for Malawi, and mainlandzanziba for Tanzania.	Categorical

Note: Unless indicated the variables were from the adult interview data set. The answer options 'didn't know' or 'refused to answer' were coded as missing for all variables.

Chipanta D, *et al. BMJ Open* 2023; 13:e067948. doi: 10.1136/bmjopen-2022-067948
