# Supplementary material for: Socioeconomic inequalities in cervical precancer screening among women in Ethiopia, Malawi, Rwanda, Tanzania, Zambia and Zimbabwe: analysis of Population-Based HIV Impact Assessment surveys
Source: BMJ Open. 2023 Jun 20;13(6):e067948. doi: 10.1136/bmjopen-2022-067948 (PMC10314495; doi:10.1136/bmjopen-2022-067948)
Supplement: Author's manuscript [file bmjopen-2022-067948.draft_revisions.pdf]

BMJ Open is committed to open peer review. As part of this commitment we make the peer review history of every article we publish publicly available.

When an article is published we post the peer reviewers' comments and the authors' responses online. We also post the versions of the paper that were used during peer review. These are the versions that the peer review comments apply to.

The versions of the paper that follow are the versions that were submitted during the peer review process. They are not the versions of record or the final published versions. They should not be cited or distributed as the published version of this manuscript.

BMJ Open is an open access journal and the full, final, typeset and author-corrected version of record of the manuscript is available on our site with no access controls, subscription charges or pay-per-view fees (http://bmjopen.bmj.com).

If you have any questions on BMJ Open's open peer review process please email info.bmjopen@bmj.com

BMJ Open

Socioeconomic inequalities in cervical pre-cancer screening among women in Ethiopia, Malawi, Rwanda, Tanzania, Zambia, and Zimbabwe – Analysis of Population-Based HIV Impact Assessment surveys.

Journal:	*BMJ Open*
Manuscript ID	bmjopen-2022-067948
Article Type:	Original research
Date Submitted by the Author:	02-Sep-2022

Complete List of Authors:	Chipanta, David; University of Geneva, Faculty of Medicine; UNAIDS, ERA<br>Kapambwe, Sharon; World Health Organisation<br>Nyondo-Mipando, Alinane Linda; Kamuzu University of Health Sciences<br>Pascoe, Margaret; Newlands Clinic, Clinic<br>Amo-Agyei, Silas; University of Lausanne, Department of Economics<br>Bohlius, Julia; University of Bern, Swiss Tropical and Public Health Institute<br>Estill, Janne; University of Geneva<br>Keiser, Olivia ; University of Geneva

Keywords:	Cancer pain < ONCOLOGY, HIV & AIDS < INFECTIOUS DISEASES, PUBLIC HEALTH, Health policy < HEALTH SERVICES ADMINISTRATION & MANAGEMENT

SCHOLARONE™
Manuscripts

I, the Submitting Author has the right to grant and does grant on behalf of all authors of the Work (as defined in the below author licence), an exclusive licence and/or a non-exclusive licence for contributions from authors who are: i) UK Crown employees; ii) where BMJ has agreed a CC-BY licence shall apply, and/or iii) in accordance with the terms applicable for US Federal Government officers or employees acting as part of their official duties; on a worldwide, perpetual, irrevocable, royalty-free basis to BMJ Publishing Group Ltd ("BMJ") its licensees and where the relevant Journal is co-owned by BMJ to the co-owners of the Journal, to publish the Work in this journal and any other BMJ products and to exploit all rights, as set out in our licence.

The Submitting Author accepts and understands that any supply made under these terms is made by BMJ to the Submitting Author unless you are acting as an employee on behalf of your employer or a postgraduate student of an affiliated institution which is paying any applicable article publishing charge ("APC") for Open Access articles. Where the Submitting Author wishes to make the Work available on an Open Access basis (and intends to pay the relevant APC), the terms of reuse of such Open Access shall be governed by a Creative Commons licence – details of these licences and which Creative Commons licence will apply to this Work are set out in our licence referred to above.

Other than as permitted in any relevant BMJ Author's Self Archiving Policies, I confirm this Work has not been accepted for publication elsewhere, is not being considered for publication elsewhere and does not duplicate material already published. I confirm all authors consent to publication of this Work and authorise the granting of this licence.

For peer review only

Socioeconomic inequalities in cervical pre-cancer screening among women in Ethiopia, Malawi, Rwanda, Tanzania, Zambia, and Zimbabwe – Analysis of Population-Based HIV Impact Assessment surveys.

David Chipanta[1,2] *, Sharon Kapambwe[3], Alinane Linda Nyondo-Mipando[4], Margaret Pascoe[5] Silas Amo-Agyei[6], Julia Bohlius[78], Janne Estill[1], Olivia Keiser[1]

1 Institute of Global Health, University of Geneva, Geneva, Switzerland.
2 Joint United Nations Programme on HIV/AIDS (UNAIDS), Geneva, Switzerland.
3 World Health Organisation, Harare, Zimbabwe.
4 School of Global and Public Health, Kamuzu University of Health Sciences, Blantyre, Malawi.
5 Newlands Clinic, Harare, Zimbabwe.
6 Department of Economics, University of Lausanne, Lausanne, Switzerland.
7 Swiss Tropical and Public Health Institute, Basel, Switzerland.
8 University of Basel, Basel, Switzerland

*Corresponding author: Email: chipanta@hotmail.com; david.chipanta@etu.unige.ch

E-mail addresses of co-authors:
SK: kapambwes@who.int
ALNM- lmipando@kuhes.ac.mw
MP MargaretP@newlandsclinic.org.zw
SA: silas.amo-agyei@unil.ch
JB julia.bohlius@swisstph.ch
JE: Janne.Estill@unige.ch
OK: Olivia.Keiser@unige.ch

Where authors are identified as personnel of the International Agency for Research on Cancer / World Health Organization, or UNAIDS, the authors alone are responsible for the views expressed in this article and they do not necessarily represent the decisions, policy, or views of the International Agency for Research on Cancer / World Health Organization or UNAIDS.

For peer review only - http://bmjopen.bmj.com/site/about/guidelines.xhtml

Abstract

Objective: This study aimed to examine age, residence, education and wealth inequalities and their combinations on cervical pre-cancer screening for women.

Designs and setting: This cross-sectional study used Population-Based HIV Impact Assessment data for Ethiopia, Malawi, Rwanda, Tanzania, Zambia, and Zimbabwe. Differences in cervical pre-cancer screening were analysed using multivariable logistic regressions, controlling for age, residence, education, and wealth. Inequalities in cervical pre-cancer screening probability were estimated using marginal effects models.

Participants: Women aged 25–49 years.

Main outcomes: The main outcomes were self-reported cervical pre-cancer screening rates, and their inequalities in percentages points. We defined differences of 20%+ as high inequality, 5% to 20% medium, 0 to 5% as low.

Results: The sample size of participants ranged from 5882 in Ethiopia to 9186 in Tanzania. Self-reported cervical pre-cancer screening was low in the surveyed countries ranging from 3.5% (95% Confidence Interval (CI) 3.1%–4.0%) in Rwanda to 17.1% (95% CI 15.8%–18.5%) and 17.4% (95% CI16.1%–18.8%) in Zambia and Zimbabwe. Inequalities in cervical pre-cancer screening were low based on covariates. Combining the inequalities led to significant inequalities in self-reported cervical pre-cancer screening between rural dwelling women aged 25–34 years, in the lowest wealth quintile, with a primary education level, and urban dwelling women aged 35–49 years, in the highest quintile, with the highest education level ranging from 4.4% in Rwanda to 44.6% in Zimbabwe.

Conclusions: Cervical pre-cancer screening was inequitable and low. None of the countries achieved one-third of the World Health Organisation target of screening 70% of eligible women for cervical pre-cancer by 2030. Inequalities in cervical pre-cancer screening were low. Their combinations led to high inequalities, preventing younger, rural-dwelling, uneducated, women in the lowest wealth quintile from cervical pre-cancer screening. Governments should include and monitor equity in their cervical pre-cancer screening programmes.

Strength and limitations of this study

- Collinearity was present, especially between the wealth and residence variables which tend to correlate with each other, consistent with how the wealth variable is constructed.
- Recall bias was present as cervical pre-cancer screening is self-reported.
- We did not control for the type of cervical pre-cancer screening because we did not have information on the types of screening women received.

For peer review only - http://bmjopen.bmj.com/site/about/guidelines.xhtml

For peer review only

Introduction

Although all countries are affected by cervical pre-cancer, its incidence and mortality are more than twice and three times as high in low- and middle-income countries as in high-income countries (1). In 2020, it was the leading cause of cancer deaths in 36 countries. Most of these countries were in sub-Saharan Africa, Melanesia, South America, and South-Eastern Asia (2). Cervical cancer occurs in the lower part of the uterus that connects to the vagina. It is caused in over 70% of cases by the human papillomavirus (HPV), the most common sexually transmitted infection (STI) (1). Most HPV strains are harmless in people with a healthy immune system (1). HIV-positive women, whose immunity may be compromised (1,2) are six times more likely to develop cervical cancer than HIV negative women (1). In 2014, invasive cervical cancer was 11 times higher among women living with HIV in South Africa than in Europe five years after antiretroviral therapy (ART) initiation (3). Cervical cancer harms girls, women and their families with significant health and development consequences, yet it is preventable and curable if detected and treated early (1,2). HPV vaccination and regular pre-cancer screening stop cervical pre-cancer. Cervical pre-cancer screening identifies pre-cancerous cells to treat before they become pre-cancer (1). Comprehensive HPV vaccination and cervical pre-cancer screening could prevent an estimated 5.2 million cases and 3.7 million deaths over 10 years and would cost around US $3.2 billion in 50 low and lower-middle-income countries (4). In 2020, the World Health Organisation (WHO) proposed the 90–70–90 target, which, if achieved by 2030, would put the world on course to eliminate cervical cancer (1). The 90–70–90 target seeks to ensure that 90% of girls are vaccinated against HPV by age 15 years, 70% of eligible women are screened by age 35 years and again by 45, and 90% of women identified with cervical cancer receive treatment (1).

For peer review only - http://bmjopen.bmj.com/site/about/guidelines.xhtml

Cervical cancer is a disease of inequality. Its burden reflects inequalities in society, including those related to socioeconomic status. Inequality in health and health care is defined as the unequal distribution of health goods, services and outcomes across income or other measures of wealth. In contrast, equity is the lack of avoidable and remedial differences in the distribution of health goods and services or outcomes due to people's socioeconomic status in society (5,6,7). Cervical pre-cancer screening is a critical component of the 90–70–90 target (8). Socioeconomic inequalities are preventing women from accessing cervical pre-cancer screening. Studies show that residence, geography, education, wealth, age, health insurance status, and the capacity of health systems influence access to cervical pre-cancer screening. Other factors include, a history of multiple sexual partners, HIV-positive status and women's social interactions (9,10,11,12,13,14,15,16,17,18). These studies showed that women who were older, resided in urban areas, and were more educated and wealthier were screened more often than younger, rural residents, uneducated, and poor women, with a few exceptions (9,19,20,21,22). However, these studies did not estimate the magnitude of inequalities and their combined impact on cervical pre-cancer screening. Such studies are needed to scale up cervical pre-cancer screening and support the prioritization of inequalities to eliminate cervical cancer, especially in east and southern African countries, where HIV has worsened the cervical cancer burden (23). This study aimed to examine age, residence, education, and wealth inequalities and their combined impacts on cervical pre-cancer screening in Ethiopia, Malawi, Rwanda, Tanzania, Zambia, and Zimbabwe. We hypothesized that significant inequalities in cervical pre-cancer screening rates would favour older women, women who resided in urban areas, and more educated and wealthy women.

Methods

Data and sample

For peer review only - http://bmjopen.bmj.com/site/about/guidelines.xhtml

This is cross-sectional study of a secondary data analysis of available PHIA data for countries that included cervical pre-cancer screening variables. The countries and the years in which the PHIA surveys were conducted include Ethiopia (2017–2018), Malawi (2015–2016), Rwanda (2018), Tanzania (2016–2017), Zambia (2016) and Zimbabwe (2015–2016). The PHIA surveys collected a range of health and socio-demographic data to evaluate HIV the impact of HIV programs in countries supported by United States President's Emergency Plan for AIDS Relief (PEPFAR). We used the Household, and Adult data sets. In participating households, a household questionnaire was administered to the head-of-household, who provided information on the household relating to wealth, and other socio demographic characteristics. Then, an individual questionnaire was administered to eligible and consenting adults aged 15 years or older in the household. The individual interviews assessed a range of HIV-related variables, including cervical pre-cancer screening and HIV testing.

We restricted our analysis to women aged 25–49 years who responded to the cervical pre-cancer questions. This is the group WHO recommends for cervical pre-cancer screening – ages 30–49 years for the general population and 25–49 years for women living with HIV – including all gender diverse people at risk of cervical cancer (1).

Patient and Public Involvement

No patient involved.

Variables and outcome descriptions

We assessed inequalities related to age, residence, education, and wealth in cervical pre-cancer screening according to women’s self-reports. These dimensions of inequalities are

For peer review only - http://bmjopen.bmj.com/site/about/guidelines.xhtml

familiar sources of disadvantage or discrimination (24). Our primary outcome was self-reported ever testing for cervical pre-cancer. The main predictors were age, residence, education, and wealth. Other covariates were having ever married, ever been tested for HIV, (also binary coded), and regions. Regions, provinces, or zones were coded as dummy variables and included to capture the geographical variation in cervical pre-cancer screening. Ethiopia's data set had an urban variable, which we coded as rural if the population size was smaller than 50,000 people, and urban otherwise. Supplementary Table 1 describes the variables. We used percentages to measure the magnitude of inequality between subgroups. We denoted a difference of 20% or more between two subgroups as high inequality, less than 20% but greater than 5% as medium inequality and below 5% as low inequality, as used by the WHO (24).

Analysis

We assessed inequalities in self-reported cervical pre-cancer screening in three steps. For each country, we first presented sample characteristics descriptively. Second, we determined the associations between screening for cervical pre-cancer and each dimension of inequality, and analysed the differences using multivariable logistics regression, controlling for covariates. We corrected the p-values of odds ratios for multiple hypothesis testing with sharpened q's. In step 3, we used marginal effects models to estimate the probabilities of reporting cervical pre-cancer screening for each inequality alone and combined with other inequalities, holding the other inequalities constant at their mean values. We applied survey weights to account for complex survey design. Using jack-knife replicate weights, 95% confidence intervals (CI) were estimated (25). We excluded the observation from the analysis if the gender value was missing. We did not impute the missing data since all variables had less than 2% of missing values. We used Stata version 14 for the analyses. The study did not require ethical clearance. The data are publicly available and de-identified at https://phia-

For peer review only - http://bmjopen.bmj.com/site/about/guidelines.xhtml

data.icap.columbia.edu/files (26). This study conforms to the SQUIRE reporting guidelines (27).

Results

The sample size of females aged 25–49 years ranged from 5882 in Ethiopia to 9186 in Tanzania. The median age and interquartile ranges were 32 years (28–39) in Ethiopia, 33 years (29–40) in Zambia and 34 years (29–40) in Malawi, Rwanda, and Tanzania. The rate of self-reported cervical pre-cancer screening was lowest in Rwanda (3.5% [95% CI 3.1%–4.0%]) and highest in Zambia (17.1% [95% CI 15.8%–18.5%]) and Zimbabwe (17.4% [95% CI 16.1%–18.8%]). The HIV prevalence among 25–49-year-old females ranged from 4.7% (95% CI 4.1%–5.4%) in Rwanda to 22.6% (95% CI 21.6%–23.7%) in Zimbabwe, and the rate of cervical pre-cancer screening among HIV-positive females ranged from 0.4% (95% CI 0.3%–0.5%) in Rwanda to 5.4% (95% CI 4.7%–6.1%) in Zambia. Females aged 25 to 34 years comprised half or more of the sample in surveyed countries. Eighty-six percent or more of the respondents reported ever have been married, and 86% or more reported ever have been tested for HIV. More than half of the respondents resided in rural areas, excluding Ethiopia, where all respondents resided in urban areas, 54% of them in areas with <50,000 residents. Less than 20% of the respondents were uneducated, and nearly 30% had attained a primary school education. Only in Ethiopia, Malawi and Zambia was the proportion of respondents in the top two wealth quintiles greater than that in the bottom two quintiles (Table 1).

For peer review only - http://bmjopen.bmj.com/site/about/guidelines.xhtml

Table 1: Survey weighted sample characteristics by country (PHIA 2015–2018), (percentage [95% Confidence Interval] sample size)

	Ethiopia	Malawi	Rwanda	Tanzania	Zambia	Zimbabwe
**CC screened**	6.1 (5.3–6.9),343	11.8 (10.6–13.1),926	3.5 (3.1–4.0),353	7.0 (6.1–8.1),586	17.1 (15.8–18.5),1066	17.4 (16.1–18.8),1129
**HIV positive**	6.1 (5.3–7.0),362	18.8 (17.3–20.3),1152	4.7 (4.1–5.4),416	9.2 (8.4–10.0),897	21.2 (19.8–22.6),1263	22.6 (21.6–23.7),1602
Cervical Pre-cancer screened	0.9 (0.7–1.45),52	3.4 (2.8–4.1), 249	0.4 (0.3–0.5),43‡	1.5 (1.1 –2.0),132	5.4 (4.7–6.1),318	4.5 (4.0–5.0),305
**Age** (years): Median [IQR] years	32 (28–39)	34 (29–40)	34 (29–40)	34 (29–40)	33 (29–40)	34 (29-40)
25–34	55.9 (55.7–56.2),3517	53.0 (52.9–53.1),3342	52.1 (52.1–52.2),4508	52.2 (52.2–52.3),4812	55.3 (55.2–55.4),3386	54.5 (54.4–54.6),3403
35–49	44.1 (43.8–44.3),2365	47.0 (46.9–47.1),2720	47.9 (47.8–47.9),4063	47.8 (47.7–47.8),4374	44.7 (44.6–44.8),3027	45.5 (45.4–45.6),3381
**Ever married**	89.7 (88.4–90.9),5228	96.6 (95.9–97.1),5794	86.0 (85.0–87.0),7320	92.6 (91.6–93.6),8565	91.7 (90.6–92.7),5901	94.0 (93.3–94.6),6373
**Ever tested for HIV**	86.4 (84.9–87.7),5018	94.8 (94.0–95.4),5756	95.7 (95.2–96.1),8194	89.2 (88.2–90.1),8206	92.3 (91.5–93.1),5878	94.8 (94.1–95.4),6375
**Location** Urban	54.0 (48.8–59.1),3215	19.0 (16.3–21.9),2289	20.2 (16.5–24.5),2159	39.4 (35.7–43.4),3299	45.9 (42.4–49.4),2867	38.9 (37.0–40.9),2317
**Education:** No education	22.1 (20.0–24.3),1307	14.5 (13.2–15.9),747	11.5 (10.7–12.4),953	17.8 (16.3–19.5),1816	8.5 (7.2–9.9),545	1.8 (1.4–2.3),135
Primary	38.7 (36.7–40.7),2210	66.2 (64.4–68.0),3672	68.1 (66.4–69.7),5666	65.8 (64.2–67.4),5931	51.8 (49.5–54.1),3329	27.6 (26.1–29.2),2044
Secondary	21.1 (19.6–22.7),1230	16.7 (15.3–18.3),1351	15.8 (14.7–16.9),1434	14.7 (13.5–15.9),1297	30.3 (28.4 - 32.3),1953	62.8 (61.1–64.4),4146
Higher	18.1 (16.3–20.0),1122	2.5 (2.1–3.0),288	4.6 (3.9–5.5),510	1.7 (1.3–2.1),137	9.4 (8.0 - 11.1),583	7.8 (6.5–9.2),455
**Wealth quintiles:**						
1, lowest	14.6 (12.5–17.0),910	17.3 (15.7–19.1),748	20.4 (18.3–22.7),1600	18.3 (16.1–20.8),1821	16.3 (14.6–18.1),1055	18.9 (17.3–20.6),1530
2	16.1 (14.5–17.8),972	18.5 (17.0–20.1),863	19.2 (17.5–21.0),1542	19.6 (18.0–21.4),1807	17.5 (16.1–19.0),1140	18.4 (17.2–19.6),1318
3	19.1 (17.6–20.6),1144	19.7 (18.3–21.2),957	19.5 (18.1–21.0),1580	20.0 (18.3–21.8),2063	19.7 (17.9–21.7),1258	17.5 (16.0–19.1),1180
4	23.1 (21.3–25.0),1356	20.7 (19.3–22.2),1207	20.2 (18.7–21.9),1662	20.5 (18.6–22.5),1798	22.2 (20–24.6),1424	21.4 (19.1–23.8),1282
5, highest	27.1 (24.5–29.9),1500	23.7 (21.6–25.9),2287	20.6 (18.1–23.3),2182	21.6 (19.4–24.0),1694	24.3 (21.7–27.1),1506	23.9 (21.4–26.5),1474
**N**	5882	6062	8571	9186	6413	6784

Self-reported cervical pre-cancer screening, proportions in percentages, Pearson's test with jack-knife 95% confidence intervals, N is number of observations. Ethiopia dataset had an urban variable which was divided into population sizes <50,000 and >50,000. We classified a population size of <50,000 as rural and >50,000 as urban. ‡ Estimate based on 25–49 observations and should be interpreted with caution.

For peer review only - http://bmjopen.bmj.com/site/about/guidelines.xhtml

Figure 1 shows the proportion of respondents reporting screening for cervical pre-cancer by age group (25-34 versus 35 – 49-years), residence (rural versus urban), education level, and wealth quintile by country and Table 2 presents adjusted odds ratios. In all countries except Zambia, more 35–49-year-olds than 25–34-year-olds reported screening for cervical pre-cancer (Figure 1). Age-related differences in reported screening varied and ranged in percentage points from 0.2% (1.7% among 25–34-year-olds versus 1.9% among 35 – 49-year-olds) in Rwanda to 2.8% (7.3% among 25–34-year-olds versus 10.1% among 35 – 49-year-olds) in Zimbabwe. After adjusting for covariates, 35–49-year-olds in all countries surveyed reported being more likely to be screened for cervical pre-cancer than 25–34-year-olds (Table 2).

More urban residents than rural residents reported screening for cervical pre-cancer in Ethiopia, Tanzania, Zambia, and Zimbabwe before adjusting for covariates (Figure 1). In these countries, the rural-urban inequalities in cervical pre-cancer screening were low, except in Zambia (5.1%, moderate). After adjustment, more urban residents than rural residents in Ethiopia, Malawi, Rwanda, and Tanzania reported screening for cervical pre-cancer (Table 2).

Respondents in Malawi, Rwanda, Zambia, and Zimbabwe had education-related inequalities (Figure 1). Education-related inequalities were also low in these countries except Zambia, where it was moderate between the uneducated and those with secondary schooling (5.9%). Respondents in Malawi, Tanzania, Zambia, and Zimbabwe had significant wealth-related inequalities in cervical pre-cancer screening (Figure 1 and Table 2). In Malawi and Tanzania, the inequalities between respondents in the lowest and highest wealth quintiles were low – 4.9% and 1.9%, respectively. They were moderate in Zambia (5.7%) and Zimbabwe (5.3%) (Figure 1). The goodness-of-fit test showed that our models fit the data well (Table 2).

For peer review only - http://bmjopen.bmj.com/site/about/guidelines.xhtml

240 [Figure 1 here]

For peer review only

For peer review only - http://bmjopen.bmj.com/site/about/guidelines.xhtml

Table 2: Survey weighted differences in self-reported cervical pre-cancer screening among 25 – 49-year-olds by age-groups, rural-urban location, education, and wealth quintile by country – (odds ratios [95% CI]) (PHIA 2015–2018)

**Inequality dimensions**	**Ethiopia**	**Malawi**	**Rwanda**	**Tanzania**	**Zambia**	**Zimbabwe**
**Age** (years) 35–49 (ref 25–34)	1.90 (1.48–2.43)	1.77 (1.39–2.27)	1.46 (1.11–1.93)	2.32 (1.85–2.91)	1.50 (1.28–1.75)	2.31 (1.92–2.78)
**Residence** Urban (ref: rural)	1.65 (1.02–2.65)	1.91 (1.29 - 2.82)	1.67 (1.05–2.65)	1.57 (1.03–2.41)	1.27 (0.96–1.68)	0.90 (0.65–1.24)
**Education** Primary (ref: no education)	0.97 (0.60 –1.54)	1.04 (0.74–1.48)	1.84 (1.04–3.26)	0.91 (0.61–1.36)	1.45 (0.97–2.16)	0.78 (0.44–1.38)
Secondary	1.32 (0.77–2.29)	1.69 (1.11–2.58)	3.11 (1.69–5.71)	1.19 (0.70–2.00)	1.81 (1.19–2.75)	1.10 (0.63–1.94)
Higher	1.73 (1.02–2.93)	2.10 (1.28–3.44)	2.08 (0.99–4.38)	1.38 (0.71–2.67)	2.52 (1.61–3.94)	1.95 (1.05–3.60)
**Wealth quintiles**: Q2 (ref: lowest Q1)	0.92 (0.46–1.85)	1.42 (0.85–2.36)	1.00 (0.60–1.66)	1.39 (0.78–2.49)	0.98 (0.61–1.58)	1.38 (1.00–1.91)
Q3	1.63 (0.75–3.51)	1.57 (0.97–2.55)	1.32(0.81–2.15)	1.72 (1.07–2.77)	2.24 (1.51–3.34)	2.04 (1.47–2.85)
Q4	1.78 (0.80–3.97)	2.16 (1.32–3.53)	1.25 (0.77–2.04)	1.93 (1.08–3.44)	2.40 (1.54–3.74)	3.24 (2.19–4.82)
Q5 Highest Quintile	2.13 (0.96–4.75)	3.53 (2.15 5.80)	1.34 (0.74–2.43)	2.44 (1.31– 4.54)7	4.01(2.51– 6.40)	4.95 (3.17–7.70)
**Goodness of fit** Prob > F	0.4317	0.9611	0.9176	0.9082	0.9569	0.9791
	F (9,17) = 1.07	F (9,17) = 0.31	F (9,17) = 0.40	F (9,17) = 0.42	F (9,17) = 0.32	F (9,17) = 0.25
N	5850	6053	8553	9167	6,375	6,777

Pearson test with jack-knife 95% confidence intervals for cell proportions, N= number of observations. *Not significant after correcting for multiple hypothesis testing with the sharpened Qs. Significance was set at p-value = 0.05. Goodness of fit test (Prob>). Ethiopia data set only had an urban variable. We classified an urban population size <50,000 as rural and >50,000 as urban. The regressions also controlled for the dummy variables province for Rwanda, Zambia, and Zimbabwe; region for Ethiopia; zone for Malawi; and mainlandzanziba for Tanzania. The results are not included. Q 1 – Lowest wealth quintile, Q 2, Q 3, Q 4, Q 5 – Highest wealth quintile.

For peer review only - http://bmjopen.bmj.com/site/about/guidelines.xhtml

Figure 2 shows the probabilities of self-reported cervical pre-cancer screening for selected combinations of age group (25–34, 35 – 49–years), residence (rural, urban), education level, and wealth quintile in percentage points by country. There were significant differences in cervical screening probabilities between the rural residents in the lowest wealth quintile with no education aged 25 – 34 years (25–34#Rural#Noed#1 in Figure 2) and their urban residents peers in the highest wealth quintile, highest educated (25–34#Urban#High#5) in Figure 2, ranging from 3.3% in Rwanda to 29.0% in Zambia. When similar comparisons were made for 35–49-year-olds, the differences ranged from 3.6% in Rwanda to 38.8% in Zimbabwe. A comparison of 25–34–years–old rural residents, with primary education in the second wealth quintile (25–34#Rural#Prim#2) and 35–49–year-old urban residents, with the highest education in the highest wealth quintile (35–49#Urban#High#5), showed substantially increased differences, ranging from 4.4% in Rwanda to 44.6% in Zimbabwe. Inequalities between 25–34–years–old urban residents, with the highest education, in the highest wealth quintile (25-34#Urban#High#5) and 35–49–year–old urban residents, with the highest education, in the highest wealth quintile (35–49#Urban#High#5) were moderate in magnitude, except in Rwanda where they were low (Figure 2).

[Figure 2 here]

Discussion

This study examined cervical pre-cancer screening inequalities related to age, residence, education, and wealth in six sub-Saharan African countries – Ethiopia, Malawi, Rwanda, Tanzania, Zambia, and Zimbabwe. We found that self-reported cervical pre-cancer was low and inequitable. None of the countries reached a third of 70% WHO cervical pre-cancer screening target, eight years away from the 2030 deadline. Self-reported cervical pre-cancer screening rates ranged from 3.5% (95% CI 3.1%–4.0%) in Rwanda to 17.1% (95% CI

For peer review only - http://bmjopen.bmj.com/site/about/guidelines.xhtml

15.8%–18.5%) Zambia and Zimbabwe (17.4% (CI16.1%–18.8%). However, older women, residing in urban areas, who were more educated and in the highest wealth quintile reported a higher uptake of cervical pre-cancer screening than younger women, living in rural areas, who were uneducated and in the lowest wealth quintile. Age-related inequalities were the most common – observed in all six surveyed countries. Rural-urban, education, and wealth-related inequalities were each observed in four countries, ranging from low to moderate in magnitude. We also observed high inequalities from combinations of inequalities in age group, residence (rural, urban), education level, and wealth in a few countries. These results have policy implications for the rapid scale-up of equitable life-saving cervical pre-cancer screening.

In this study, self-reported cervical pre-cancer screening was lower than a third of the WHO target of screening 70% of eligible women for cervical pre-cancer by 2030, consistent with other studies conducted in the surveyed countries. Studies found that cervical pre-cancer screening uptake ranged from 11% in Tanzania to 26.5% in Malawi, despite differences in methodologies used to estimate cervical pre-cancer screening (9,10). Countries should urgently scale up cervical pre-cancer screening to meet the WHO cervical pre-cancer screening target, removing inequalities that prevent some women from accessing screening for cervical pre-cancer, and integrate cervical pre-cancer screening in maternal, HIV, sexual and reproductive health, and social protection programmes.

Evidence supports the finding that inequalities in these underserved cervical pre-cancer screening countries favoured women who were older, resided in urban areas, were more educated and in the highest wealth quintile than younger women, who lived in rural areas, were uneducated and in the lowest wealth quintile (9,17,18). Studies conducted in Ethiopia, Rwanda, Tanzania, Zambia, and Zimbabwe, also support this finding (10,28,29,12,30,11,12,13,14,15). A study of 18 resource-constrained countries, of which

For peer review only - http://bmjopen.bmj.com/site/about/guidelines.xhtml

eight were from sub-Saharan Africa further found that wealth status increased socioeconomic inequalities in cervical pre-cancer screening, whereas being married, unemployed and living in urban communities reduced it (9). The results of this study agree with existing evidence on socioeconomic inequalities in maternal health. A study in Malawi found that rural-urban residence, education, and wealth status significantly impacted the uptake of three types of maternal health services in the study population. It found that compared with women residing in rural areas, who had lower education and were in the poorest wealth quintile, women living in urban areas, who had higher education and were in the higher wealth quintiles had significantly higher odds of receiving four antenatal care visits, skilled birth attendance and postnatal care (31). A study from Zambia reported similar findings (32). So did a study from 36 sub-Saharan African countries, concluding that prioritizing quality education of women in sub-Saharan Africa would reduce disparities in antenatal service utilization (33). Countries should urgently scale-up cervical pre-cancer screening, addressing inequalities that prevent some women from accessing services.

This study also determined the magnitude of the socioeconomic inequalities in reported cervical pre-cancer screening to aid policymaking in the prioritization of inequalities to eliminate in the scale-up of cervical pre-cancer screening. We found low to moderate age, rural-urban, education, and wealth-related inequalities in cervical pre-cancer screening in the surveyed countries. Age-related inequalities were reported in all the surveyed countries and residence, education, and wealth-related inequalities in four of the six surveyed countries. Although the inequalities were low to moderate, our study found that their combined effects on cervical pre-cancer screening probabilities were substantial, especially in countries with higher rates of self-reported cervical pre-cancer screening. The inequalities in cervical pre-cancer screening rates between 25 – 34-year-old rural residents, with no education in the lowest wealth quintile, and their urban resident peers, with highest education in the highest

For peer review only

wealth quintile, ranged from low inequalities in Rwanda, to moderate inequalities in Ethiopia and Tanzania to high inequalities in Malawi, Zambia, and Zimbabwe. Comparing primary educated 25–34–years–old rural residents, in the lowest wealth quintile with the highest educated 35–49–year–old urban residents, in the highest wealth quintile showed substantial differences. Inequalities did not disappear between the 25–34–years–old most affluent, highest educated, urban residents, and 35–49–year–old, most affluent, highest educated, urban residents. These results suggest that inequalities persist. Minor or moderate inequalities compound into high inequalities, further leaving women behind in cervical pre-cancer screening. Starting with age-related inequalities, eliminating socioeconomic inequalities should be prioritized in the urgent scale-up of cervical pre-cancer screening interventions.

Comparing the proportion of HIV–positive women in the sample and their reported cervical pre-cancer screening showed cervical pre-cancer screening gaps among HIV–positive women. In our study, the proportion of HIV positive 25–49-year-olds ranged from 4.7% (95% CI 4.1%–5.4%) 4.7 in Rwanda to 22.6% (95% CI 21.6%–23.7%) in Zimbabwe. By contrast, self-reported cervical pre-cancer screening among this population ranged from 0.4% (95% CI 0.3%–0.5%) in Rwanda to 5.4% (95% CI 4.7%–6.1%) in Zambia. Not all HIV–positive women were screened for cervical pre-cancer for several reasons. One reason may be the perception that linking women testing HIV positive to HIV treatment eliminates the risk of cervical pre-cancer for such women. A study in Zimbabwe found that many women living with HIV on ART screened positive for cervical pre-cancer, despite the country attaining HIV treatment targets (34). Another reason women may have not been screened for cervical pre-cancer is that they may have tested HIV positive at a facility with a limited capacity to conduct cervical pre-cancer screening. Alternatively, women might have been unable to access health facilities with the capacity to test for cervical pre-cancer for various reasons, including transport costs. A large proportion of HIV-negative women who were tested for

For peer review only - http://bmjopen.bmj.com/site/about/guidelines.xhtml

For peer review only

HIV in this study were not screened for cervical pre-cancer. One reason is the perception that HIV–negative women may be at low risk of cervical pre-cancer (14). Integration between HIV testing and cervical pre-cancer screening is required and is feasible in the scale up of cervical pre-cancer screening (35). Increased resources for HIV testing, training, and orientation of health care staff to screen eligible women for cervical pre-cancer, including HIV–negative women, is required to scale up screening services and reduce inequalities in the uptake of services.

Besides intensifying cervical pre-cancer screening in HIV programmes, governments should fulfil their obligations to the right to sexual and reproductive health by increasing the accessibility of their health systems (8). They should re-orient the building blocks of health systems – governance, financing, human resources, information, medical technologies, and service delivery– towards addressing inequalities and reaching all eligible women (8,36). They should establish, fund and scale-up population-level public-sector screening and treatment services and integrate them into HIV, STI, maternal and reproductive health services (37). Governments could build cervical pre-cancer screening around a target population, such as eligible schoolgirls and colleague students, which would assist in addressing socioeconomic inequalities. For example, Rwanda vaccinated more than 90% of schoolgirls against HPV from 2011 to 2012, through designated "health days" which were adapted for other sexual reproductive and maternal health purposes (38,39). This approach reduced time, money, and other costs of accessing HPV vaccination for girls, and other maternal health services for women (38). In contrast to other countries surveyed in this study, none of the wealth levels were associated with cervical pre-cancer screening in Rwanda. However, the cervical pre-cancer screening rate in Rwanda in this study was low at 3.5% (3.1%–4.0%), illustrating that cervical pre-cancer screening did not scale-up as did the HPV vaccination (39). Additional activities may be added to such programmes to increase access

For peer review only

and reduce inequalities. These include multiple screening over the lifetime, reducing the perception of high economic costs associated with cervical pre-cancer screening, and instituting same-day cervical pre-cancer screening and treatment (40). Other recommendations are the removal of economic barriers and focused outreach to women less likely to be screened and allowing them to self-collect samples (40,1,15). Women's ability to benefit from these programmes may depend on their social health protection, such as whether they have childcare responsibilities that prevent them from accessing services (21). Women with multiple inequalities depend on social protection and may be vulnerable under systems with low social protection (21). Governments should expand their social protection coverage and enhance their health system accessibility to scale up cervical pre-cancer screening, addressing the socioeconomic inequalities that prevent women from accessing services (21).

This study has several limitations. Collinearity was present, especially between the wealth and residence variables which tend to correlate with each other, consistent with how the wealth variable is constructed. Recall bias was present as cervical pre-cancer screening is self-reported. We did not control for the type of cervical pre-cancer screening because we did not have information on the types of screening women received. Cervical pre-cancer screening uptake may have worsened because of the COVID-19 pandemic. We did not determine if women had received two screenings by age 45 years as recommended by WHO, which could change our results. However, we used high-quality population-based data providing useful study findings to countries in sub-Saharan Africa.

Cervical pre-cancer screening in the surveyed countries was inequitable and low – less than a third of the WHO target to screen 70% of eligible women by 2030 for cervical pre-cancerous lesions. Women who were younger, lived in rural areas, had no education and were from poor backgrounds were left behind in cervical pre-cancer screening. Age-related inequalities were the most common. In combination, inequalities related to age, residence, education, and

For peer review only - http://bmjopen.bmj.com/site/about/guidelines.xhtml

wealth left younger, rural, uneducated, and poor women behind further. Focused and inclusive national cervical pre-cancer interventions are required to scale-up equitable cervical pre-cancer screening. Governments should include and monitor equity in their cervical pre-cancer screening programmes and research is needed to understand how to scale-up equitable cervical pre-cancer screening in sub-Saharan Africa countries.

a. Contributership statement

DC designed the study. DC and SA collated the data. DC, SA, SK, ALNM, MP, JB, JE, and OK performed the data analysis. DC conducted the data visualisation, and drafted, and revised the manuscript. SA, SK, ALNM, MP, JB, JE, and OK critically reviewed the drafts. All authors approved the final manuscript.

b. Competing interests

The authors declare no competing interests.

c. Funding statement

This grant was supported by Swiss National Science Foundation (SNSF) grant number 202660.

d. Data sharing statement

Detailed descriptions of the PHIA are https://phia-data.icap.columbia.edu/files.

e. Ethics declarations

The study did not need ethical clearance because the data is de-identified and publicly available at https://phia-data.icap.columbia.edu/files.

For peer review only - http://bmjopen.bmj.com/site/about/guidelines.xhtml

References

1. World Health Organization. Global strategy to accelerate the elimination of cervical cancer as a public health problem. Geneva: World Health Organization; 2020. Report No.: ISBN 978-92-4-001410-7.

2. World Health Organization. WHO guideline for screening and treatment of cervical pre-cancer lesions for cervical cancer prevention, second edition. Geneva: World Health Organization; 2021. Report No.: https://apps.who.int/iris/handle/10665/336583.

3. Rohner E, Bütikofer L, Schmidlin K, Sengayi M, Maskew M, Giddy J, et al. Cervical cancer risk in women living with HIV across four continents: A multicohort study. Int J Cancer. 2020 Feb 1; 146(3; doi: 10.1002/ijc.32260): 601-609.

4. Campos NG, Sharma M, Clark A, Lee K, Geng F, Regan C, et al. The health and economic impact of scaling cervical cancer prevention in 50 low- and lower-middle-income countries. Int J Gynaecol Obstet. 2017 Jul; Suppl 1(138 doi: 10.1002/ijgo.12184): 47-56.

5. World health Organizaation (WHO). Health Equity. [Online].; 2021 [cited 2021 November 10. Available from: https://www.who.int/health-topics/health-equity#tab=tab_1.

6. Emadi M, Delavari S, Bayati M. Global socioeconomic inequality in the burden of communicable and non-communicable diseases and injuries: an analysis on global burden of disease study 2019. BMC Public Health. 2021 September; 21(https://doi.org/10.1186/s12889-021-11793-7): 1771.

7. O'Donnell O, Doorslaer Ev, Wagstaff A, Lindelow M. Analyzing health equity using Household Survey Data. A guide to techniques and their Implementation Washington DC: The International Bank for Reconstruction and Development / The World Bank; 2008.

8. Perehudoff K, Vermandere H, Williams A, Bautista-Arredondo S, Paepe ED, Dias S, et al. Universal cervical cancer control through a right to health lens: refocusing national policy and programmes on underserved women. BMC Int Health Hum Rights. 2020 July; 20(21. doi.org/10.1186/s12914-020-00237-9).

9. Mahumud RA, Keramat SA, Ormsby GM, Sultana M, Rawal LB, Alam K, et al. Wealth-related inequalities of women's knowledge of cervical cancer screening and service utilisation in 18 resource-constrained countries: evidence from a pooled decomposition analysis. International Journal for Equity in Health. 2020 March; 19(42 doi.org/10.1186/s12939-020-01159-7).

10. Ayenew AA, Zewdu BF, Nigussie AA. Uptake of cervical cancer screening service and associated factors among age-eligible women in Ethiopia: systematic review and meta-analysis. Infectious Agents and Cancer. 2020 November; 15(doi.org/10.1186/s13027-020-00334-3): 67.

For peer review only

For peer review only - http://bmjopen.bmj.com/site/about/guidelines.xhtml

11. Henke A, Kluge U, Borde T, Mchome B, Serventi F, Henke O. Tanzanian women´s knowledge about Cervical Cancer and HPV and their prevalence of positive VIA cervical screening results. Data from a Prevention and Awareness Campaign in Northern Tanzania, 2017 – 2019. Glob Health Action. 2021 Jan; 14(1 doi: 10.1080/16549716.2020.1852780): 1852780.

12. Weng Q, Jiang J, Haji FM, Nondo LH, Zhou H. Women's knowledge of and attitudes toward cervical cancer and cervical cancer screening in Zanzibar, Tanzania: a cross-sectional study. BMC Cancer. 2020 January; 20(doi.org/10.1186/s12885-020-6528-x): 63.

13. Barrow A, Onikan A, Nzoputam CI, Ekholuenetale M. Prevalence and determinants of cervical cancer awareness among women of reproductive age: evidence from Benin and Zimbabwe population-based data. Appl Cancer Res. 2020 October; 40(doi.org/10.1186/s41241-020-00092-z).

14. Moucheraud C, Kawale P, Kafwafwa S, Bastani R, Hoffma RM. "It is big because it's ruining the lives of many people in Malawi": Women'sattitudes and beliefs about cervical cancer. Preventive Medicine Reports. 2020 June; 18(DOI: 10.1016/j.pmedr.2020.101093): 101093.

15. Pry JM, Manasyan A, Kapambwe S, Taghavi K, Duran-Frigola M, Mwanahamuntu M, et al. Cervical cancer screening outcomes in Zambia, 2010–19: a cohort study. The Lancet Global Health. 2021 June; 9(6 DOI:https://doi.org/10.1016/S2214-109X(21)00062-0): E832-E840.

16. Nyambe A, Kampen JK, Baboo SK, Hal GV. Knowledge, attitudes and practices of cervical cancer prevention among Zambian women and men. BMC Public Health 19, 508 (2019). https://doi.org/10.1186/s12889-019-6874-2. BMC Public Health. 2019 May; 19(508 doi.org/10.1186/s12889-019-6874-2).

17. Akanda R, Kawale P, Corrina M. Cervical cancer prevention in Africa: A policy analysis. Journal of Cancer Policy. 2022 January;(https://doi.org/10.1016/j.jcpo.2021.100321).

18. Akokuwebe ME, Idemudia ES, Lekulo AM, Motlogeloa OW. Determinants and levels of cervical Cancer screening uptake among women of reproductive age in South Africa: evidence from South Africa Demographic and health survey data, 2016. BMC Public Health. 2021 November; 21(1, doi.org/10.1186/s12889-021-12020-z): 2013.

19. Keetile M, Ndlovu K, Letamo G, Disang M, Yaya S, Navaneetham K. Factors associated with and socioeconomic inequalities in breast and cervical cancer screening among women aged 15–64 years in Botswana. PLoS ONE. 2021 August; 16(8 doi.org/10.1371/journal.pone.0255581): e0255581.

20. Quizhpe E, Sebastian MS, Teran E, Pulkki-Brännström AM. Socioeconomic inequalities in women's access to health care: has Ecuadorian health reform been successful? International Journal for Equity in Health. 2020 October; 19(doi.org/10.1186/s12939-020-01294-1): 178.

21. Prez VD, Jolidon V, Willems B, Cullati S, Burton-Jeangros C, Bracke P. Cervical cancer screening programs and their context-dependent effect on inequalities in screening uptake: a dynamic interplay between public health policy and welfare state redistribution. Int J Equity Health. 20 September; 20(doi.org/10.1186/s12939-021-01548-6): 211.

For peer review only - http://bmjopen.bmj.com/site/about/guidelines.xhtml

22. Jolidon V, Prez VD, Willems B, Bracke P, Cullati S, Burton-Jeangros C. Never and under cervical cancer screening in Switzerland and Belgium: trends and inequalities. BMC Public Health. 2020 October; 20(doi.org/10.1186/s12889-020-09619-z): 1517.

23. Stelzle D, Tanaka LF, Lee KK, Khalil AI, Baussano I, Shah ASV, et al. Estimates of the global burden of cervical cancer associated with HIV. Lancet Glob Health. 2021 February; 9(2. doi.org/10.1016/S2214-109X(20)30459-9): E161-E169.

24. World Health Organization. State of inequality: HIV, tuberculosis and malaria. Geneva: World Health Organization; 2021. Report No.: Licence: CC BY-NC-SA 3.0 IG.

25. Population-Based HIV Impact Assessment (PHIA) Data Use Manual. New York:; July 2019.

26. Population-Based HIV Impact Assessments (PHIA) Project. Population-Based HIV Impact Assessments (PHIA) Guiding the Global AIDS Response. [Online].; 2021 [cited 2021 February 22. Available from: https://phia-data.icap.columbia.edu/files.

27. Ogrinc G, Davies L, Goodman D, Batalden P, Davidoff F, Stevens D. SQUIRE 2.0 (Standards for QUality Improvement Reporting Excellence): revised publication guideliness from a detailed consensus process. BMJ Qual Saf. 2015.. 2015;(PMID: 26369893).

28. Niyonsenga G, Gishoma D, Sego R, Uwayezu MG, Nikuze B, Fitch M, et al. Knowledge, utilization and barriers of cervical cancer screening among women attending selected district hospitals in Kigali - Rwanda. Canadian Oncology Nursing Journal. 2021 July; 31(3. doi: 10.5737/23688076313266274): 266–274.

29. Ndateba I, Kabatsinda A, Ndabarora E. Uptake of Cervical Cancer Screening and Associated Factors Among Women Attending Outpatient Services in Rwamagana Hospital, Rwanda. Rwanda Journal of Medicine and Health Sciences. 2021 December; 4(3. doi: 10.4314/rjmhs.v4i3.8).

30. Kapambwe S, Sahasrabuddhe VV, Blevins M, Mwanahamuntu MH, Mudenda V, Shepherd BE, et al. Implementation and Operational Research: Age Distribution and Determinants of Invasive Cervical Cancer in a "Screen-and-Treat" Program Integrated With HIV/AIDS Care in Zambia. J Acquir Immune Defic Synd. 2015 Sep; 70(1. doi: 10.1097/QAI.0000000000000685): e20-6..

31. Yaya S, Bishwajit G, Shah V. Wealth, education and urban–rural and maternal healthcare service usage in Malawi. BMJ Global Health. 2016; 1(doi:10.1136/bmjgh-2016-000085): e000085.

32. He Z, Zhang C, Wang S, Yang X. Socioeconomic Determinants of Maternal HealthCare Utilisation in Zambia: 1997-2014. INQUIRY: The Journal of Health Care Organization, Provision, and Financing. 2021; S8(doi: 10.1177/00469580211067480): 1-14.

33. Obse AG, Ataguba JE. Explaining socioeconomic disparities and gaps in the use of antenatal care services in 36 countries in sub-Saharan Africa. Health Policy and Planning. 2021 June ; 36(5): 651–661.

34. Taghavi K, Mandiriri A, Shamu T, Rohner E, Bütikofer L, Asangbeh S, et al. Cervical Cancer Screening Cascade for women living with HIV: A cohort study from Zimbabwe. PLOS Glob Public Health. 2022 Feb; 2(2. https://doi.org/10.1371/journal.pgph.0000156): e0000156.

For peer review only - http://bmjopen.bmj.com/site/about/guidelines.xhtml

35. Pfaff C, Singano V, Akello H, Amberbir A, Berman J, Kwekwesa A, et al. Early experiences in integrating cervical cancer screening and treatment into HIV services in Zomba Central Hospital, Malawi. Malawi Med J. 2018 Sep; 30(3: doi: 10.4314/mmj.v30i3.14.): 211-214.

36. Gravitt PE, Silver MI, Hussey HM, Arrossi S, Huchko M, Jeronimo J, et al. Achieving equity in cervical cancer screening in low- and middle-income countries (LMICs): Strengthening health systems using a systems thinking approach. Preventive Medicine. 2021 March; 44(https://doi.org/10.1016/j.ypmed.2020.106322): 106322.

37. Parham GP, MwanahamuntuV MH, Kapambwe S, Muwonge R, Bateman AC, Blevins M, et al. Population-level scale-up of cervical cancer prevention services in a low-resource setting: Development, implementation, and evaluation of the cervical cancer prevention program in Zambia. PLoS ONE. 2015; 10(4. doi.org/10.1371/journal.pone.0122169).

38. Binagwaho A, Ngabo F, Wagner CM, Gatera M, Nutt CT, Nsanzimana S. Lessons from the field. Bulletin of the World Health Organisation. 2013; 91(doi: http://dx.doi.org/10.2471/BLT.12.116087): 697–703.

39. Muhimpundu MA, Ngabo F, Sayinzoga F, Balinda JP, Rusine J, Harward S, et al. Screen, Notify, See, and Treat: Initial Results of Cervical Cancer Screening and Treatment in Rwanda. JCO Global Oncology. 2021; 7(DOI: 10.1200/GO.20.00147): 632-638.

40. Campos NG, VT, Jeronimo J, Mvundura M, Kim JJ. Evidence-based policy choices for efficient and equitable cervical cancer screening programs in low-resource settings. Cancer Medicine. 2017 May; 6(8. doi: 10.1002/cam4.1123): 2008–2014.

41. Joint United Nations Programme on HIV/AIDS. UNAIDS 2021 epidemiological estimates. Geneva: Joint United Nations Programme on HIV/AIDS; 2021. Report No.: www.aidsinfo.unaids.org.

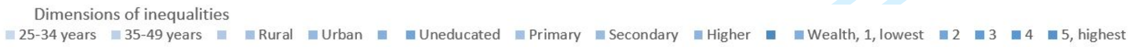

Figure 1. Survey weighted proportions of 25–49-year-olds reporting being screened for cervical pre-cancer by age-groups, rural versus urban residence, education, and wealth quintile by country (Percentage, 95% CI) (PHIA 2015–2018).

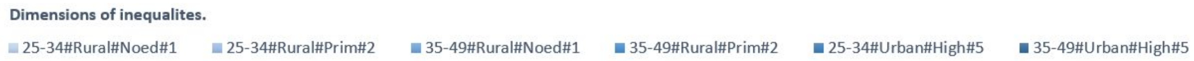

1=lowest wealth quintile, 2, 3, 4, 5= highest wealth quintile. 25-34# =25 – 34, 35-49#=35 – 49 years old. #Rural=rural. #Rich= rich. Noed = no education, Prim= primary, High=higher education.

For peer review only - http://bmjopen.bmj.com/site/about/guidelines.xhtml

Figure 2. Survey weighted differences in probabilities of self-reported cervical pre-cancer screening by combinations of age-group, rural-urban residence, no education, higher education, and lowest to highest wealth quintiles by country. Marginal effects (percentages, 95% CI) (PHIA 2015–2018).

For peer review only

Figure 1

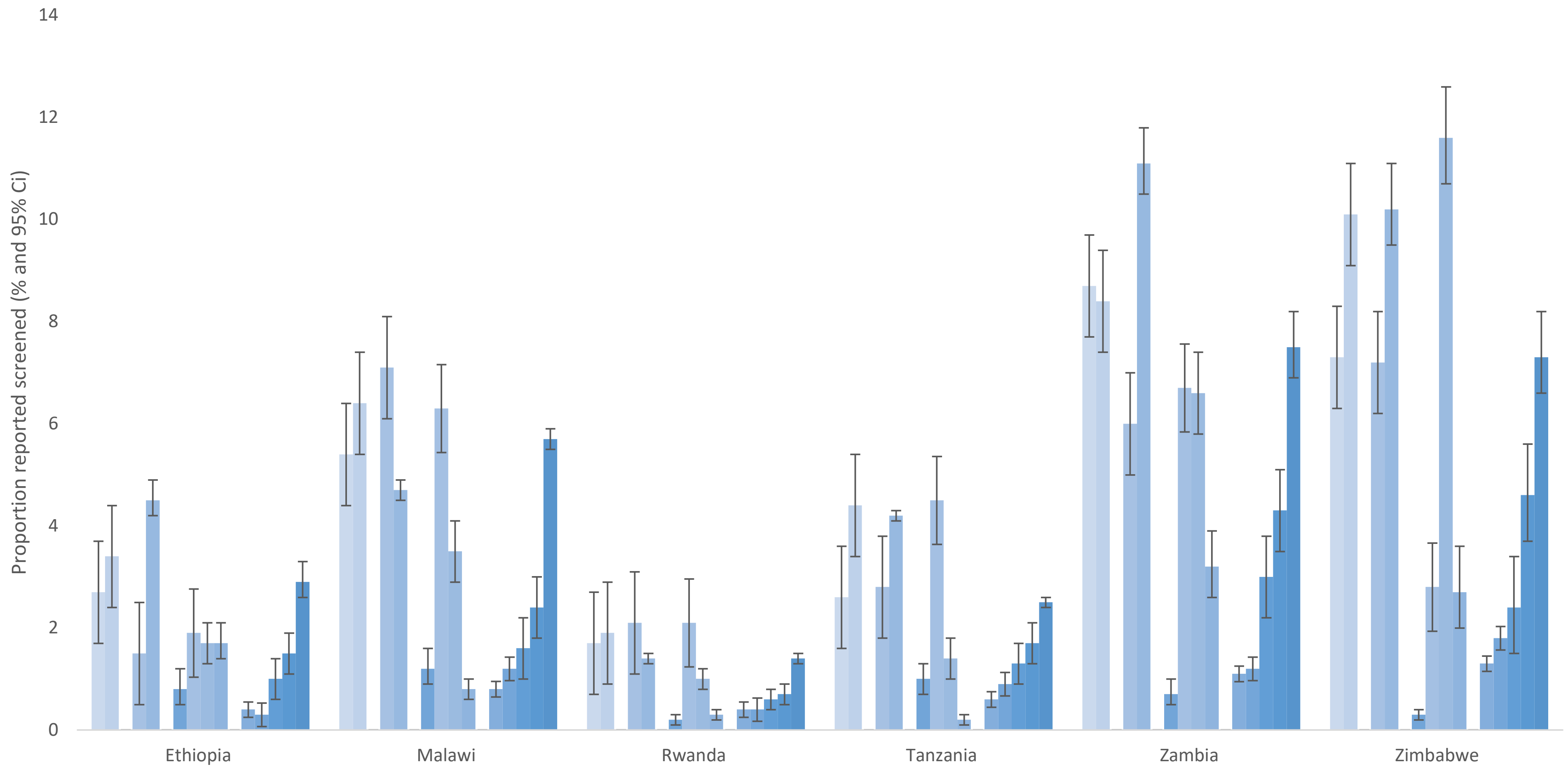
14
12
10
8
6
4
2
0
Proportion reported screened (% and 95% Ci)
Ethiopia
Malawi
Rwanda
Tanzania
Zambia
Zimbabwe

Figure 2

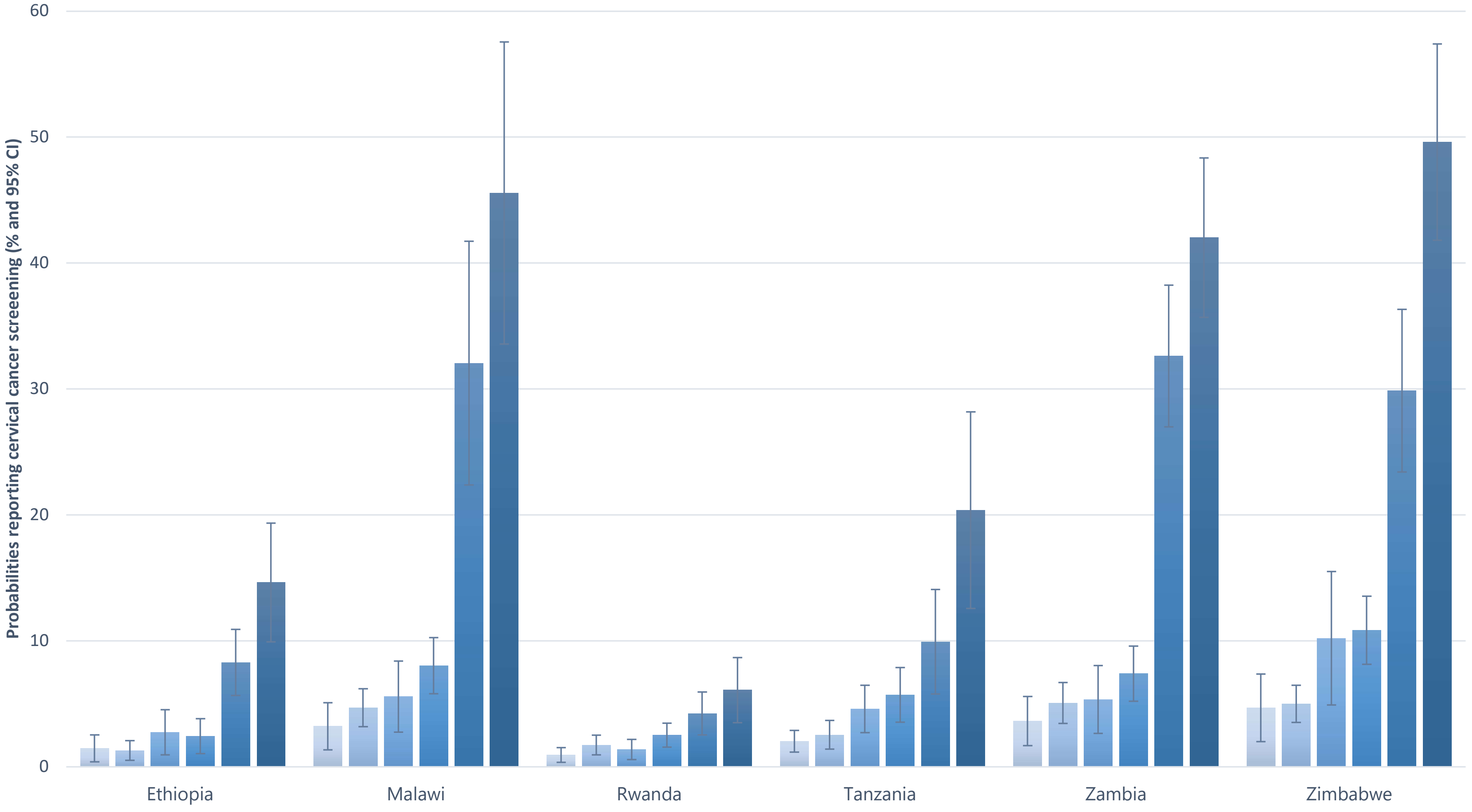
Probabilities reporting cervical cancer screeening (% and 95% CI)
0
10
20
30
40
50
60
Ethiopia
Malawi
Rwanda
Tanzania
Zambia
Zimbabwe

Supplementary Table 1. Variable descriptions.

Variable name	Description	Measure
Cervical pre-cancer screening	Self-reported from the adult interview question on ever testing for cervical pre-cancer, coded no, yes.	Binary
Sex	Self-reported sex (i.e., male, female).	Binary
Age	Self-reported linear years categorized in years (25–34, 35–49).	Categorical
Location, rural, urban	Self-reported residence (i.e., rural, urban), from the household interview. The data from Ethiopia had only urban variables. For Ethiopia, we coded size of area <=50,000 as rural = 0, and >50k =1; urban, from the household data set.	Binary
Ever married	Was derived from the question 'Have you ever been married or lived together with a (man/woman) as if married?' coded no, yes.	Binary
Ever tested for HIV	From the question 'Has been tested for HIV', coded never tested, ever tested.	Binary
HIV prevalence	The proportion of people 15 years or older testing HIV positive using laboratory-confirmed HIV test results from the adult biomarker questionnaire.	Binary
Educational status	Level of school respondent ever attended (i.e., not educated, primary school education, secondary school education, or higher education, from the adult interview. Tanzania's educations variables were standardized as follows: No education; Primary combined pre-primary, primary and post-primary; Secondary combined secondary, post-secondary training, secondary A); More than secondary (included post-secondary A levels and university).	Categorical
Wealth	Assessed household wealth with the wealth quintiles variable, which was derived from a wealth index. Wealth quintiles ranked households depending on their household characteristics and asset ownership from Q1 representing the poorest households to Q5 representing the wealthiest. (i.e., poorest, poor, rich, richer, and richest). The variable is provided in the PHIA household data set.	Ordered ranking
Regions, province, or zone	Defined as a political area, which is the primary sampling unit. It was province for Rwanda, Zambia, and Zimbabwe; region for Ethiopia, zone for Malawi, and mainlandzanziba for Tanzania.	Categorical

Note: Unless indicated the variables were from the adult interview data set. The answer options 'didn't know' or 'refused to answer' were coded as missing for all variables.

For peer review only

Reporting checklist for quality improvement in health care.

Based on the SQUIRE guidelines.

Instructions to authors

Complete this checklist by entering the page numbers from your manuscript where readers will find each of the items listed below.

Your article may not currently address all the items on the checklist. Please modify your text to include the missing information. If you are certain that an item does not apply, please write "n/a" and provide a short explanation.

Upload your completed checklist as an extra file when you submit to a journal.

In your methods section, say that you used the SQUIREreporting guidelines, and cite them as:

Ogrinc G, Davies L, Goodman D, Batalden P, Davidoff F, Stevens D. SQUIRE 2.0 (Standards for QUality Improvement Reporting Excellence): revised publication guidelines from a detailed consensus process

For peer review only

		Reporting Item	Page Number
**Title**			
	#1	Indicate that the manuscript concerns an initiative to improve healthcare (broadly defined to include the quality, safety,	1

		effectiveness, patientcenteredness, timeliness, cost, efficiency, and equity of healthcare)	
**Abstract**			
	#02a	Provide adequate information to aid in searching and indexing	2
	#02b	Summarize all key information from various sections of the text using the abstract format of the intended publication or a structured summary such as: background, local problem, methods, interventions, results, conclusions	2
**Introduction**			
Problem description	#3	Nature and significance of the local problem	3
Available knowledge	#4	Summary of what is currently known about the problem, including relevant previous studies	3
Rationale	#5	Informal or formal frameworks, models, concepts, and / or theories used to explain the problem, any reasons or assumptions that were used to develop the intervention(s), and reasons why the intervention(s) was expected to work	3
Specific aims	#6	Purpose of the project and of this report	4
**Methods**			
Context	#7	Contextual elements considered important at the outset of introducing the intervention(s)	5

For peer review only

Intervention(s)	#08a	Description of the intervention(s) in sufficient detail that others could reproduce it	N/A
Intervention(s)	#08b	Specifics of the team involved in the work	N/A
Study of the Intervention(s)	#09a	Approach chosen for assessing the impact of the intervention(s)	5
Study of the Intervention(s)	#09b	Approach used to establish whether the observed outcomes were due to the intervention(s)	6
Measures	#10a	Measures chosen for studying processes and outcomes of the intervention(s), including rationale for choosing them, their operational definitions, and their validity and reliability	6
Measures	#10b	Description of the approach to the ongoing assessment of contextual elements that contributed to the success, failure, efficiency, and cost	N/A
Measures	#10c	Methods employed for assessing completeness and accuracy of data	6
Analysis	#11a	Qualitative and quantitative methods used to draw inferences from the data	6
Analysis	#11b	Methods for understanding variation within the data, including the effects of time as a variable	N/A
Ethical considerations	#12	Ethical aspects of implementing and studying the intervention(s) and how they were addressed, including, but	20

For peer review only

		not limited to, formal ethics review and potential conflict(s) of interest	
**Results**			
	#13a	Initial steps of the intervention(s) and their evolution over time (e.g., time-line diagram, flow chart, or table), including modifications made to the intervention during the project	N/A
	#13b	Details of the process measures and outcome	6-9
	#13c	Contextual elements that interacted with the intervention(s)	6-9
	#13d	Observed associations between outcomes, interventions, and relevant contextual elements	6-9
	#13e	Unintended consequences such as unexpected benefits, problems, failures, or costs associated with the intervention(s).	N/A
	#13f	Details about missing data	6
**Discussion**			
Summary	#14a	Key findings, including relevance to the rationale and specific aims	9
Summary	#14b	Particular strengths of the project	14
Interpretation	#15a	Nature of the association between the intervention(s) and the outcomes	N/A
Interpretation	#15b	Comparison of results with findings from other publications	10-11

For peer review only

Interpretation	#15c	Impact of the project on people and systems	N/A
Interpretation	#15d	Reasons for any differences between observed and anticipated outcomes, including the influence of context	12 - 14
Interpretation	#15e	Costs and strategic trade-offs, including opportunity costs	N/A
Limitations	#16a	Limits to the generalizability of the work	14
Limitations	#16b	Factors that might have limited internal validity such as confounding, bias, or imprecision in the design, methods, measurement, or analysis	14
Limitations	#16c	Efforts made to minimize and adjust for limitations	14
Conclusion	#17a	Usefulness of the work	14
Conclusion	#17b	Sustainability	N/A
Conclusion	#17c	Potential for spread to other contexts	14
Conclusion	#17d	Implications for practice and for further study in the field	14
Conclusion	#17e	Suggested next steps	14
**Other information**			
Funding	#18	Sources of funding that supported this work. Role, if any, of the funding organization in the design, implementation, interpretation, and reporting	2

For peer review only

None The SQUIRE 2.0 checklist is distributed under the terms of the Creative Commons Attribution License CC BY-NC 4.0. This checklist can be completed online using https://www.goodreports.org/, a tool made by the EQUATOR Network in collaboration with Penelope.ai

For peer review only

Socioeconomic inequalities in cervical pre-cancer screening among women in Ethiopia, Malawi, Rwanda, Tanzania, Zambia, and Zimbabwe – Analysis of Population-Based HIV Impact Assessment surveys.

Journal:	*BMJ Open*
Manuscript ID	bmjopen-2022-067948.R1
Article Type:	Original research
Date Submitted by the Author:	17-Nov-2022

Complete List of Authors:	Chipanta, David; University of Geneva, Faculty of Medicine; UNAIDS, ERA<br>Kapambwe, Sharon; World Health Organisation<br>Nyondo-Mipando, Alinane Linda; Kamuzu University of Health Sciences<br>Pascoe, Margaret; Newlands Clinic, Clinic<br>Amo-Agyei, Silas; University of Lausanne, Department of Economics<br>Bohlius, Julia; University of Bern, Swiss Tropical and Public Health Institute<br>Estill, Janne; University of Geneva<br>Keiser, Olivia ; University of Geneva

**Primary Subject Heading**:	Epidemiology
Secondary Subject Heading:	Health economics, Health policy, Health services research
Keywords:	Cancer pain < ONCOLOGY, HIV & AIDS < INFECTIOUS DISEASES, PUBLIC HEALTH, Health policy < HEALTH SERVICES ADMINISTRATION & MANAGEMENT, Health economics < HEALTH SERVICES ADMINISTRATION & MANAGEMENT, Reproductive medicine < GYNAECOLOGY

SCHOLARONE™
Manuscripts

I, the Submitting Author has the right to grant and does grant on behalf of all authors of the Work (as defined in the below author licence), an exclusive licence and/or a non-exclusive licence for contributions from authors who are: i) UK Crown employees; ii) where BMJ has agreed a CC-BY licence shall apply, and/or iii) in accordance with the terms applicable for US Federal Government officers or employees acting as part of their official duties; on a worldwide, perpetual, irrevocable, royalty-free basis to BMJ Publishing Group Ltd ("BMJ") its licensees and where the relevant Journal is co-owned by BMJ to the co-owners of the Journal, to publish the Work in this journal and any other BMJ products and to exploit all rights, as set out in our licence.

The Submitting Author accepts and understands that any supply made under these terms is made by BMJ to the Submitting Author unless you are acting as an employee on behalf of your employer or a postgraduate student of an affiliated institution which is paying any applicable article publishing charge ("APC") for Open Access articles. Where the Submitting Author wishes to make the Work available on an Open Access basis (and intends to pay the relevant APC), the terms of reuse of such Open Access shall be governed by a Creative Commons licence – details of these licences and which Creative Commons licence will apply to this Work are set out in our licence referred to above.

Other than as permitted in any relevant BMJ Author's Self Archiving Policies, I confirm this Work has not been accepted for publication elsewhere, is not being considered for publication elsewhere and does not duplicate material already published. I confirm all authors consent to publication of this Work and authorise the granting of this licence.

For peer review only

Socioeconomic inequalities in cervical pre-cancer screening among women in Ethiopia, Malawi, Rwanda, Tanzania, Zambia, and Zimbabwe – Analysis of Population-Based HIV Impact Assessment surveys

David Chipanta[1,2] *, Sharon Kapambwe[3], Alinane Linda Nyondo-Mipando[4], Margaret Pascoe[5], Silas Amo-Agyei[6], Julia Bohlius[7 8], Janne Estill[1], Olivia Keiser[1]

1 Institute of Global Health, University of Geneva, Geneva, Switzerland.
2 Joint United Nations Programme on HIV/AIDS (UNAIDS), Geneva, Switzerland.
3 World Health Organisation, Harare, Zimbabwe.
4 School of Global and Public Health, Kamuzu University of Health Sciences, Blantyre, Malawi.
5 Newlands Clinic, Harare, Zimbabwe.
6 Department of Economics, University of Lausanne, Lausanne, Switzerland.
7 Swiss Tropical and Public Health Institute, Allschwil, Switzerland.
8 University of Basel, Basel, Switzerland

*Corresponding author: Email: chipanta@hotmail.com; david.chipanta@etu.unige.ch

E-mail addresses of co-authors:
SK: kapambwes@who.int
ALNM- lmipando@kuhes.ac.mw
MP MargaretP@newlandsclinic.org.zw
SA: silas.amo-agyei@unil.ch
JB julia.bohlius@swisstph.ch
JE: Janne.Estill@unige.ch
OK: Olivia.Keiser@unige.ch

Where authors are identified as personnel of the International Agency for Research on Cancer / World Health Organization, or UNAIDS, the authors alone are responsible for the views expressed in this article and they do not necessarily represent the decisions, policy, or views of the International Agency for Research on Cancer / World Health Organization or UNAIDS.

For peer review only - http://bmjopen.bmj.com/site/about/guidelines.xhtml

Abstract

Background:
Screening for cervical pre-cancerous lesions may be inequitable among women in sub-Saharan African countries. This study examined socioeconomic inequalities in cervical pre-cancer screening among women in Ethiopia, Malawi, Rwanda, Tanzania, Zambia, and Zimbabwe.

Methods:
We used the available Population-Based HIV Impact Assessment (PHIA) datasets. We measured the inequality in screening rates between subgroups, considering absolute differences of ≥ 20 percentage points as high, < 20 but > 5, medium, ≤ 5 but > 0 percentage points as low. We described the sample characteristics for each country and examined the associations between screening rates and age groups, residence, education, and wealth levels. We analysed the differences using multivariable logistic regressions, controlling for covariates. Using marginal effects models, we estimated the inequalities in screening probabilities for each dimension and their combinations.

Results:
The sample size of women aged 25–49 comprised 5882 in Ethiopia, 6062 in Malawi, 8571 in Rwanda, 9186 in Tanzania, 6413 in Zambia and 6784 in Zimbabwe. The screening rates were low, ranging from 3.5% (95% Confidence Interval (CI) 3.1%–4.0%) in Rwanda to 17.4% (95% CI 16.1%–18.8%) in Zimbabwe. Inequalities in screening rates were low. Combining the dimensions led to significant inequalities in screening rates between subgroups. For example, the differences between women living in rural areas aged 25–34 years, primary educated, and least wealthy, and women living in urban areas aged 35–49 years, highest educated and most affluent, ranged from 4.4 to 44.6 percentage points in Rwanda and Zimbabwe.

Conclusions:
Cervical pre-cancer screening was low and inequitable. No surveyed country achieved one-third of the World Health Organisation target of screening 70% of eligible women for cervical pre-cancer by 2030. Inequalities in cervical pre-cancer screening were low. Their combinations led to high inequalities between sub-groups of women. Including indicators to monitor equity in cervical pre-cancer screening programmes is required.

Keywords: Socioeconomic inequalities, equity, cervical pre-cancer screening, age, rural-urban, education and wealth

Strengths and limitations

- The timelines and contexts for reaching the WHO target to screen 70% of eligible women for cervical pre-cancer by 2030 differ by country.
- We did not determine if women had received two screenings by age 45, as recommended by WHO, which could change our results. This information was unavailable.
- There was evidence of collinearity, especially between the wealth and rural urban residence variables which tend to correlate but was not problematic because of a low (<2) variance inflation factor (VIF).
- We did not control for the type of cervical pre-cancer screening because we did not have information on the types of screening the women received.
- However, we used high-quality, large, and standardized population-based data of multiple countries, enabling comparisons across countries.

For peer review only - http://bmjopen.bmj.com/site/about/guidelines.xhtml

Background

Although all countries are affected by cervical cancer, its incidence is more than twice, and mortality more than three times as high in low-and-middle-income countries as in high-income countries (1). In 2020, cervical cancer was the leading cause of cancer deaths in 36 countries. Most of these countries were in sub-Saharan Africa, Melanesia, South America, and South-Eastern Asia (2). Cervical cancer occurs in the lower part of the uterus that connects to the vagina. It is caused in over 70% of cases by the human papillomavirus (HPV), the most common sexually transmitted infection (STI) (1). Most HPV strains are harmless in people with a healthy immune system (1). HIV-positive women, whose immunity may be compromised (1,2), if co-infected with HPV, are six times more likely to develop cervical cancer than HIV negative women (1). In 2014, invasive cervical cancer was 11 times higher among women living with HIV in South Africa than in Europe (3). Cervical cancer harms girls, women and their families with significant health and development consequences, yet it is preventable and curable if detected and treated early (1,2). HPV vaccination and regular pre-cancer screening stop cervical pre-cancer from developing into cervical cancer. Cervical pre-cancer screening identifies pre-cancerous cells to treat before they become cancer (1). Comprehensive HPV vaccination and cervical pre-cancer screening could prevent an estimated 5.2 million cases and 3.7 million deaths over 10 years and would cost around US $3.2 billion in 50 low and lower-middle-income countries (4). In 2020, the World Health Organisation (WHO) proposed the 90–70–90 target, which, if achieved by 2030, would put the world on course to eliminate cervical cancer (1). The 90–70–90 target seeks to ensure that 90% of girls are vaccinated against HPV by age 15 years, 70% of eligible women are screened by age 35 years and again by 45, and 90% of women identified with cervical cancer receive treatment (1). Cervical pre-cancer screening is a critical component of the 90–70–90 target (5).

For peer review only - http://bmjopen.bmj.com/site/about/guidelines.xhtml

Socioeconomic inequalities prevent women from accessing cervical pre-cancer screening. Studies show that type of residence, geographical location, education, wealth, age, health insurance status, and the capacity of health systems influence access to cervical pre-cancer screening. (6,7,8,9,10,11,12,13,14,15). These studies showed that women who were older, resided in urban areas, and were more educated and wealthier were screened more often than younger, rural residing, uneducated, and poor women, with a few exceptions (6,16,17,18,19). However, these studies did not estimate the magnitude of inequalities and their combined impact on cervical pre-cancer screening. Such studies are needed to scale up cervical pre-cancer screening and support the prioritization of inequalities to eliminate in cervical -pre-cancer screening, especially in east and southern African countries, where HIV has worsened the cervical cancer burden (20). This study aimed to examine age, residence type, education, and wealth related inequalities and their combined impacts on cervical pre-cancer screening in Ethiopia, Malawi, Rwanda, Tanzania, Zambia, and Zimbabwe. We hypothesized that significant inequalities in cervical pre-cancer screening rates favoured women who were older, resided in urban areas, were more educated, and wealthier.

Methods

Data and sample

This was a cross-sectional study of a secondary data analysis of available Population-Based HIV Impact Assessment (PHIA) data for countries that included cervical pre-cancer screening variables. The countries and the years in which the PHIA surveys were conducted include Ethiopia (2017–2018), Malawi (2015–2016), Rwanda (2018), Tanzania (2016–2017), Zambia (2016) and Zimbabwe (2015–2016). The PHIA surveys collected a range of health and socio-demographic data to evaluate the impact of HIV programs in countries supported by United States President's Emergency Plan for AIDS Relief (PEPFAR). We combined the

For peer review only - http://bmjopen.bmj.com/site/about/guidelines.xhtml

Household, and Adult PHIA datasets for this study. In the PHIA surveys, a household questionnaire was administered to the head-of-household, who provided information on the household related to wealth, and other socio demographic characteristics. Then, an individual questionnaire was administered to eligible and consenting adults aged 15 years or older in the household. The individual interviews assessed a range of HIV-related variables, including cervical pre-cancer screening and HIV testing.

We restricted our analysis to women aged 25–49 years who responded to the questions on cervical pre-cancer. This is the group WHO recommends for cervical pre-cancer screening – aged 30–49 years for the general population and 25–49 years for women living with HIV – including all gender diverse people at risk of cervical cancer (1). Examining inequalities in a population not recommended to receive the intervention may be incorrect because the population may not be receiving the intervention.

Variables and outcome descriptions

Inequality in health and health care is defined as the unequal distribution of health goods, services and outcomes across income or other measures of wealth. In contrast, equity is the lack of avoidable differences in the distribution of health goods and services or outcomes due to people's socioeconomic status in society (21,22,23). We assessed inequalities related to age, residence type, education, and wealth in cervical pre-cancer screening according to women's self-reports. These dimensions of inequalities are familiar sources of disadvantage or discrimination (24). Our outcome variable was self-reported ever testing for cervical pre-cancer. The main predictors were age, rural-urban residence, education, and wealth. Other covariates were having ever married, ever been tested for HIV, and regions. Regions, provinces or zones were coded as dummy variables and included to capture the variation in cervical pre-cancer screening by geographical location. Ethiopia's PHIA survey was

For peer review only - http://bmjopen.bmj.com/site/about/guidelines.xhtml

conducted only in urban areas. We coded the urban variable for Ethiopia as rural if the population size of the area was smaller than 50,000 people, and urban otherwise, to standardize the presentation of the data. Therefore, the interpretation of Ethiopia's urbanity reflects the size of the population in the area, not necessarily urbanity. Supplementary Table 1 describes the variables. We used the absolute difference in percentage of women screened for cervical pre-cancer (in percentage points) to measure the magnitude of inequality between subgroups. We denoted an absolute difference of 20 percentage points or more between two subgroups as high inequality, less than 20 percentage points but greater than 5 percentage points as medium inequality and 5 percentage points of less but greater than 0, as low inequality, as used by the WHO (24).

Analysis

We assessed inequalities in self-reported cervical pre-cancer screening in three steps. For each country, we first presented sample characteristics descriptively. Second, we determined the associations between screening for cervical pre-cancer and each dimension of inequality, and analysed the differences using multivariable logistics regression, controlling for covariates. We corrected the p-values of the odds ratios for multiple hypothesis testing with sharpened q's. The sharpened q's have been found easier to use and more robust than other methods (25). In step 3, after each logistic regression we used the Stata margins post command (marginal effects models) to estimate the probabilities of reporting cervical pre-cancer screening for each subgroup alone and combined with relevant subgroups, holding the other subgroups constant at their mean values. We applied survey weights to account for complex survey design. Using jack-knife replicate weights, 95% confidence intervals (CI) were estimated (26). We did not impute the missing data since all variables had less than 2% of missing values. We used Stata version 14 for the analyses. The study did not require ethical clearance. The data are publicly available and de-identified (27).

For peer review only - http://bmjopen.bmj.com/site/about/guidelines.xhtml

Patient and Public Involvement

The respondents were not involved in the design and data analysis of this study. It was not possible to involve them since this study is an analysis of secondary data. The data collection abided by highest standard of ethical research, patient and public engagement as stipulated on the PHIA website (27).

Results

Table 1 presents the descriptive statistics of the respondents for each surveyed country. The sample size of women aged 25–49 years ranged from 5882 in Ethiopia to 9186 in Tanzania. The median ages and interquartile ranges were 32 years (28–39) in Ethiopia, 33 years (29–40) in Zambia and 34 years (29–40) in Malawi, Rwanda, and Tanzania. The rate of self-reported cervical pre-cancer screening was lowest in Rwanda (3.5% [95% CI 3.1%–4.0%]) and highest in Zambia (17.1% [95% CI 15.8%–18.5%]) and Zimbabwe (17.4% [95% CI 16.1%–18.8%]). The HIV prevalence among 25–49-year-old women ranged from 4.7% (95% CI 4.1%–5.4%) in Rwanda to 22.6% (95% CI 21.6%–23.7%) in Zimbabwe, and the rate of cervical pre-cancer screening among HIV-positive females ranged from 0.4% (95% CI 0.3%–0.5%) in Rwanda to 5.4% (95% CI 4.7%–6.1%) in Zambia. Women aged 25 to 34 years comprised half or more of the sample in surveyed countries. Eighty-six percent or more of the respondents reported ever having been married, and 86% or more reported ever having been tested for HIV. More than half of the respondents resided in rural areas, excluding Ethiopia, where all respondents resided in urban areas, 54% of them in areas with <50,000 residents. Less than 20% of the respondents were uneducated, and nearly 30% had attained a primary school education. (Table 1).

For peer review only - http://bmjopen.bmj.com/site/about/guidelines.xhtml

For peer review only

Table 1: Survey weighted sample characteristics by country (PHIA 2015–2018), (percentage [95% confidence interval] sample size)

	**Ethiopia** (n=5882)	**Malawi** (n=6062)	**Rwanda** (n=8571)	**Tanzania** (n=9186)	**Zambia** (n=6413)	**Zimbabwe** (n=6784)
**Cervical Cancer screened**	6.1 (5.3–6.9),343	11.8 (10.6–13.1),926	3.5 (3.1–4.0),353	7.0 (6.1–8.1),586	17.1 (15.8–18.5),1066	17.4 (16.1–18.8),1129
**HIV positive**	6.1 (5.3–7.0),362	18.8 (17.3–20.3),1152	4.7 (4.1–5.4),416	9.2 (8.4–10.0),897	21.2 (19.8–22.6),1263	22.6 (21.6–23.7),1602
[a]HIV positive cervical Pre-cancer screened	0.9 (0.7–1.45),52	3.4 (2.8–4.1), 249	0.4 (0.3–0.5),43‡	1.5 (1.1 –2.0),132	5.4 (4.7–6.1),318	4.5 (4.0–5.0),305
**Age** (years): Median [IQR] years	32 (28–39)	34 (29–40)	34 (29–40)	34 (29–40)	33 (29–40)	34 (29-40)
25–34	55.9 (55.7–56.2),3517	53.0 (52.9–53.1),3342	52.1 (52.1–52.2),4508	52.2 (52.2–52.3),4812	55.3 (55.2–55.4),3386	54.5 (54.4–54.6),3403
35–49	44.1 (43.8–44.3),2365	47.0 (46.9–47.1),2720	47.9 (47.8–47.9),4063	47.8 (47.7–47.8),4374	44.7 (44.6–44.8),3027	45.5 (45.4–45.6),3381
**Ever married**	89.7 (88.4–90.9),5228	96.6 (95.9–97.1),5794	86.0 (85.0–87.0),7320	92.6 (91.6–93.6),8565	91.7 (90.6–92.7),5901	94.0 (93.3–94.6),6373
**Ever tested for HIV**	86.4 (84.9–87.7),5018	94.8 (94.0–95.4),5756	95.7 (95.2–96.1),8194	89.2 (88.2–90.1),8206	92.3 (91.5–93.1),5878	94.8 (94.1–95.4),6375
**Location** Urban	54.0 (48.8–59.1),3215	19.0 (16.3–21.9),2289	20.2 (16.5–24.5),2159	39.4 (35.7–43.4),3299	45.9 (42.4–49.4),2867	38.9 (37.0–40.9),2317
**Education:** No education	22.1 (20.0–24.3),1307	14.5 (13.2–15.9),747	11.5 (10.7–12.4),953	17.8 (16.3–19.5),1816	8.5 (7.2–9.9),545	1.8 (1.4–2.3),135
Primary	38.7 (36.7–40.7),2210	66.2 (64.4–68.0),3672	68.1 (66.4–69.7),5666	65.8 (64.2–67.4),5931	51.8 (49.5–54.1),3329	27.6 (26.1–29.2),2044
Secondary	21.1 (19.6–22.7),1230	16.7 (15.3–18.3),1351	15.8 (14.7–16.9),1434	14.7 (13.5–15.9),1297	30.3 (28.4 - 32.3),1953	62.8 (61.1–64.4),4146
Higher	18.1 (16.3–20.0),1122	2.5 (2.1–3.0),288	4.6 (3.9–5.5),510	1.7 (1.3–2.1),137	9.4 (8.0 - 11.1),583	7.8 (6.5–9.2),455
**Wealth quintiles:**						
1, lowest	14.6 (12.5–17.0),910	17.3 (15.7–19.1),748	20.4 (18.3–22.7),1600	18.3 (16.1–20.8),1821	16.3 (14.6–18.1),1055	18.9 (17.3–20.6),1530
2	16.1 (14.5–17.8),972	18.5 (17.0–20.1),863	19.2 (17.5–21.0),1542	19.6 (18.0–21.4),1807	17.5 (16.1–19.0),1140	18.4 (17.2–19.6),1318
3	19.1 (17.6–20.6),1144	19.7 (18.3–21.2),957	19.5 (18.1–21.0),1580	20.0 (18.3–21.8),2063	19.7 (17.9–21.7),1258	17.5 (16.0–19.1),1180
4	23.1 (21.3–25.0),1356	20.7 (19.3–22.2),1207	20.2 (18.7–21.9),1662	20.5 (18.6–22.5),1798	22.2 (20–24.6),1424	21.4 (19.1–23.8),1282
5, highest	27.1 (24.5–29.9),1500	23.7 (21.6–25.9),2287	20.6 (18.1–23.3),2182	21.6 (19.4–24.0),1694	24.3 (21.7–27.1),1506	23.9 (21.4–26.5),1474

Self-reported cervical pre-cancer screening, proportions in percentages, Pearson's test with jack-knife 95% confidence intervals, N is number of observations. The urbanity variable in Ethiopia's dataset was defined as population size of area <50,000 (rural) and >50,000 (urban). [a]Proportion screened among HIV positive women.

For peer review only

For peer review only - http://bmjopen.bmj.com/site/about/guidelines.xhtml

Figure 1 shows the proportion of respondents who reported being screened for cervical pre-cancer by age group (25–34 versus 35 – 49-years), residence (rural versus urban), education level, and wealth quintile in each country. Table 2 presents adjusted odds ratios. In all countries except Zambia, 35–49-year-old women reported screening for cervical pre-cancer more frequently than 25–34-year-old women (Figure 1). Age-related differences in reported screening varied and ranged from 0.2 percentage points (1.7% among 25–34-year-old women versus 1.9% among 35 – 49-year-old women) in Rwanda to 2.8% (7.3% among 25–34-year-old women versus 10.1% among 35 – 49-year-old women) in Zimbabwe. After adjusting for covariates, 35–49-year-old women in all countries surveyed reported being more likely to be screened for cervical pre-cancer than 25–34-year-old women (Table 2).

Urban residents reported screening for cervical pre-cancer more often than rural residents in Tanzania, Zambia, and Zimbabwe before adjusting for covariates. In Ethiopia, women in areas with more than 50,000 people reported screening for cervical pre-cancer more often than their counterparts living in areas with fewer than 50,000 people (Figure 1). The rural-urban inequalities in cervical pre-cancer screening were low, in Tanzania and Zimbabwe, and moderate in Zambia. After adjustment, urban residents in Malawi, Rwanda, and Tanzania reported a higher screening rate for cervical pre-cancer than rural residents (Table 2).

Education-related inequalities were low in Malawi, Rwanda, Zambia, and Zimbabwe, except Zambia, where it was moderate between the uneducated and those with secondary schooling (5.9 percentage points) (Figure 1). In Malawi and Tanzania, the inequalities between women in the lowest and highest wealth quintiles were low – 4.9 percentage points and 1.9 percentage points, respectively. They were moderate in Zambia (5.7 percentage points) and Zimbabwe (5.3 percentage points) (Figure 1). The goodness-of-fit test showed that our models fit the data well (Table 2).

For peer review only - http://bmjopen.bmj.com/site/about/guidelines.xhtml

Table 2: Survey weighted differences in self-reported cervical pre-cancer screening among 25 – 49-year-old women by age-groups, rural-urban location, education, and wealth quintile by country – (adjusted odds ratios [95% CI]) (PHIA 2015–2018)

**Inequality dimensions**	**Ethiopia** (n=5850)	**Malawi** (n=6053)	**Rwanda** (n = 8553)	**Tanzania** (n = 9167)	**Zambia** (n = 6,375)	**Zimbabwe** (n = 6,777)
**Age** (years) 35–49 (ref 25–34)	1.90 (1.48–2.43)	1.77 (1.39–2.27)	1.46 (1.11–1.93)	2.32 (1.85–2.91)	1.50 (1.28–1.75)	2.31 (1.92–2.78)
**Residence** Urban (ref: rural)	1.65 (1.02–2.65)	1.91 (1.29 - 2.82)	1.67 (1.05–2.65)	1.57 (1.03–2.41)	1.27 (0.96–1.68)	0.90 (0.65–1.24)
**Education** Primary (ref: no education)	0.97 (0.60 –1.54)	1.04 (0.74–1.48)	1.84 (1.04–3.26)	0.91 (0.61–1.36)	1.45 (0.97–2.16)	0.78 (0.44–1.38)
Secondary	1.32 (0.77–2.29)	1.69 (1.11–2.58)	3.11 (1.69–5.71)	1.19 (0.70–2.00)	1.81 (1.19–2.75)	1.10 (0.63–1.94)
Higher	1.73 (1.02–2.93)	2.10 (1.28–3.44)	2.08 (0.99–4.38)	1.38 (0.71–2.67)	2.52 (1.61–3.94)	1.95 (1.05–3.60)
**Wealth quintiles**: Q2 (ref: lowest Q1)	0.92 (0.46–1.85)	1.42 (0.85–2.36)	1.00 (0.60–1.66)	1.39 (0.78–2.49)	0.98 (0.61–1.58)	1.38 (1.00–1.91)
Q3	1.63 (0.75–3.51)	1.57 (0.97–2.55)	1.32(0.81–2.15)	1.72 (1.07–2.77)	2.24 (1.51–3.34)	2.04 (1.47–2.85)
Q4	1.78 (0.80–3.97)	2.16 (1.32–3.53)	1.25 (0.77–2.04)	1.93 (1.08–3.44)	2.40 (1.54–3.74)	3.24 (2.19–4.82)
Q5 Highest Quintile	2.13 (0.96–4.75)	3.53 (2.15 5.80)	1.34 (0.74–2.43)	2.44 (1.31– 4.54)7	4.01(2.51– 6.40)	4.95 (3.17–7.70)
**Goodness of fit** Prob > F	0.4317	0.9611	0.9176	0.9082	0.9569	0.9791
	F (9,17) = 1.07	F (9,17) = 0.31	F (9,17) = 0.40	F (9,17) = 0.42	F (9,17) = 0.32	F (9,17) = 0.25

Pearson test with jack-knife 95% confidence intervals for cell proportions, N= number of observations. *Not significant after correcting for multiple hypothesis testing with the sharpened Qs. Significance was set at p-value = 0.05. Goodness of fit test (Prob>). The urbanity variable in Ethiopia's dataset was defined as population size of area <50,000 (rural) and >50,000 (urban). The analysis was adjusted for all variables in the table as well as the geographical area (province for Rwanda, Zambia, and Zimbabwe; region for Ethiopia; zone for Malawi; and mainland versus Zanziba for Tanzania). Q 1 – Lowest wealth quintile, Q 2, Q 3, Q 4, Q 5 – Highest wealth quintile.

For peer review only

(Figure 1 here)

Figure 2 shows the probabilities of self-reported cervical pre-cancer screening for selected combinations of age groups (25–34, 35 – 49–years), residence (rural, urban), education levels, and wealth quintiles in percentage points by country. There were significant differences in cervical pre-cancer screening probabilities between the rural residents in the lowest wealth quintile with no education aged 25 – 34 years (25–34#Rural#Noed#1) and their peers residing in urban areas in the highest wealth quintile, and highest education level (25–34#Urban#High#5), ranging from 3.3% in Rwanda to 29.0% in Zambia (Figure 2). When similar comparisons were made for 35–49-year-olds, the differences ranged from 3.6 percentage points in Rwanda to 38.8 percentage points in Zimbabwe. A comparison of 25–34–year–old women residing in rural areas, with primary education in the second wealth quintile (25–34#Rural#Prim#2) and 35–49–year-old women residing in urban areas, with the highest education, and in the highest wealth quintile (35–49#Urban#High#5), showed substantial differences. The differences ranged from 4.4 percentage points in Rwanda to 44.6 percentage points in Zimbabwe. Inequalities between 25–34–years–old urban residents, with the highest education, in the highest wealth quintile (25-34#Urban#High#5) and 35–49–year–old urban residents, with the highest education, in the highest wealth quintile (35–49#Urban#High#5) were moderate in magnitude, except in Rwanda where they were low (Figure 2).

(Figure 2 Here).

For peer review only - http://bmjopen.bmj.com/site/about/guidelines.xhtml

Discussion

This study examined socioeconomic inequalities in cervical pre-cancer screening related to age, residence, education, and wealth in six sub-Saharan African countries – Ethiopia, Malawi, Rwanda, Tanzania, Zambia, and Zimbabwe. We found that self-reported cervical pre-cancer screening was low, ranging from 3.5% in Rwanda to 17.4% in Zimbabwe. It was inequitable, with women who were older, residing in urban areas, more educated, and in the highest wealth quintile, reporting a higher uptake of cervical pre-cancer screening than women who were younger, living in rural areas, uneducated, and in the lowest wealth quintile. Age-related inequalities were common and observed in all six surveyed countries. Inequalities related to rural-urban residence, education, and wealth were each observed in four countries, ranging from low to moderate in magnitude. High inequalities were observed between combinations of age group, residence (rural, urban), education levels, and wealth levels in a few countries. These results have policy implications for the rapid scale-up of equitable life-saving cervical pre-cancer screening.

In this study, self-reported rate of cervical pre-cancer screening was lower than a third of the WHO target of screening 70% of eligible women for cervical pre-cancer by 2030. This finding is consistent with other studies conducted in the surveyed countries. Studies found cervical pre-cancer screening services uptake ranged from 13.5% in Ethiopia, 17.0 % in Tanzania, 19.0% in Zimbabwe to 26.5% in Malawi. In most countries, these findings were higher than our findings but nevertheless lower than 23%, or a third of 70% (6,7,28). The difference between our study estimates of cervical pre-cancer screening rates and other studies is that our outcome variable was self-reported cervical pre-cancer screening rate. Further, we used PHIA data, and defined cervical pre-cancer screening according to women's

For peer review only

For peer review only - http://bmjopen.bmj.com/site/about/guidelines.xhtml

self-reports of whether they had screened for cervical pre-cancer. A study in 18 resource constrained countries used the Demographic Health Survey (DHS) data. It defined cervical cancer services utilization as knowing about pap smear tests and vaginal cytology and their purpose and receiving educational information about cervical pre-cancer screening (6). Another reason is that we focused on women aged 25-49 years, whereas women outside this age group may have been included in cervical pre-cancer estimates of other studies (29). In Ethiopia, our estimates were from urban areas, whereas Ethiopia's pooled estimates of cervical pre-cancer screening included rural areas (7). There is an urgent need to scale up cervical pre-cancer screening, enabling women to access this life-saving service, and positioning the countries to meet the WHO cervical pre-cancer screening target by 2030.

Evidence supports the finding that inequalities in these countries, underserved with cervical pre-cancer screening, favoured women who were older, resided in urban areas, were highly educated, and wealthier, than women who were younger, living in rural areas, uneducated, and less wealthy (6,14,15). Studies highlight the heterogeneity in cervical pre-cancer screening with fewer women with lower or middle socioeconomic status accessing cervical pre-cancer screening than their wealthier counterparts (7,30,31,9,29,8,9,10,11,12). For example, in 18 resource-constrained countries, of which eight were from sub-Saharan Africa, high wealth status increased socioeconomic inequalities in cervical pre-cancer screening; living in urban communities reduced it (6). Further, older age, being literate, having at least a primary level education, and having a higher monthly household income, significantly increased the uptake of cervical pre-cancer screening in southern Malawi. The increases were greater in cervical cancer screening sites supported by Médecins Sans Frontières France (MSF) an international non-governmental organization (28). Private-government partnerships need to be formed in scaling up cervical pre-cancer screening, addressing the heterogeneous inequalities preventing women from accessing cervical pre-cancer screening.

For peer review only

For peer review only - http://bmjopen.bmj.com/site/about/guidelines.xhtml

This study found inequalities related to age, rural-urban residence, education, and wealth in cervical pre-cancer screening to be low or moderate in the surveyed countries which is supported by the existing evidence (6). One study found high inequalities in cervical pre-cancer screening between the poorest and richest socioeconomic status groups, although from a higher service utilization rate in high income countries than in resource constrained countries (6). For example, access to cervical pre-cancer screening service was 68.4% and 91.8% among women from the poorest and wealthiest socioeconomic status groups in Austria and 55.4% and 88.4% in Brazil. In in resource constrained countries, the corresponding rates were 18.8% and 40.2% respectively (6). These results suggest that inequalities may be present regardless of the coverage of the intervention. Our study further found that inequalities in cervical pre-cancer screening increased when combined suggesting that minor or moderate inequalities compound into high inequalities, further leaving some women behind in cervical pre-cancer screening. Inequalities related to age, rural-urban residence, education, and wealth, need to be addressed in eliminating socioeconomic inequalities in cervical pre-cancer screening.

We found gaps in cervical pre-cancer screening among women living with HIV. Not all eligible women living with HIV were screened cervical pre-cancer, which may be due to several reasons. One reason may be the perception that linking women testing HIV positive to HIV treatment eliminates the risk of cervical cancer for such women. A study in in South Africa and another in Zimbabwe found that many women living with HIV, including those on ART, screened positive for cervical pre-cancer, despite the countries attaining high HIV treatment coverage (3,32). Another reason women living with HIV may have not been screened for cervical pre-cancer is that they may have tested HIV positive at a facility with a limited capacity to conduct cervical pre-cancer screening. Alternatively, women living with HIV might have been unable to access health facilities with the capacity to test for cervical

For peer review only - http://bmjopen.bmj.com/site/about/guidelines.xhtml

pre-cancer for various reasons, including transport costs. Similarly, a large proportion of eligible HIV-negative women who were tested for HIV in this study were not screened for cervical pre-cancer. One reason is the perception that HIV–negative women may be at low risk of cervical pre-cancer (11).

Integrating cervical cancer screening into HIV, STI, maternal and reproductive health services is required and feasible (33,34). As an example, Rwanda vaccinated more than 90% of schoolgirls against HPV from 2011 to 2012, through designated "health days" which were adapted also for other sexual reproductive and maternal health purposes (35,36). This approach saved time, money, and costs compared with other methods of providing HPV vaccination for girls (35). However, the cervical pre-cancer screening rate in Rwanda in this study was low at 3.5% illustrating that cervical pre-cancer screening did not scale-up as did the HPV vaccination (36). Additional activities may include instituting same-day cervical pre-cancer screening and treatment (37), alleviating economic barriers to accessing cervical cancer screening, focusing outreach to women less likely to be screened and allowing women to self-collect samples (37,1,12).

Women's ability to benefit from cervical pre-cancer screening programmes may depend on their social health protection, such as whether they have childcare responsibilities that prevent them from accessing services (18). Women experiencing multiple inequalities depend on social protection and may be vulnerable under systems with low social protection (18). Expanding social protection coverage and enhancing the health system accessibility are crucial for women to access cervical pre-cancer screening (18).

In conclusion, cervical pre-cancer screening in the surveyed countries was low and inequitable – with an overall rate below a third of the WHO target to screen 70% of eligible women by 2030 for cervical pre-cancerous lesions. Women who were younger, lived in rural

For peer review only - http://bmjopen.bmj.com/site/about/guidelines.xhtml

areas, had no education and were from poor backgrounds were left behind in cervical pre-cancer screening. Age-related inequalities were the most common. In combination, inequalities related to age, residence, education, and wealth left younger, rural, uneducated, and poor women behind further. Focused cervical pre-cancer interventions are required to scale-up equitable cervical pre-cancer screening. Including equity in cervical pre-cancer screening programmes and monitoring it is required. Research is needed to understand how to scale-up equitable cervical pre-cancer screening in sub-Saharan African countries.

For peer review only

For peer review only - http://bmjopen.bmj.com/site/about/guidelines.xhtml

Availability of data and materials

Detailed descriptions of the PHIA survey design, sampling, variables, and analytic guidance are at the PHIA Project website at https://phia-data.icap.columbia.edu/files.

Abbreviations

HPV: Human Papilloma Virus

PHIA Population-Based HIV Impact Assessment

PEPFAR: President's Emergency Plan for AIDS Relief

UNAIDS: Joint United Nations Programme on HIV/AIDS

WHO: World Health Organisation

CI: Confidence intervals

STI: Sexually transmitted infection

For peer review only - http://bmjopen.bmj.com/site/about/guidelines.xhtml

414 # References

415

1. World Health Organization. Global strategy to accelerate the elimination of cervical cancer as a public health problem. Geneva: World Health Organization; 2020. Report No.: ISBN 978-92-4-001410-7.

2. World Health Organization. WHO guideline for screening and treatment of cervical pre-cancer lesions for cervical cancer prevention, second edition. Geneva: World Health Organization; 2021. Report No.: https://apps.who.int/iris/handle/10665/336583.

3. Rohner E, Bütikofer L, Schmidlin K, Sengayi M, Maskew M, Giddy J, et al. Cervical cancer risk in women living with HIV across four continents: A multicohort study. Int J Cancer. 2020 Feb 1; 146(3; doi: 10.1002/ijc.32260): 601-609.

4. Campos NG, Sharma M, Clark A, Lee K, Geng F, Regan C, et al. The health and economic impact of scaling cervical cancer prevention in 50 low- and lower-middle-income countries. Int J Gynaecol Obstet. 2017 Jul; Suppl 1(138 doi: 10.1002/ijgo.12184): 47-56.

5. Perehudoff K, Vermandere H, Williams A, Bautista-Arredondo S, Paepe ED, Dias S, et al. Universal cervical cancer control through a right to health lens: refocusing national policy and programmes on underserved women. BMC Int Health Hum Rights. 2020 July; 20(21. doi.org/10.1186/s12914-020-00237-9).

6. Mahumud RA, Keramat SA, Ormsby GM, Sultana M, Rawal LB, Alam K, et al. Wealth-related inequalities of women's knowledge of cervical cancer screening and service utilisation in 18 resource-constrained countries: evidence from a pooled decomposition analysis. International Journal for Equity in Health. 2020 March; 19(42 doi.org/10.1186/s12939-020-01159-7).

7. Ayenew AA, Zewdu BF, Nigussie AA. Uptake of cervical cancer screening service and associated factors among age-eligible women in Ethiopia: systematic review and meta-analysis. Infectious Agents and Cancer. 2020 November; 15(doi.org/10.1186/s13027-020-00334-3): 67.

8. Henke A, Kluge U, Borde T, Mchome B, Serventi F, Henke O. Tanzanian women´s knowledge about Cervical Cancer and HPV and their prevalence of positive VIA cervical screening results. Data from a Prevention and Awareness Campaign in Northern Tanzania, 2017 – 2019. Glob Health Action. 2021 Jan; 14(1 doi: 10.1080/16549716.2020.1852780): 1852780.

9. Weng Q, Jiang J, Haji FM, Nondo LH, Zhou H. Women's knowledge of and attitudes toward cervical cancer and cervical cancer screening in Zanzibar, Tanzania: a cross-sectional study. BMC Cancer. 2020 January; 20(doi.org/10.1186/s12885-020-6528-x): 63.

10. Barrow A, Onikan A, Nzoputam CI, Ekholuenetale M. Prevalence and determinants of cervical cancer awareness among women of reproductive age: evidence from Benin and Zimbabwe population-based data. Appl Cancer Res. 2020 October; 40(doi.org/10.1186/s41241-020-00092-z).

11. Moucheraud C, Kawale P, Kafwafwa S, Bastani R, Hoffma RM. "It is big because it's ruining the lives of many people in Malawi": Women'sattitudes and beliefs about cervical cancer. Preventive Medicine Reports. 2020 June; 18(DOI: 10.1016/j.pmedr.2020.101093): 101093.

For peer review only - http://bmjopen.bmj.com/site/about/guidelines.xhtml

12. Pry JM, Manasyan A, Kapambwe S, Taghavi K, Duran-Frigola M, Mwanahamuntu M, et al. Cervical cancer screening outcomes in Zambia, 2010–19: a cohort study. The Lancet Global Health. 2021 June; 9(6 DOI:https://doi.org/10.1016/S2214-109X(21)00062-0): E832-E840.

13. Nyambe A, Kampen JK, Baboo SK, Hal GV. Knowledge, attitudes and practices of cervical cancer prevention among Zambian women and men. BMC Public Health 19, 508 (2019). https://doi.org/10.1186/s12889-019-6874-2. BMC Public Health. 2019 May; 19(508 doi.org/10.1186/s12889-019-6874-2).

14. Akanda R, Kawale P, Corrina M. Cervical cancer prevention in Africa: A policy analysis. Journal of Cancer Policy. 2022 January;(https://doi.org/10.1016/j.jcpo.2021.100321).

15. Akokuwebe ME, Idemudia ES, Lekulo AM, Motlogeloa OW. Determinants and levels of cervical Cancer screening uptake among women of reproductive age in South Africa: evidence from South Africa Demographic and health survey data, 2016. BMC Public Health. 2021 November; 21(1, doi.org/10.1186/s12889-021-12020-z): 2013.

16. Keetile M, Ndlovu K, Letamo G, Disang M, Yaya S, Navaneetham K. Factors associated with and socioeconomic inequalities in breast and cervical cancer screening among women aged 15–64 years in Botswana. PLoS ONE. 2021 August; 16(8 doi.org/10.1371/journal.pone.0255581): e0255581.

17. Quizhpe E, Sebastian MS, Teran E, Pulkki-Brännström AM. Socioeconomic inequalities in women's access to health care: has Ecuadorian health reform been successful? International Journal for Equity in Health. 2020 October; 19(doi.org/10.1186/s12939-020-01294-1): 178.

18. Prez VD, Jolidon V, Willems B, Cullati S, Burton-Jeangros C, Bracke P. Cervical cancer screening programs and their context-dependent effect on inequalities in screening uptake: a dynamic interplay between public health policy and welfare state redistribution. Int J Equity Health. 20 September; 20(doi.org/10.1186/s12939-021-01548-6): 211.

19. Jolidon V, Prez VD, Willems B, Bracke P, Cullati S, Burton-Jeangros C. Never and under cervical cancer screening in Switzerland and Belgium: trends and inequalities. BMC Public Health. 2020 October; 20(doi.org/10.1186/s12889-020-09619-z): 1517.

20. Stelzle D, Tanaka LF, Lee KK, Khalil AI, Baussano I, Shah ASV, et al. Estimates of the global burden of cervical cancer associated with HIV. Lancet Glob Health. 2021 February; 9(2. doi.org/10.1016/S2214-109X(20)30459-9): E161-E169.

21. World health Organizaation (WHO). Health Equity. [Online].; 2021 [cited 2021 November 10. Available from: https://www.who.int/health-topics/health-equity#tab=tab_1.

22. Emadi M, Delavari S, Bayati M. Global socioeconomic inequality in the burden of communicable and non-communicable diseases and injuries: an analysis on global burden of disease study 2019. BMC Public Health. 2021 September; 21(https://doi.org/10.1186/s12889-021-11793-7): 1771.

23. O'Donnell O, Doorslaer Ev, Wagstaff A, Lindelow M. Analyzing health equity using Household Survey Data. A guide to techniques and their Implementation Washington DC: The International Bank for Reconstruction and Development / The World Bank; 2008.

For peer review only

For peer review only - http://bmjopen.bmj.com/site/about/guidelines.xhtml

24. World Health Organization. State of inequality: HIV, tuberculosis and malaria. Geneva: World Health Organization; 2021. Report No.: Licence: CC BY-NC-SA 3.0 IG.

25. McKenzie D. World Bank Blogs. Published on Development Impact. [Online].; 2020 [cited 2022 June 2022 19. Available from: https://blogs.worldbank.org/impactevaluations/overview-multiple-hypothesis-testing-commands-stata.

26. Population-Based HIV Impact Assessment (PHIA) Data Use Manual. New York:; July 2019.

27. Population-Based HIV Impact Assessments (PHIA) Project. Population-Based HIV Impact Assessments (PHIA) Guiding the Global AIDS Response. [Online].; 2021 [cited 2021 February 22. Available from: https://phia-data.icap.columbia.edu/files.

28. Gerstl S, Lee L, Nesbitt RC, Mambula C, Sugianto H, Phiri T, et al. Cervical cancer screening coverage and its related knowledge in southern Malawi. BMC Public Health. 2022 Feb; 295(Doi.org/10.1186/s12889-022-12547-9).

29. Kapambwe S, Sahasrabuddhe VV, Blevins M, Mwanahamuntu MH, Mudenda V, Shepherd BE, et al. Implementation and Operational Research: Age Distribution and Determinants of Invasive Cervical Cancer in a "Screen-and-Treat" Program Integrated With HIV/AIDS Care in Zambia. J Acquir Immune Defic Synd. 2015 Sep; 70(1. doi: 10.1097/QAI.0000000000000685): e20-6..

30. Niyonsenga G, Gishoma D, Sego R, Uwayezu MG, Nikuze B, Fitch M, et al. Knowledge, utilization and barriers of cervical cancer screening among women attending selected district hospitals in Kigali - Rwanda. Canadian Oncology Nursing Journal. 2021 July; 31(3. doi: 10.5737/23688076313266274): 266–274.

31. Ndateba I, Kabatsinda A, Ndabarora E. Uptake of Cervical Cancer Screening and Associated Factors Among Women Attending Outpatient Services in Rwamagana Hospital, Rwanda. Rwanda Journal of Medicine and Health Sciences. 2021 December; 4(3. doi: 10.4314/rjmhs.v4i3.8).

32. Taghavi K, Mandiriri A, Shamu T, Rohner E, Bütikofer L, Asangbeh S, et al. Cervical Cancer Screening Cascade for women living with HIV: A cohort study from Zimbabwe. PLOS Glob Public Health. 2022 Feb; 2(2. https://doi.org/10.1371/journal.pgph.0000156): e0000156.

33. Parham GP, MwanahamuntuV MH, Kapambwe S, Muwonge R, Bateman AC, Blevins M, et al. Population-level scale-up of cervical cancer prevention services in a low-resource setting: Development, implementation, and evaluation of the cervical cancer prevention program in Zambia. PLoS ONE. 2015; 10(4. doi.org/10.1371/journal.pone.0122169).

34. Pfaff C, Singano V, Akello H, Amberbir A, Berman J, Kwekwesa A, et al. Early experiences in integrating cervical cancer screening and treatment into HIV services in Zomba Central Hospital, Malawi. Malawi Med J. 2018 Sep; 30(3: doi: 10.4314/mmj.v30i3.14.): 211-214.

35. Binagwaho A, Ngabo F, Wagner CM, Gatera M, Nutt CT, Nsanzimana S. Lessons from the field. Bulletin of the World Health Organisation. 2013; 91(doi: http://dx.doi.org/10.2471/BLT.12.116087): 697–703.

36. Muhimpundu MA, Ngabo F, Sayinzoga F, Balinda JP, Rusine J, Harward S, et al. Screen, Notify, See, and Treat: Initial Results of Cervical Cancer Screening and Treatment in Rwanda. JCO Global Oncology. 2021; 7(DOI: 10.1200/GO.20.00147): 632-638.

For peer review only - http://bmjopen.bmj.com/site/about/guidelines.xhtml

37. Campos NG, VT, Jeronimo J, Mvundura M, Kim JJ. Evidence-based policy choices for efficient and equitable cervical cancer screening programs in low-resource settings. Cancer Medicine. 2017 May; 6(8. doi: 10.1002/cam4.1123): 2008–2014.

Acknowledgements

The authors thank UNAIDS for the in-kind support provided in drafting this manuscript. However, the views in this article are those of the authors.

Funding

Olivia Keiser was funded by SNF grant number 202660.

Author information

Institute of Global Health, University of Geneva, Geneva, Switzerland.
David, Chipanta, Janne Estill, Olivia Keiser

Joint United Nations Programme on HIV/AIDS (UNAIDS).
David Chipanta

World Health Organisation.
Sharon Kapambwe

School of Global and Public Health, Kamuzu University of Health Sciences.
Alinane Linda Nyondo-Mipando

Newlands Clinic, Harare, Zimbabwe.
Margaret Pascoe

Department of Economics, University of Lausanne, Lausanne, Switzerland.
Silas Amo-Agyei

Swiss Tropical and Public Health Institute, Basel, Switzerland.

For peer review only

University of Basel, Allschwil, Switzerland
Julia Bohlius

Contributions

DC designed the study. DC and SA collated the data. DC, SA, SK, ALNM, MP, JB, JE, and OK performed the data analysis. DC conducted the data visualisation, and drafted, and revised the manuscript. SA, SK, ALNM, MP, JB, JE, and OK critically reviewed the drafts. All authors approved the final manuscript.

Corresponding author: Email: chipanta@hotmail.com; david.chipanta@etu.unige.ch

Ethics declarations

The study did not need ethical clearance because the data is de-identified and publicly available at https://phia-data.icap.columbia.edu/files.

Consent for publication

Not applicable.

Competing interests

The authors declare that they have no competing interests.

For peer review only

Figure 1

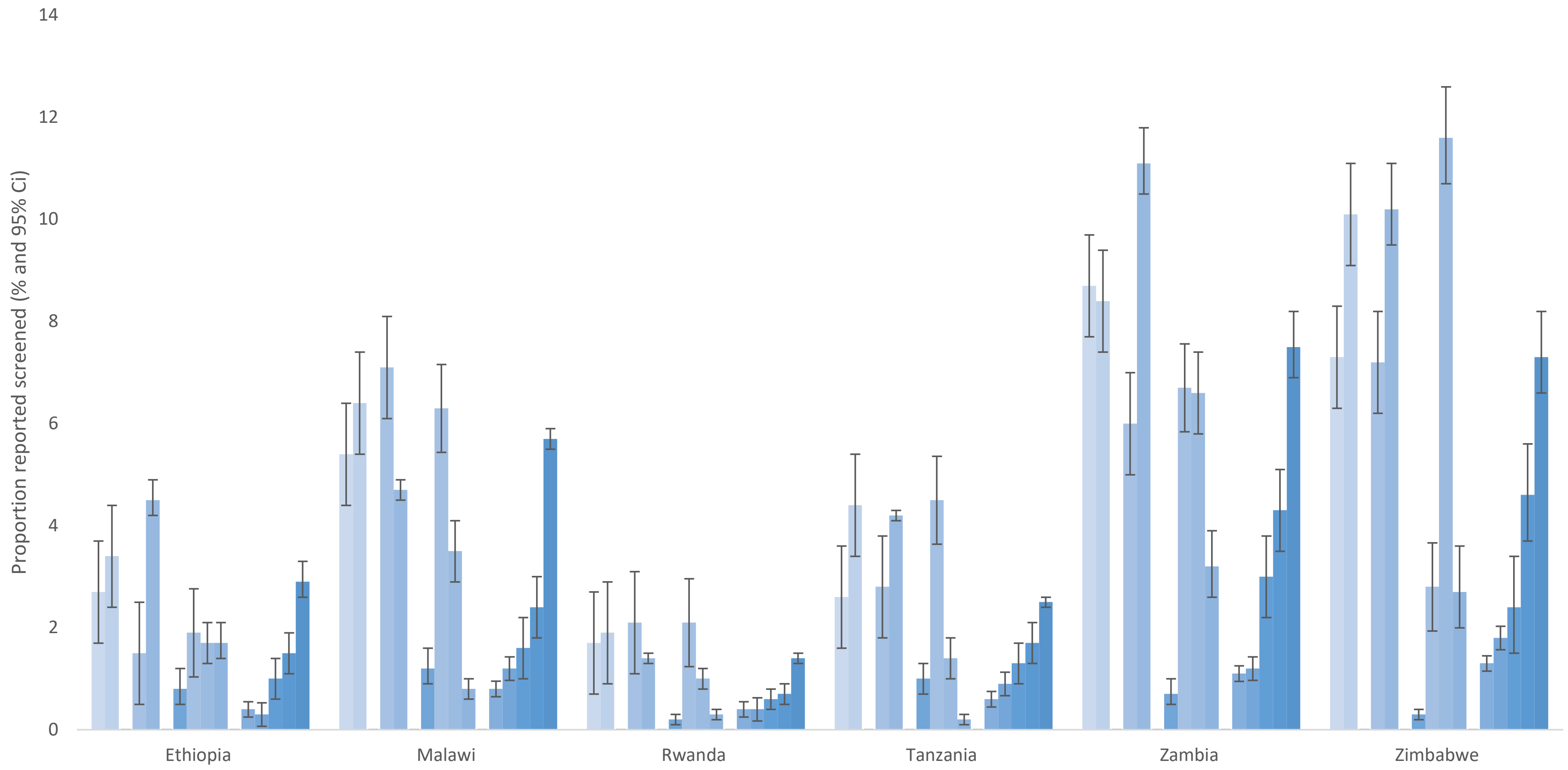
14
12
10
8
6
4
2
0
Proportion reported screened (% and 95% Ci)
Ethiopia
Malawi
Rwanda
Tanzania
Zambia
Zimbabwe

Figure 2

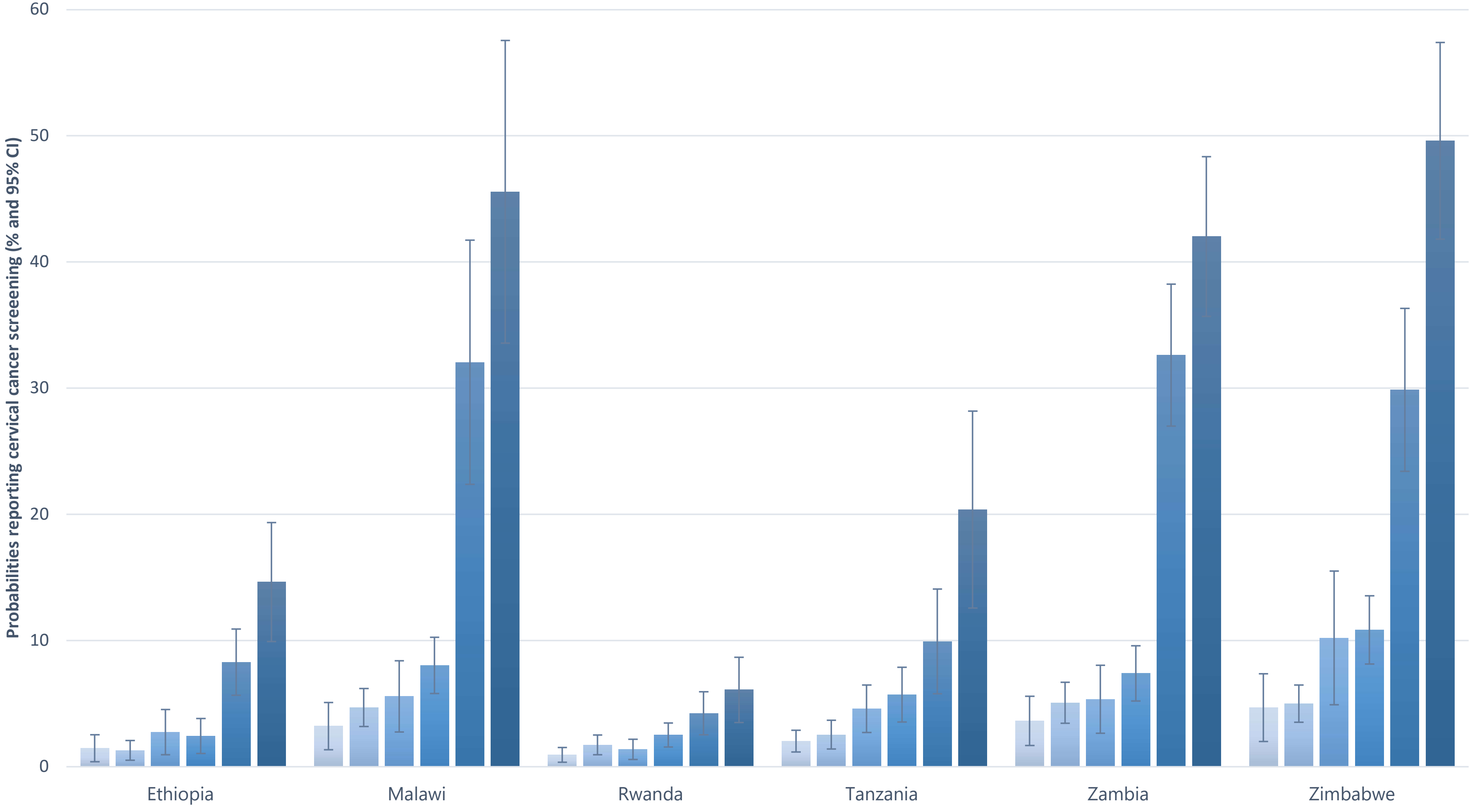
60
50
40
30
20
10
0
Probabilities reporting cervical cancer screeening (% and 95% CI)
Ethiopia
Malawi
Rwanda
Tanzania
Zambia
Zimbabwe

Supplementary Table 1. Variable descriptions.

Variable name	Description	Measure
Cervical pre-cancer screening	Self-reported from the adult interview question on ever testing for cervical pre-cancer, coded no, yes.	Binary
Sex	Self-reported sex (i.e., male, female).	Binary
Age	Self-reported linear years categorized in years (25–34, 35–49).	Categorical
Location, rural, urban	Self-reported residence (i.e., rural, urban), from the household interview. The data from Ethiopia had only urban variables. For Ethiopia, we coded size of area <=50,000 as rural = 0, and >50k =1; urban, from the household data set.	Binary
Ever married	Was derived from the question 'Have you ever been married or lived together with a (man/woman) as if married?' coded no, yes.	Binary
Ever tested for HIV	From the question 'Has been tested for HIV', coded never tested, ever tested.	Binary
HIV prevalence	The proportion of people 15 years or older testing HIV positive using laboratory-confirmed HIV test results from the adult biomarker questionnaire.	Binary
Educational status	Level of school respondent ever attended (i.e., not educated, primary school education, secondary school education, or higher education, from the adult interview. Tanzania's educations variables were standardized as follows: No education; Primary combined pre-primary, primary and post-primary; Secondary combined secondary, post-secondary training, secondary A); More than secondary (included post-secondary A levels and university).	Categorical
Wealth	Assessed household wealth with the wealth quintiles variable, which was derived from a wealth index. Wealth quintiles ranked households depending on their household characteristics and asset ownership from Q1 representing the poorest households to Q5 representing the wealthiest. (i.e., poorest, poor, rich, richer, and richest). The variable is provided in the PHIA household data set.	Ordered ranking
Regions, province, or zone	Defined as a political area, which is the primary sampling unit. It was province for Rwanda, Zambia, and Zimbabwe; region for Ethiopia, zone for Malawi, and mainlandzanziba for Tanzania.	Categorical

Note: Unless indicated the variables were from the adult interview data set. The answer options 'didn't know' or 'refused to answer' were coded as missing for all variables.

For peer review only - http://bmjopen.bmj.com/site/about/guidelines.xhtml

Reporting checklist for quality improvement in health care.

Based on the SQUIRE guidelines.

Instructions to authors

Complete this checklist by entering the page numbers from your manuscript where readers will find each of the items listed below.

Your article may not currently address all the items on the checklist. Please modify your text to include the missing information. If you are certain that an item does not apply, please write "n/a" and provide a short explanation.

Upload your completed checklist as an extra file when you submit to a journal.

In your methods section, say that you used the SQUIREreporting guidelines, and cite them as:

Ogrinc G, Davies L, Goodman D, Batalden P, Davidoff F, Stevens D. SQUIRE 2.0 (Standards for QUality Improvement Reporting Excellence): revised publication guidelines from a detailed consensus process

		Reporting Item	Page Number
**Title**			
	#1	Indicate that the manuscript concerns an initiative to improve healthcare (broadly defined to include the quality, safety,	1

For peer review only

		effectiveness, patientcenteredness, timeliness, cost, efficiency, and equity of healthcare)	
**Abstract**			
	#02a	Provide adequate information to aid in searching and indexing	2
	#02b	Summarize all key information from various sections of the text using the abstract format of the intended publication or a structured summary such as: background, local problem, methods, interventions, results, conclusions	2
**Introduction**			
Problem description	#3	Nature and significance of the local problem	3
Available knowledge	#4	Summary of what is currently known about the problem, including relevant previous studies	3
Rationale	#5	Informal or formal frameworks, models, concepts, and / or theories used to explain the problem, any reasons or assumptions that were used to develop the intervention(s), and reasons why the intervention(s) was expected to work	3
Specific aims	#6	Purpose of the project and of this report	4
**Methods**			
Context	#7	Contextual elements considered important at the outset of introducing the intervention(s)	5

For peer review only

Intervention(s)	#08a	Description of the intervention(s) in sufficient detail that others could reproduce it	N/A
Intervention(s)	#08b	Specifics of the team involved in the work	N/A
Study of the Intervention(s)	#09a	Approach chosen for assessing the impact of the intervention(s)	5
Study of the Intervention(s)	#09b	Approach used to establish whether the observed outcomes were due to the intervention(s)	6
Measures	#10a	Measures chosen for studying processes and outcomes of the intervention(s), including rationale for choosing them, their operational definitions, and their validity and reliability	6
Measures	#10b	Description of the approach to the ongoing assessment of contextual elements that contributed to the success, failure, efficiency, and cost	N/A
Measures	#10c	Methods employed for assessing completeness and accuracy of data	6
Analysis	#11a	Qualitative and quantitative methods used to draw inferences from the data	6
Analysis	#11b	Methods for understanding variation within the data, including the effects of time as a variable	N/A
Ethical considerations	#12	Ethical aspects of implementing and studying the intervention(s) and how they were addressed, including, but	20

For peer review only

		not limited to, formal ethics review and potential conflict(s) of interest	

Results

	#13a	Initial steps of the intervention(s) and their evolution over time (e.g., time-line diagram, flow chart, or table), including modifications made to the intervention during the project	N/A
	#13b	Details of the process measures and outcome	6-9
	#13c	Contextual elements that interacted with the intervention(s)	6-9
	#13d	Observed associations between outcomes, interventions, and relevant contextual elements	6-9
	#13e	Unintended consequences such as unexpected benefits, problems, failures, or costs associated with the intervention(s).	N/A
	#13f	Details about missing data	6

Discussion

Summary	#14a	Key findings, including relevance to the rationale and specific aims	9
Summary	#14b	Particular strengths of the project	14
Interpretation	#15a	Nature of the association between the intervention(s) and the outcomes	N/A
Interpretation	#15b	Comparison of results with findings from other publications	10-11

For peer review only

Interpretation	#15c	Impact of the project on people and systems	N/A
Interpretation	#15d	Reasons for any differences between observed and anticipated outcomes, including the influence of context	12 - 14
Interpretation	#15e	Costs and strategic trade-offs, including opportunity costs	N/A
Limitations	#16a	Limits to the generalizability of the work	14
Limitations	#16b	Factors that might have limited internal validity such as confounding, bias, or imprecision in the design, methods, measurement, or analysis	14
Limitations	#16c	Efforts made to minimize and adjust for limitations	14
Conclusion	#17a	Usefulness of the work	14
Conclusion	#17b	Sustainability	N/A
Conclusion	#17c	Potential for spread to other contexts	14
Conclusion	#17d	Implications for practice and for further study in the field	14
Conclusion	#17e	Suggested next steps	14
**Other information**			
Funding	#18	Sources of funding that supported this work. Role, if any, of the funding organization in the design, implementation, interpretation, and reporting	2

For peer review only

None The SQUIRE 2.0 checklist is distributed under the terms of the Creative Commons Attribution License CC BY-NC 4.0. This checklist can be completed online using https://www.goodreports.org/, a tool made by the EQUATOR Network in collaboration with Penelope.ai

For peer review only

BMJ Open

Socioeconomic inequalities in cervical pre-cancer screening among women in Ethiopia, Malawi, Rwanda, Tanzania, Zambia, and Zimbabwe – Analysis of Population-Based HIV Impact Assessment surveys.

Journal:	*BMJ Open*
Manuscript ID	bmjopen-2022-067948.R2
Article Type:	Original research
Date Submitted by the Author:	09-May-2023

Complete List of Authors:	Chipanta, David; University of Geneva, Faculty of Medicine; UNAIDS, ERA<br>Kapambwe, Sharon; World Health Organisation<br>Nyondo-Mipando, Alinane Linda; Kamuzu University of Health Sciences<br>Pascoe, Margaret; Newlands Clinic, Clinic<br>Amo-Agyei, Silas; University of Lausanne, Department of Economics<br>Bohlius, Julia; University of Bern, Swiss Tropical and Public Health Institute<br>Estill, Janne; University of Geneva<br>Keiser, Olivia ; University of Geneva

**Primary Subject Heading**:	Epidemiology
Secondary Subject Heading:	Health economics, Health policy, Health services research
Keywords:	Cancer pain < ONCOLOGY, HIV & AIDS < INFECTIOUS DISEASES, PUBLIC HEALTH, Health policy < HEALTH SERVICES ADMINISTRATION & MANAGEMENT, Health economics < HEALTH SERVICES ADMINISTRATION & MANAGEMENT, Reproductive medicine < GYNAECOLOGY

SCHOLARONE™ Manuscripts

BMJ

I, the Submitting Author has the right to grant and does grant on behalf of all authors of the Work (as defined in the below author licence), an exclusive licence and/or a non-exclusive licence for contributions from authors who are: i) UK Crown employees; ii) where BMJ has agreed a CC-BY licence shall apply, and/or iii) in accordance with the terms applicable for US Federal Government officers or employees acting as part of their official duties; on a worldwide, perpetual, irrevocable, royalty-free basis to BMJ Publishing Group Ltd ("BMJ") its licensees and where the relevant Journal is co-owned by BMJ to the co-owners of the Journal, to publish the Work in this journal and any other BMJ products and to exploit all rights, as set out in our licence.

The Submitting Author accepts and understands that any supply made under these terms is made by BMJ to the Submitting Author unless you are acting as an employee on behalf of your employer or a postgraduate student of an affiliated institution which is paying any applicable article publishing charge ("APC") for Open Access articles. Where the Submitting Author wishes to make the Work available on an Open Access basis (and intends to pay the relevant APC), the terms of reuse of such Open Access shall be governed by a Creative Commons licence – details of these licences and which Creative Commons licence will apply to this Work are set out in our licence referred to above.

Other than as permitted in any relevant BMJ Author's Self Archiving Policies, I confirm this Work has not been accepted for publication elsewhere, is not being considered for publication elsewhere and does not duplicate material already published. I confirm all authors consent to publication of this Work and authorise the granting of this licence.

For peer review only

Socioeconomic inequalities in cervical pre-cancer screening among women in Ethiopia, Malawi, Rwanda, Tanzania, Zambia, and Zimbabwe – Analysis of Population-Based HIV Impact Assessment surveys.

David Chipanta[1,2] *, Sharon Kapambwe[3], Alinane Linda Nyondo-Mipando[4], Margaret Pascoe[5] Silas Amo-Agyei[6], Julia Bohlius[78], Janne Estill[1], Olivia Keiser[1]

1 Institute of Global Health, University of Geneva, Geneva, Switzerland.
2 Joint United Nations Programme on HIV/AIDS (UNAIDS), Geneva, Switzerland.
3 World Health Organisation, Harare, Zimbabwe.
4 School of Global and Public Health, Kamuzu University of Health Sciences, Blantyre, Malawi.
5 Newlands Clinic, Harare, Zimbabwe.
6 Department of Economics, University of Lausanne, Lausanne, Switzerland.
7 Swiss Tropical and Public Health Institute, Basel, Switzerland.
8 University of Basel, Basel, Switzerland

*Corresponding author: Email: chipanta@hotmail.com; david.chipanta@etu.unige.ch

E-mail addresses of co-authors:
SK: kapambwes@who.int
ALNM- lmipando@kuhes.ac.mw
MP MargaretP@newlandsclinic.org.zw
SA: silas.amo-agyei@unil.ch
JB julia.bohlius@swisstph.ch
JE: Janne.Estill@unige.ch
OK: Olivia.Keiser@unige.ch

Where authors are identified as personnel of the International Agency for Research on Cancer / World Health Organization, or UNAIDS, the authors alone are responsible for the views expressed in this article and they do not necessarily represent the decisions, policy, or views of the International Agency for Research on Cancer / World Health Organization or UNAIDS.

For peer review only - http://bmjopen.bmj.com/site/about/guidelines.xhtml

Abstract

Objectives: We examined age, residence, education and wealth inequalities and their combinations on cervical pre-cancer screening probabilities for women. We hypothesised that inequalities in screening favoured women who were older, lived in urban areas, were more educated and wealthier.

Design: Cross-sectional study using Population-Based HIV Impact Assessment data.

Setting: Ethiopia, Malawi, Rwanda, Tanzania, Zambia, and Zimbabwe. Differences in screening rates were analysed using multivariable logistic regressions, controlling for age, residence, education, and wealth. Inequalities in screening probability were estimated using marginal effects models.

Participants: Women aged 25–49 years, reporting screening.

Outcome measures: Self-reported screening rates, and their inequalities in percentage points, with differences of 20%+ defined as high inequality, 5% to 20% as medium, 0 to 5% as low.

Results: The sample size of participants ranged from 5882 in Ethiopia to 9186 in Tanzania. The screening rates were low in the surveyed countries, ranging from 3.5% (95% Confidence Interval (CI) 3.1%–4.0%) in Rwanda to 17.1% (95% CI 15.8%–18.5%) and 17.4% (95% CI 16.1%–18.8%) in Zambia and Zimbabwe. Inequalities in screening rates were low based on covariates. Combining the inequalities led to significant inequalities in screening probabilities between women living in rural areas aged 25–34 years, with a primary education level, from the lowest wealth quintile, and women living in urban areas aged 35–49 years, with the highest education level, from the highest wealth quintile, ranging from 4.4% in Rwanda to 44.6% in Zimbabwe.

Conclusions: Cervical pre-cancer screening rates were inequitable and low. No country surveyed achieved one-third of the World Health Organisation's target of screening 70% of eligible women by 2030. Combining inequalities led to high inequalities, preventing women who were younger, lived in rural areas, were uneducated, and from the lowest wealth quintile from screening. Governments should include and monitor equity in their cervical pre-cancer screening programmes.

Strength and limitations of this study

- Collinearity was present, especially between the wealth and residence variables which tend to correlate with each other, consistent with how the wealth variable is constructed.
- Recall bias was present as cervical pre-cancer screening is self-reported.
- We did not control for the type of cervical pre-cancer screening because we did not have information on the types of screening women received.

For peer review only

Introduction

Although all countries are affected by cervical pre-cancer, its incidence and mortality are more than twice and three times as high in low- and middle-income countries as in high-income countries (1). In 2020, it was the leading cause of cancer deaths in 36 countries. Most of these countries were in sub-Saharan Africa, Melanesia, South America, and South-Eastern Asia (2). Cervical cancer occurs in the lower part of the uterus that connects to the vagina. It is caused in over 70% of cases by the human papillomavirus (HPV), the most common sexually transmitted infection (STI) (1). Most HPV strains are harmless in people with a healthy immune system (1). HIV-positive women, whose immunity may be compromised (1,2) are six times more likely to develop cervical cancer than HIV negative women (1). In 2014, invasive cervical cancer was 11 times higher among women living with HIV in South Africa than in Europe five years after antiretroviral therapy (ART) initiation (3). Cervical cancer harms girls, women and their families with significant health and development consequences, yet it is preventable and curable if detected and treated early (1,2). HPV vaccination and regular pre-cancer screening stop cervical pre-cancer. Cervical pre-cancer screening identifies pre-cancerous cells to treat before they become pre-cancer (1). Comprehensive HPV vaccination and cervical pre-cancer screening could prevent an estimated 5.2 million cases and 3.7 million deaths over 10 years and would cost around US $3.2 billion in 50 low and lower-middle-income countries (4). In 2020, the World Health Organisation (WHO) proposed the 90–70–90 target, which, if achieved by 2030, would put the world on course to eliminate cervical cancer (1). The 90–70–90 target seeks to ensure that 90% of girls are vaccinated against HPV by age 15 years, 70% of eligible women are screened by age 35 years and again by 45, and 90% of women identified with cervical cancer receive treatment (1).

For peer review only

Cervical cancer is a disease of inequality. Its burden reflects inequalities in society, including those related to socioeconomic status. Inequality in health and health care is defined as the unequal distribution of health goods, services and outcomes across income or other measures of wealth. In contrast, equity is the lack of avoidable and remedial differences in the distribution of health goods and services or outcomes due to people's socioeconomic status in society (5,6,7). Cervical pre-cancer screening is a critical component of the 90–70–90 target (8). Socioeconomic inequalities are preventing women from accessing cervical pre-cancer screening. Studies show that residence, geography, education, wealth, age, health insurance status, and the capacity of health systems influence access to cervical pre-cancer screening. Other factors include, a history of multiple sexual partners, HIV-positive status and women's social interactions (9,10,11,12,13,14,15,16,17,18). These studies showed that women who were older, resided in urban areas, and were more educated and wealthier were screened more often than younger, rural residents, uneducated, and poor women, with a few exceptions (9,19,20,21,22). However, these studies did not estimate the magnitude of inequalities and their combined impact on cervical pre-cancer screening. Such studies are needed to scale up cervical pre-cancer screening and support the prioritization of inequalities to eliminate cervical cancer, especially in east and southern African countries, where HIV has worsened the cervical cancer burden (23). This study aimed to examine age, residence, education, and wealth inequalities and their combined impacts on cervical pre-cancer screening in Ethiopia, Malawi, Rwanda, Tanzania, Zambia, and Zimbabwe. We hypothesized that significant inequalities in cervical pre-cancer screening rates would favour older women, women who resided in urban areas, and more educated and wealthy women.

Methods

Data and sample

For peer review only - http://bmjopen.bmj.com/site/about/guidelines.xhtml

This is cross-sectional study of a secondary data analysis of available PHIA data for countries that included cervical pre-cancer screening variables. The countries and the years in which the PHIA surveys were conducted include Ethiopia (2017–2018) (24), Malawi (2015–2016) (25), Rwanda (2018–2019) (26), Tanzania (2016–2017) (27), Zambia (2016) (28) and Zimbabwe (2015–2016) (29). The PHIA surveys collected a range of health and socio-demographic data to evaluate HIV the impact of HIV programs in countries supported by United States President's Emergency Plan for AIDS Relief (PEPFAR). We used the Household, and Adult data sets. In participating households, a household questionnaire was administered to the head-of-household, who provided information on the household relating to wealth, and other socio demographic characteristics. Then, an individual questionnaire was administered to eligible and consenting adults aged 15 years or older in the household. The individual interviews assessed a range of HIV-related variables, including cervical pre-cancer screening and HIV testing.

We restricted our analysis to women aged 25–49 years who responded to the cervical pre-cancer questions. This is the group WHO recommends for cervical pre-cancer screening – ages 30–49 years for the general population and 25–49 years for women living with HIV – including all gender diverse people at risk of cervical cancer (1).

Patient and Public Involvement

No patient involved.

Variables and outcome descriptions

For peer review only - http://bmjopen.bmj.com/site/about/guidelines.xhtml

We assessed inequalities related to age, residence, education, and wealth in cervical pre-cancer screening according to women's self-reports. These dimensions of inequalities are familiar sources of disadvantage or discrimination (30). Our primary outcome was self-reported ever testing for cervical pre-cancer. The main predictors were age, residence, education, and wealth. Other covariates were having ever married, ever been tested for HIV, (also binary coded), and regions. Regions, provinces, or zones were coded as dummy variables and included to capture the geographical variation in cervical pre-cancer screening. Ethiopia's data set had an urban variable, which we coded as rural if the population size was smaller than 50,000 people, and urban otherwise. Supplementary Table 1 describes the variables. We used percentages to measure the magnitude of inequality between subgroups. We denoted a difference of 20% or more between two subgroups as high inequality, less than 20% but greater than 5% as medium inequality and below 5% as low inequality, as used by the WHO (30).

Analysis

We assessed inequalities in self-reported cervical pre-cancer screening in three steps. For each country, we first presented sample characteristics descriptively. Second, we determined the associations between screening for cervical pre-cancer and each dimension of inequality, and analysed the differences using multivariable logistics regression, controlling for covariates. We corrected the p-values of odds ratios for multiple hypothesis testing with sharpened q's. In step 3, we used marginal effects models to estimate the probabilities of reporting cervical pre-cancer screening for each inequality alone and combined with other inequalities, holding the other inequalities constant at their mean values. We applied survey weights to account for complex survey design. Using jack-knife replicate weights, 95% confidence intervals (CI) were estimated (31). We excluded the observation from the analysis if the gender value was missing. We did not impute the missing data since all variables had

For peer review only - http://bmjopen.bmj.com/site/about/guidelines.xhtml

less than 2% of missing values. We used Stata version 14 for the analyses. The study did not require ethical clearance. The data are publicly available and de-identified at https://phia-data.icap.columbia.edu/files (32). This study conforms to the SQUIRE reporting guidelines (33).

Results

The sample size of females aged 25–49 years ranged from 5882 in Ethiopia to 9186 in Tanzania. The median age and interquartile ranges were 32 years (28–39) in Ethiopia, 33 years (29–40) in Zambia and 34 years (29–40) in Malawi, Rwanda, and Tanzania. The rate of self-reported cervical pre-cancer screening was lowest in Rwanda (3.5% [95% CI 3.1%–4.0%]) and highest in Zambia (17.1% [95% CI 15.8%–18.5%]) and Zimbabwe (17.4% [95% CI 16.1%–18.8%]). The HIV prevalence among 25–49-year-old females ranged from 4.7% (95% CI 4.1%–5.4%) in Rwanda to 22.6% (95% CI 21.6%–23.7%) in Zimbabwe, and the rate of cervical pre-cancer screening among HIV-positive females ranged from 0.4% (95% CI 0.3%–0.5%) in Rwanda to 5.4% (95% CI 4.7%–6.1%) in Zambia. Females aged 25 to 34 years comprised half or more of the sample in surveyed countries. Eighty-six percent or more of the respondents reported ever have been married, and 86% or more reported ever have been tested for HIV. More than half of the respondents resided in rural areas, excluding Ethiopia, where all respondents resided in urban areas, 54% of them in areas with <50,000 residents. Less than 20% of the respondents were uneducated, and nearly 30% had attained a primary school education. Only in Ethiopia, Malawi and Zambia was the proportion of respondents in the top two wealth quintiles greater than that in the bottom two quintiles (Table 1).

For peer review only - http://bmjopen.bmj.com/site/about/guidelines.xhtml

Table 1: Survey weighted sample characteristics by country (PHIA 2015–2019), (percentage [95% Confidence Interval] sample size)

	Ethiopia	Malawi	Rwanda	Tanzania	Zambia	Zimbabwe
**CC screened**	6.1 (5.3–6.9),343	11.8 (10.6–13.1),926	3.5 (3.1–4.0),353	7.0 (6.1–8.1),586	17.1 (15.8–18.5),1066	17.4 (16.1–18.8),1129
**HIV positive**	6.1 (5.3–7.0),362	18.8 (17.3–20.3),1152	4.7 (4.1–5.4),416	9.2 (8.4–10.0),897	21.2 (19.8–22.6),1263	22.6 (21.6–23.7),1602
Cervical Pre-cancer screened	0.9 (0.7–1.45),52	3.4 (2.8–4.1), 249	0.4 (0.3–0.5),43‡	1.5 (1.1 –2.0),132	5.4 (4.7–6.1),318	4.5 (4.0–5.0),305
**Age** (years): Median [IQR] years	32 (28–39)	34 (29–40)	34 (29–40)	34 (29–40)	33 (29–40)	34 (29-40)
25–34	55.9 (55.7–56.2),3517	53.0 (52.9–53.1),3342	52.1 (52.1–52.2),4508	52.2 (52.2–52.3),4812	55.3 (55.2–55.4),3386	54.5 (54.4–54.6),3403
35–49	44.1 (43.8–44.3),2365	47.0 (46.9–47.1),2720	47.9 (47.8–47.9),4063	47.8 (47.7–47.8),4374	44.7 (44.6–44.8),3027	45.5 (45.4–45.6),3381
**Ever married**	89.7 (88.4–90.9),5228	96.6 (95.9–97.1),5794	86.0 (85.0–87.0),7320	92.6 (91.6–93.6),8565	91.7 (90.6–92.7),5901	94.0 (93.3–94.6),6373
**Ever tested for HIV**	86.4 (84.9–87.7),5018	94.8 (94.0–95.4),5756	95.7 (95.2–96.1),8194	89.2 (88.2–90.1),8206	92.3 (91.5–93.1),5878	94.8 (94.1–95.4),6375
**Location** Urban	54.0 (48.8–59.1),3215	19.0 (16.3–21.9),2289	20.2 (16.5–24.5),2159	39.4 (35.7–43.4),3299	45.9 (42.4–49.4),2867	38.9 (37.0–40.9),2317
**Education:** No education	22.1 (20.0–24.3),1307	14.5 (13.2–15.9),747	11.5 (10.7–12.4),953	17.8 (16.3–19.5),1816	8.5 (7.2–9.9),545	1.8 (1.4–2.3),135
Primary	38.7 (36.7–40.7),2210	66.2 (64.4–68.0),3672	68.1 (66.4–69.7),5666	65.8 (64.2–67.4),5931	51.8 (49.5–54.1),3329	27.6 (26.1–29.2),2044
Secondary	21.1 (19.6–22.7),1230	16.7 (15.3–18.3),1351	15.8 (14.7–16.9),1434	14.7 (13.5–15.9),1297	30.3 (28.4 - 32.3),1953	62.8 (61.1–64.4),4146
Higher	18.1 (16.3–20.0),1122	2.5 (2.1–3.0),288	4.6 (3.9–5.5),510	1.7 (1.3–2.1),137	9.4 (8.0 - 11.1),583	7.8 (6.5–9.2),455
**Wealth quintiles:**						
1, lowest	14.6 (12.5–17.0),910	17.3 (15.7–19.1),748	20.4 (18.3–22.7),1600	18.3 (16.1–20.8),1821	16.3 (14.6–18.1),1055	18.9 (17.3–20.6),1530
2	16.1 (14.5–17.8),972	18.5 (17.0–20.1),863	19.2 (17.5–21.0),1542	19.6 (18.0–21.4),1807	17.5 (16.1–19.0),1140	18.4 (17.2–19.6),1318
3	19.1 (17.6–20.6),1144	19.7 (18.3–21.2),957	19.5 (18.1–21.0),1580	20.0 (18.3–21.8),2063	19.7 (17.9–21.7),1258	17.5 (16.0–19.1),1180
4	23.1 (21.3–25.0),1356	20.7 (19.3–22.2),1207	20.2 (18.7–21.9),1662	20.5 (18.6–22.5),1798	22.2 (20–24.6),1424	21.4 (19.1–23.8),1282
5, highest	27.1 (24.5–29.9),1500	23.7 (21.6–25.9),2287	20.6 (18.1–23.3),2182	21.6 (19.4–24.0),1694	24.3 (21.7–27.1),1506	23.9 (21.4–26.5),1474
**N**	5882	6062	8571	9186	6413	6784

Self-reported cervical pre-cancer screening, proportions in percentages, Pearson's test with jack-knife 95% confidence intervals, N is number of observations. Ethiopia dataset had an urban variable which was divided into population sizes <50,000 and >50,000. We classified a population size of <50,000 as rural and >50,000 as urban. ‡ Estimate based on 25–49 observations and should be interpreted with caution.

For peer review only - http://bmjopen.bmj.com/site/about/guidelines.xhtml

Figure 1 shows the proportion of respondents reporting screening for cervical pre-cancer by age group (25-34 versus 35 – 49-years), residence (rural versus urban), education level, and wealth quintile by country and Table 2 presents adjusted odds ratios. In all countries except Zambia, more 35–49-year-olds than 25–34-year-olds reported screening for cervical pre-cancer (Figure 1). Age-related differences in reported screening varied and ranged in percentage points from 0.2% (1.7% among 25–34-year-olds versus 1.9% among 35 – 49-year-olds) in Rwanda to 2.8% (7.3% among 25–34-year-olds versus 10.1% among 35 – 49-year-olds) in Zimbabwe. After adjusting for covariates, 35–49-year-olds in all countries surveyed reported being more likely to be screened for cervical pre-cancer than 25–34-year-olds (Table 2).

More urban residents than rural residents reported screening for cervical pre-cancer in Ethiopia, Tanzania, Zambia, and Zimbabwe before adjusting for covariates (Figure 1). In these countries, the rural-urban inequalities in cervical pre-cancer screening were low, except in Zambia (5.1%, moderate). After adjustment, more urban residents than rural residents in Ethiopia, Malawi, Rwanda, and Tanzania reported screening for cervical pre-cancer (Table 2).

Respondents in Malawi, Rwanda, Zambia, and Zimbabwe had education-related inequalities (Figure 1). Education-related inequalities were also low in these countries except Zambia, where it was moderate between the uneducated and those with secondary schooling (5.9%). Respondents in Malawi, Tanzania, Zambia, and Zimbabwe had significant wealth-related inequalities in cervical pre-cancer screening (Figure 1 and Table 2). In Malawi and Tanzania, the inequalities between respondents in the lowest and highest wealth quintiles were low – 4.9% and 1.9%, respectively. They were moderate in Zambia (5.7%) and Zimbabwe (5.3%) (Figure 1). The goodness-of-fit test showed that our models fit the data well (Table 2).

For peer review only - http://bmjopen.bmj.com/site/about/guidelines.xhtml

241 [Figure 1 here]

For peer review only

For peer review only - http://bmjopen.bmj.com/site/about/guidelines.xhtml

Table 2: Survey weighted differences in self-reported cervical pre-cancer screening among 25 – 49-year-olds by age-groups, rural-urban location, education, and wealth quintile by country – (odds ratios [95% CI]) (PHIA 2015–2019)

**Inequality dimensions**	**Ethiopia**	**Malawi**	**Rwanda**	**Tanzania**	**Zambia**	**Zimbabwe**
**Age** (years) 35–49 (ref 25–34)	1.90 (1.48–2.43)	1.77 (1.39–2.27)	1.46 (1.11–1.93)	2.32 (1.85–2.91)	1.50 (1.28–1.75)	2.31 (1.92–2.78)
**Residence** Urban (ref: rural)	1.65 (1.02–2.65)	1.91 (1.29 - 2.82)	1.67 (1.05–2.65)	1.57 (1.03–2.41)	1.27 (0.96–1.68)	0.90 (0.65–1.24)
**Education** Primary (ref: no education)	0.97 (0.60 –1.54)	1.04 (0.74–1.48)	1.84 (1.04–3.26)	0.91 (0.61–1.36)	1.45 (0.97–2.16)	0.78 (0.44–1.38)
Secondary	1.32 (0.77–2.29)	1.69 (1.11–2.58)	3.11 (1.69–5.71)	1.19 (0.70–2.00)	1.81 (1.19–2.75)	1.10 (0.63–1.94)
Higher	1.73 (1.02–2.93)	2.10 (1.28–3.44)	2.08 (0.99–4.38)	1.38 (0.71–2.67)	2.52 (1.61–3.94)	1.95 (1.05–3.60)
**Wealth quintiles**: Q2 (ref: lowest Q1)	0.92 (0.46–1.85)	1.42 (0.85–2.36)	1.00 (0.60–1.66)	1.39 (0.78–2.49)	0.98 (0.61–1.58)	1.38 (1.00–1.91)
Q3	1.63 (0.75–3.51)	1.57 (0.97–2.55)	1.32(0.81–2.15)	1.72 (1.07–2.77)	2.24 (1.51–3.34)	2.04 (1.47–2.85)
Q4	1.78 (0.80–3.97)	2.16 (1.32–3.53)	1.25 (0.77–2.04)	1.93 (1.08–3.44)	2.40 (1.54–3.74)	3.24 (2.19–4.82)
Q5 Highest Quintile	2.13 (0.96–4.75)	3.53 (2.15 5.80)	1.34 (0.74–2.43)	2.44 (1.31– 4.54)7	4.01(2.51– 6.40)	4.95 (3.17–7.70)
**Goodness of fit** Prob > F	0.4317	0.9611	0.9176	0.9082	0.9569	0.9791
	F (9,17) = 1.07	F (9,17) = 0.31	F (9,17) = 0.40	F (9,17) = 0.42	F (9,17) = 0.32	F (9,17) = 0.25
N	5850	6053	8553	9167	6,375	6,777

Pearson test with jack-knife 95% confidence intervals for cell proportions, N= number of observations. *Not significant after correcting for multiple hypothesis testing with the sharpened Qs. Significance was set at p-value = 0.05. Goodness of fit test (Prob>). Ethiopia data set only had an urban variable. We classified an urban population size <50,000 as rural and >50,000 as urban· The regressions also controlled for the dummy variables province for Rwanda, Zambia, and Zimbabwe; region for Ethiopia; zone for Malawi; and mainlandzanziba for Tanzania. The results are not included. Q 1 – Lowest wealth quintile, Q 2, Q 3, Q 4, Q 5 – Highest wealth quintile.

For peer review only

Figure 2 shows the probabilities of self-reported cervical pre-cancer screening for selected combinations of age group (25–34, 35 – 49–years), residence (rural, urban), education level, and wealth quintile in percentage points by country. There were significant differences in cervical screening probabilities between the rural residents in the lowest wealth quintile with no education aged 25 – 34 years (25–34#Rural#Noed#1 in Figure 2) and their urban residents peers in the highest wealth quintile, highest educated (25–34#Urban#High#5) in Figure 2, ranging from 3.3% in Rwanda to 29.0% in Zambia. When similar comparisons were made for 35–49-year-olds, the differences ranged from 3.6% in Rwanda to 38.8% in Zimbabwe. A comparison of 25–34–years–old rural residents, with primary education in the second wealth quintile (25–34#Rural#Prim#2) and 35–49–year-old urban residents, with the highest education in the highest wealth quintile (35–49#Urban#High#5), showed substantially increased differences, ranging from 4.4% in Rwanda to 44.6% in Zimbabwe. Inequalities between 25–34–years–old urban residents, with the highest education, in the highest wealth quintile (25-34#Urban#High#5) and 35–49–year–old urban residents, with the highest education, in the highest wealth quintile (35–49#Urban#High#5) were moderate in magnitude, except in Rwanda where they were low (Figure 2).

[Figure 2 here]

Discussion

This study examined cervical pre-cancer screening inequalities related to age, residence, education, and wealth in six sub-Saharan African countries – Ethiopia, Malawi, Rwanda, Tanzania, Zambia, and Zimbabwe. We found that self-reported cervical pre-cancer was low and inequitable. None of the countries reached a third of 70% WHO cervical pre-cancer screening target, eight years away from the 2030 deadline. Self-reported cervical pre-cancer screening rates ranged from 3.5% (95% CI 3.1%–4.0%) in Rwanda to 17.1% (95% CI

For peer review only - http://bmjopen.bmj.com/site/about/guidelines.xhtml

15.8%–18.5%) Zambia and Zimbabwe (17.4% (CI16.1%–18.8%). However, older women, residing in urban areas, who were more educated and in the highest wealth quintile reported a higher uptake of cervical pre-cancer screening than younger women, living in rural areas, who were uneducated and in the lowest wealth quintile. Age-related inequalities were the most common – observed in all six surveyed countries. Rural-urban, education, and wealth-related inequalities were each observed in four countries, ranging from low to moderate in magnitude. We also observed high inequalities from combinations of inequalities in age group, residence (rural, urban), education level, and wealth in a few countries. These results have policy implications for the rapid scale-up of equitable life-saving cervical pre-cancer screening.

In this study, self-reported cervical pre-cancer screening was lower than a third of the WHO target of screening 70% of eligible women for cervical pre-cancer by 2030, consistent with other studies conducted in the surveyed countries. Studies found that cervical pre-cancer screening uptake ranged from 11% in Tanzania to 26.5% in Malawi, despite differences in methodologies used to estimate cervical pre-cancer screening (9,10). Countries should urgently scale up cervical pre-cancer screening to meet the WHO cervical pre-cancer screening target, removing inequalities that prevent some women from accessing screening for cervical pre-cancer, and integrate cervical pre-cancer screening in maternal, HIV, sexual and reproductive health, and social protection programmes.

Evidence supports the finding that inequalities in these underserved cervical pre-cancer screening countries favoured women who were older, resided in urban areas, were more educated and in the highest wealth quintile than younger women, who lived in rural areas, were uneducated and in the lowest wealth quintile (9,17,18). Studies conducted in Ethiopia, Rwanda, Tanzania, Zambia, and Zimbabwe, also support this finding (10,11,12,13,14,15,34,35,36). A study of 18 resource-constrained countries, of which eight

For peer review only - http://bmjopen.bmj.com/site/about/guidelines.xhtml

were from sub-Saharan Africa further found that wealth status increased socioeconomic inequalities in cervical pre-cancer screening, whereas being married, unemployed and living in urban communities reduced it (9). The results of this study agree with existing evidence on socioeconomic inequalities in maternal health. A study in Malawi found that rural-urban residence, education, and wealth status significantly impacted the uptake of three types of maternal health services in the study population. It found that compared with women residing in rural areas, who had lower education and were in the poorest wealth quintile, women living in urban areas, who had higher education and were in the higher wealth quintiles had significantly higher odds of receiving four antenatal care visits, skilled birth attendance and postnatal care (37). A study from Zambia reported similar findings (38). So did a study from 36 sub-Saharan African countries, concluding that prioritizing quality education of women in sub-Saharan Africa would reduce disparities in antenatal service utilization (39). Countries should urgently scale-up cervical pre-cancer screening, addressing inequalities that prevent some women from accessing services.

This study also determined the magnitude of the socioeconomic inequalities in reported cervical pre-cancer screening to aid policymaking in the prioritization of inequalities to eliminate in the scale-up of cervical pre-cancer screening. We found low to moderate age, rural-urban, education, and wealth-related inequalities in cervical pre-cancer screening in the surveyed countries. Age-related inequalities were reported in all the surveyed countries and residence, education, and wealth-related inequalities in four of the six surveyed countries. Although the inequalities were low to moderate, our study found that their combined effects on cervical pre-cancer screening probabilities were substantial, especially in countries with higher rates of self-reported cervical pre-cancer screening. The inequalities in cervical pre-cancer screening rates between 25 – 34-year-old rural residents, with no education in the lowest wealth quintile, and their urban resident peers, with highest education in the highest

For peer review only

wealth quintile, ranged from low inequalities in Rwanda, to moderate inequalities in Ethiopia and Tanzania to high inequalities in Malawi, Zambia, and Zimbabwe. Comparing primary educated 25–34–years–old rural residents, in the lowest wealth quintile with the highest educated 35–49–year–old urban residents, in the highest wealth quintile showed substantial differences. Inequalities did not disappear between the 25–34–years–old most affluent, highest educated, urban residents, and 35–49–year–old, most affluent, highest educated, urban residents. These results suggest that inequalities persist. Minor or moderate inequalities compound into high inequalities, further leaving women behind in cervical pre-cancer screening. Starting with age-related inequalities, eliminating socioeconomic inequalities should be prioritized in the urgent scale-up of cervical pre-cancer screening interventions.

Comparing the proportion of HIV–positive women in the sample and their reported cervical pre-cancer screening showed cervical pre-cancer screening gaps among HIV–positive women. In our study, the proportion of HIV positive 25–49-year-olds ranged from 4.7% (95% CI 4.1%–5.4%) 4.7 in Rwanda to 22.6% (95% CI 21.6%–23.7%) in Zimbabwe. By contrast, self-reported cervical pre-cancer screening among this population ranged from 0.4% (95% CI 0.3%–0.5%) in Rwanda to 5.4% (95% CI 4.7%–6.1%) in Zambia. Not all HIV–positive women were screened for cervical pre-cancer for several reasons. One reason may be the perception that linking women testing HIV positive to HIV treatment eliminates the risk of cervical pre-cancer for such women. A study in Zimbabwe found that many women living with HIV on ART screened positive for cervical pre-cancer, despite the country attaining HIV treatment targets (40). Another reason women may have not been screened for cervical pre-cancer is that they may have tested HIV positive at a facility with a limited capacity to conduct cervical pre-cancer screening. Alternatively, women might have been unable to access health facilities with the capacity to test for cervical pre-cancer for various reasons, including transport costs. A large proportion of HIV-negative women who were tested for

For peer review only - http://bmjopen.bmj.com/site/about/guidelines.xhtml

For peer review only

HIV in this study were not screened for cervical pre-cancer. One reason is the perception that HIV–negative women may be at low risk of cervical pre-cancer (14). Integration between HIV testing and cervical pre-cancer screening is required and is feasible in the scale up of cervical pre-cancer screening (41). Increased resources for HIV testing, training, and orientation of health care staff to screen eligible women for cervical pre-cancer, including HIV–negative women, is required to scale up screening services and reduce inequalities in the uptake of services.

Besides intensifying cervical pre-cancer screening in HIV programmes, governments should fulfil their obligations to the right to sexual and reproductive health by increasing the accessibility of their health systems (8). They should re-orient the building blocks of health systems – governance, financing, human resources, information, medical technologies, and service delivery– towards addressing inequalities and reaching all eligible women (8,42). They should establish, fund and scale-up population-level public-sector screening and treatment services and integrate them into HIV, STI, maternal and reproductive health services (43). Governments could build cervical pre-cancer screening around a target population, such as eligible schoolgirls and colleague students, which would assist in addressing socioeconomic inequalities. For example, Rwanda vaccinated more than 90% of schoolgirls against HPV from 2011 to 2012, through designated "health days" which were adapted for other sexual reproductive and maternal health purposes (44,45). This approach reduced time, money, and other costs of accessing HPV vaccination for girls, and other maternal health services for women (44). In contrast to other countries surveyed in this study, none of the wealth levels were associated with cervical pre-cancer screening in Rwanda. However, the cervical pre-cancer screening rate in Rwanda in this study was low at 3.5% (3.1%–4.0%), illustrating that cervical pre-cancer screening did not scale-up as did the HPV vaccination (45). Additional activities may be added to such programmes to increase access

For peer review only - http://bmjopen.bmj.com/site/about/guidelines.xhtml

and reduce inequalities. These include multiple screening over the lifetime, reducing the perception of high economic costs associated with cervical pre-cancer screening, and instituting same-day cervical pre-cancer screening and treatment (46). Other recommendations are the removal of economic barriers and focused outreach to women less likely to be screened and allowing them to self-collect samples (1,15,46). Women's ability to benefit from these programmes may depend on their social health protection, such as whether they have childcare responsibilities that prevent them from accessing services (21). Women with multiple inequalities depend on social protection and may be vulnerable under systems with low social protection (21). Governments should expand their social protection coverage and enhance their health system accessibility to scale up cervical pre-cancer screening, addressing the socioeconomic inequalities that prevent women from accessing services (21).

This study has several limitations. Collinearity was present, especially between the wealth and residence variables which tend to correlate with each other, consistent with how the wealth variable is constructed. Recall bias was present as cervical pre-cancer screening is self-reported. We did not control for the type of cervical pre-cancer screening because we did not have information on the types of screening women received. Cervical pre-cancer screening uptake may have worsened because of the COVID-19 pandemic. We did not determine if women had received two screenings by age 45 years as recommended by WHO, which could change our results. However, we used high-quality population-based data providing useful study findings to countries in sub-Saharan Africa.

Cervical pre-cancer screening in the surveyed countries was inequitable and low – less than a third of the WHO target to screen 70% of eligible women by 2030 for cervical pre-cancerous lesions. Women who were younger, lived in rural areas, had no education and were from poor backgrounds were left behind in cervical pre-cancer screening. Age-related inequalities were the most common. In combination, inequalities related to age, residence, education, and

For peer review only - http://bmjopen.bmj.com/site/about/guidelines.xhtml

wealth left younger, rural, uneducated, and poor women behind further. Focused and inclusive national cervical pre-cancer interventions are required to scale-up equitable cervical pre-cancer screening. Governments should include and monitor equity in their cervical pre-cancer screening programmes and research is needed to understand how to scale-up equitable cervical pre-cancer screening in sub-Saharan Africa countries.

a. Contributorship statement

DC designed the study. DC and SA collated the data. DC, SA, SK, ALNM, MP, JB, JE, and OK performed the data analysis. DC conducted the data visualisation, and drafted, and revised the manuscript. SA, SK, ALNM, MP, JB, JE, and OK critically reviewed the drafts. All authors approved the final manuscript.

b. Competing interests

The authors declare no competing interests.

c. Funding statement

This manuscript was supported by Swiss National Science Foundation (SNSF) grant number 202660.

d. Data sharing statement

Detailed descriptions of the PHIA are available at https://phia-data.icap.columbia.edu/files.

e. Ethics statement

The study did not require ethical clearance. The data are publicly available and de-identified at https://phia-data.icap.columbia.edu/files.

f. Acknowledgements

We acknowledge the support from the SNSF.

For peer review only - http://bmjopen.bmj.com/site/about/guidelines.xhtml

430 # References

431

1. World Health Organization. Global strategy to accelerate the elimination of cervical cancer as a public health problem. Geneva: World Health Organization; 2020. Report No.: ISBN 978-92-4-001410-7.

2. World Health Organization. WHO guideline for screening and treatment of cervical pre-cancer lesions for cervical cancer prevention, second edition. Geneva: World Health Organization; 2021. Report No.: https://apps.who.int/iris/handle/10665/336583.

3. Rohner E, Bütikofer L, Schmidlin K, Sengayi M, Maskew M, Giddy J, et al. Cervical cancer risk in women living with HIV across four continents: A multicohort study. Int J Cancer. 2020 Feb 1; 146(3; doi: 10.1002/ijc.32260): 601-609.

4. Campos NG, Sharma M, Clark A, Lee K, Geng F, Regan C, et al. The health and economic impact of scaling cervical cancer prevention in 50 low- and lower-middle-income countries. Int J Gynaecol Obstet. 2017 Jul; Suppl 1(138 doi: 10.1002/ijgo.12184): 47-56.

5. World health Organizaation (WHO). Health Equity. [Online].; 2021 [cited 2021 November 10. Available from: https://www.who.int/health-topics/health-equity#tab=tab_1.

6. Emadi M, Delavari S, Bayati M. Global socioeconomic inequality in the burden of communicable and non-communicable diseases and injuries: an analysis on global burden of disease study 2019. BMC Public Health. 2021 September; 21(https://doi.org/10.1186/s12889-021-11793-7): 1771.

7. O'Donnell O, Doorslaer Ev, Wagstaff A, Lindelow M. Analyzing health equity using Household Survey Data. A guide to techniques and their Implementation Washington DC: The International Bank for Reconstruction and Development / The World Bank; 2008.

8. Perehudoff K, Vermandere H, Williams A, Bautista-Arredondo S, Paepe ED, Dias S, et al. Universal cervical cancer control through a right to health lens: refocusing national policy and programmes on underserved women. BMC Int Health Hum Rights. 2020 July; 20(21. doi.org/10.1186/s12914-020-00237-9).

9. Mahumud RA, Keramat SA, Ormsby GM, Sultana M, Rawal LB, Alam K, et al. Wealth-related inequalities of women's knowledge of cervical cancer screening and service utilisation in 18 resource-constrained countries: evidence from a pooled decomposition analysis. International Journal for Equity in Health. 2020 March; 19(42 doi.org/10.1186/s12939-020-01159-7).

10. Ayenew AA, Zewdu BF, Nigussie AA. Uptake of cervical cancer screening service and associated factors among age-eligible women in Ethiopia: systematic review and meta-analysis. Infectious Agents and Cancer. 2020 November; 15(doi.org/10.1186/s13027-020-00334-3): 67.

For peer review only

11. Henke A, Kluge U, Borde T, Mchome B, Serventi F, Henke O. Tanzanian women´s knowledge about Cervical Cancer and HPV and their prevalence of positive VIA cervical screening results. Data from a Prevention and Awareness Campaign in Northern Tanzania, 2017 – 2019. Glob Health Action. 2021 Jan; 14(1 doi: 10.1080/16549716.2020.1852780): 1852780.

12. Weng Q, Jiang J, Haji FM, Nondo LH, Zhou H. Women's knowledge of and attitudes toward cervical cancer and cervical cancer screening in Zanzibar, Tanzania: a cross-sectional study. BMC Cancer. 2020 January; 20(doi.org/10.1186/s12885-020-6528-x): 63.

13. Barrow A, Onikan A, Nzoputam CI, Ekholuenetale M. Prevalence and determinants of cervical cancer awareness among women of reproductive age: evidence from Benin and Zimbabwe population-based data. Appl Cancer Res. 2020 October; 40(doi.org/10.1186/s41241-020-00092-z).

14. Moucheraud C, Kawale P, Kafwafwa S, Bastani R, Hoffma RM. "It is big because it's ruining the lives of many people in Malawi": Women'sattitudes and beliefs about cervical cancer. Preventive Medicine Reports. 2020 June; 18(DOI: 10.1016/j.pmedr.2020.101093): 101093.

15. Pry JM, Manasyan A, Kapambwe S, Taghavi K, Duran-Frigola M, Mwanahamuntu M, et al. Cervical cancer screening outcomes in Zambia, 2010–19: a cohort study. The Lancet Global Health. 2021 June; 9(6 DOI:https://doi.org/10.1016/S2214-109X(21)00062-0): E832-E840.

16. Nyambe A, Kampen JK, Baboo SK, Hal GV. Knowledge, attitudes and practices of cervical cancer prevention among Zambian women and men. BMC Public Health 19, 508 (2019). https://doi.org/10.1186/s12889-019-6874-2. BMC Public Health. 2019 May; 19(508 doi.org/10.1186/s12889-019-6874-2).

17. Akanda R, Kawale P, Corrina M. Cervical cancer prevention in Africa: A policy analysis. Journal of Cancer Policy. 2022 January;(https://doi.org/10.1016/j.jcpo.2021.100321).

18. Akokuwebe ME, Idemudia ES, Lekulo AM, Motlogeloa OW. Determinants and levels of cervical Cancer screening uptake among women of reproductive age in South Africa: evidence from South Africa Demographic and health survey data, 2016. BMC Public Health. 2021 November; 21(1, doi.org/10.1186/s12889-021-12020-z): 2013.

19. Keetile M, Ndlovu K, Letamo G, Disang M, Yaya S, Navaneetham K. Factors associated with and socioeconomic inequalities in breast and cervical cancer screening among women aged 15–64 years in Botswana. PLoS ONE. 2021 August; 16(8 doi.org/10.1371/journal.pone.0255581): e0255581.

20. Quizhpe E, Sebastian MS, Teran E, Pulkki-Brännström AM. Socioeconomic inequalities in women's access to health care: has Ecuadorian health reform been successful? International Journal for Equity in Health. 2020 October; 19(doi.org/10.1186/s12939-020-01294-1): 178.

21. Prez VD, Jolidon V, Willems B, Cullati S, Burton-Jeangros C, Bracke P. Cervical cancer screening programs and their context-dependent effect on inequalities in screening

For peer review only - http://bmjopen.bmj.com/site/about/guidelines.xhtml

uptake: a dynamic interplay between public health policy and welfare state redistribution. Int J Equity Health. 20 September; 20(doi.org/10.1186/s12939-021-01548-6): 211.

22. Jolidon V, Prez VD, Willems B, Bracke P, Cullati S, Burton-Jeangros C. Never and under cervical cancer screening in Switzerland and Belgium: trends and inequalities. BMC Public Health. 2020 October; 20(doi.org/10.1186/s12889-020-09619-z): 1517.

23. Stelzle D, Tanaka LF, Lee KK, Khalil AI, Baussano I, Shah ASV, et al. Estimates of the global burden of cervical cancer associated with HIV. Lancet Glob Health. 2021 February; 9(2. doi.org/10.1016/ S2214-109X(20)30459-9): E161-E169.

[dataset]24. Ministry of Health, Ethiopian Public Health Institute, Federal HIV/AIDS Prevention and Control Office, Central Statistical Agency of Ethiopia, Ethiopian Public Health Association. Ethiopia Population-based HIV Impact Assessment 2017-2018 (EPHIA 2017-2018) (various datasets). Addis Ababa: MOH, PEPFAR, CDC, Westat, ICAP at Columbia University [Producers]. ICAP at Columbia University [Distributor]; 2021.

[dataset]25. Ministry of Health (MOH). Malawi Population-Based HIV Impact Assessment 2015-2016 (MPHIA 2015-2016) (various) [Datasets]. Lilongwe: MOH, National AIDS Commission, PEPFAR, CDC, Westat, ICAP at Columbia University [Producers]. ICAP at Columbia University [Distributor]; 2021.

[dataset]26. Government of Rwanda, Rwanda Biomedical Centre, National Institute of Statistics of Rwanda, University of Rwanda, World Health Organization, Rwanda United Nations Children's Fund (UNICEF). Rwanda Population-based HIV Impact Assessment 2018-2019 (RPHIA 2018-2019) (various datasets). Kigali: Government of Rwanda, PEPFAR, CDC, Westat, ICAP at Columbia University [Producers]. ICAP at Columbia University [Distributor]; 2021.

[dataset]27. Tanzania Commission for AIDS, Zanzibar AIDS Commission, Ministry of Health, Community Development, Gender, Elderly and Children, Mainland Tanzania, Zanzibar Integrated HIV, Hepatitis, Tuberculosis and Leprosy Program, Ministry of Health Zanzibar. Tanzania HIV Impact Survey 2016 - 2017 (THIS 2016-2017). Dar es Salaam: TACAIDS, PEPFAR, CDC, Westat, ICAP at Columbia University [Producers]. ICAP at Columbia University [Distributor]; 2021.

[dataset]28. Ministry of Health, Zambia National Public Health Institute, National HIV/AIDS/STI/TB Council Zambia, University of Zambia, Central Statistical Office Zambia, Tropical Disease Research Centre, University Teaching Hospital, Zambia. Zambia Population-based HIV Impact Assessment 2016 (ZAMPHIA 2016) (various datasets). Lusaka: MOH, National HIV/AIDS/STI/TB Council Zambia, PEPFAR, CDC, Westat, ICAP at Columbia University [Producers]. ICAP at Columbia University [Distributor]; 2021.

[dataset]29. Ministry of Health and Child Care Zimbabwe, Zimbabwe National Statistics Agency, Biomedical Research and Training Institute, Lancet Clinical, Zimbabwe Statistical Center for HIV/AIDS Research and Prevention. Zimbabwe Population-based HIV Impact Assessment 2015 - 2016 (ZIMPHIA 2015-2016). Harare: MoHCC, PEPFAR, CDC, Westat, ICAP at Columbia University [Producers]. ICAP at Columbia University [Distributor]; 2021.

For peer review only

30. World Health Organization. State of inequality: HIV, tuberculosis and malaria. Geneva: World Health Organization; 2021. Report No.: Licence: CC BY-NC-SA 3.0 IG.

31. Population-Based HIV Impact Assessment (PHIA) Data Use Manual. New York:; July 2019.

32. Population-Based HIV Impact Assessments (PHIA) Project. Population-Based HIV Impact Assessments (PHIA) Guiding the Global AIDS Response. [Online].; 2021 [cited 2021 February 22. Available from: https://phia-data.icap.columbia.edu/files.

33. Ogrinc G, Davies L, Goodman D, Batalden P, Davidoff F, Stevens D. SQUIRE 2.0 (Standards for QUality Improvement Reporting Excellence): revised publication guideliness from a detailed consensus process. BMJ Qual Saf. 2015.. 2015;(PMID: 26369893).

34. Niyonsenga G, Gishoma D, Sego R, Uwayezu MG, Nikuze B, Fitch M, et al. Knowledge, utilization and barriers of cervical cancer screening among women attending selected district hospitals in Kigali - Rwanda. Canadian Oncology Nursing Journal. 2021 July; 31(3. doi: 10.5737/23688076313266274): 266–274.

35. Ndateba I, Kabatsinda A, Ndabarora E. Uptake of Cervical Cancer Screening and Associated Factors Among Women Attending Outpatient Services in Rwamagana Hospital, Rwanda. Rwanda Journal of Medicine and Health Sciences. 2021 December; 4(3. doi: 10.4314/rjmhs.v4i3.8).

36. Kapambwe S, Sahasrabuddhe VV, Blevins M, Mwanahamuntu MH, Mudenda V, Shepherd BE, et al. Implementation and Operational Research: Age Distribution and Determinants of Invasive Cervical Cancer in a "Screen-and-Treat" Program Integrated With HIV/AIDS Care in Zambia. J Acquir Immune Defic Synd. 2015 Sep; 70(1. doi: 10.1097/QAI.0000000000000685): e20-6..

37. Yaya S, Bishwajit G, Shah V. Wealth, education and urban–rural and maternal healthcare service usage in Malawi. BMJ Global Health. 2016; 1(doi:10.1136/bmjgh-2016-000085): e000085.

38. He Z, Zhang C, Wang S, Yang X. Socioeconomic Determinants of Maternal HealthCare Utilisation in Zambia: 1997-2014. INQUIRY: The Journal of Health Care Organization, Provision, and Financing. 2021; S8(doi: 10.1177/00469580211067480): 1-14.

39. Obse AG, Ataguba JE. Explaining socioeconomic disparities and gaps in the use of antenatal care services in 36 countries in sub-Saharan Africa. Health Policy and Planning. 2021 June ; 36(5): 651–661.

40. Taghavi K, Mandiriri A, Shamu T, Rohner E, Bütikofer L, Asangbeh S, et al. Cervical Cancer Screening Cascade for women living with HIV: A cohort study from Zimbabwe. PLOS Glob Public Health. 2022 Feb; 2(2. https://doi.org/10.1371/journal.pgph.0000156): e0000156.

41. Pfaff C, Singano V, Akello H, Amberbir A, Berman J, Kwekwesa A, et al. Early experiences in integrating cervical cancer screening and treatment into HIV services in

For peer review only - http://bmjopen.bmj.com/site/about/guidelines.xhtml

Zomba Central Hospital, Malawi. Malawi Med J. 2018 Sep; 30(3: doi: 10.4314/mmj.v30i3.14.): 211-214.

42. Gravitt PE, Silver MI, Hussey HM, Arrossi S, Huchko M, Jeronimo J, et al. Achieving equity in cervical cancer screening in low- and middle-income countries (LMICs): Strengthening health systems using a systems thinking approach. Preventive Medicine. 2021 March; 44(https://doi.org/10.1016/j.ypmed.2020.106322): 106322.

43. Parham GP, MwanahamuntuV MH, Kapambwe S, Muwonge R, Bateman AC, Blevins M, et al. Population-level scale-up of cervical cancer prevention services in a low-resource setting: Development, implementation, and evaluation of the cervical cancer prevention program in Zambia. PLoS ONE. 2015; 10(4. doi.org/10.1371/journal.pone.0122169).

44. Binagwaho A, Ngabo F, Wagner CM, Gatera M, Nutt CT, Nsanzimana S. Lessons from the field. Bulletin of the World Health Organisation. 2013; 91(doi: http://dx.doi.org/10.2471/BLT.12.116087): 697–703.

45. Muhimpundu MA, Ngabo F, Sayinzoga F, Balinda JP, Rusine J, Harward S, et al. Screen, Notify, See, and Treat: Initial Results of Cervical Cancer Screening and Treatment in Rwanda. JCO Global Oncology. 2021; 7(DOI: 10.1200/GO.20.00147): 632-638.

46. Campos NG, VT, Jeronimo J, Mvundura M, Kim JJ. Evidence-based policy choices for efficient and equitable cervical cancer screening programs in low-resource settings. Cancer Medicine. 2017 May; 6(8. doi: 10.1002/cam4.1123): 2008–2014.

Figure 1. Survey weighted proportions of 25–49-year-olds reporting being screened for cervical pre-cancer by age-groups, rural versus urban residence, education, and wealth quintile by country (Percentage, 95% CI) (PHIA 2015–2019).

1=lowest wealth quintile, 2, 3, 4, 5= highest wealth quintile. 25-34# =25 – 34, 35-49#=35 – 49 years old. #Rural=rural. #Rich= rich. Noed = no education, Prim= primary, High=higher education.

Figure 2. Survey weighted differences in probabilities of self-reported cervical pre-cancer screening by combinations of age-group, rural-urban residence, no education, higher education, and lowest to highest wealth quintiles by country. Marginal effects (percentages, 95% CI) (PHIA 2015–2019).

For peer review only - http://bmjopen.bmj.com/site/about/guidelines.xhtml

Figure 1

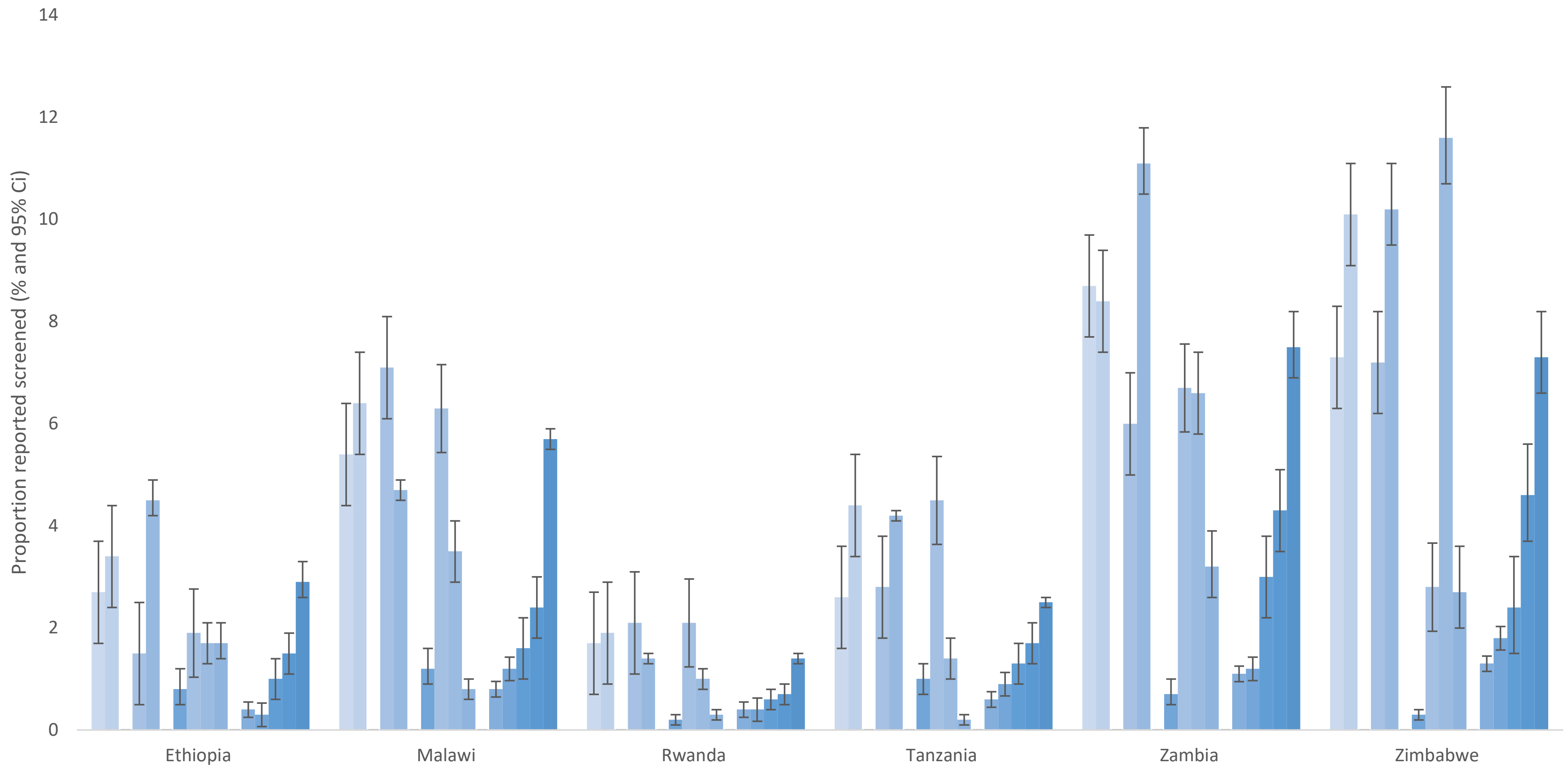
14
12
10
8
6
4
2
0
Proportion reported screened (% and 95% Ci)
Ethiopia
Malawi
Rwanda
Tanzania
Zambia
Zimbabwe

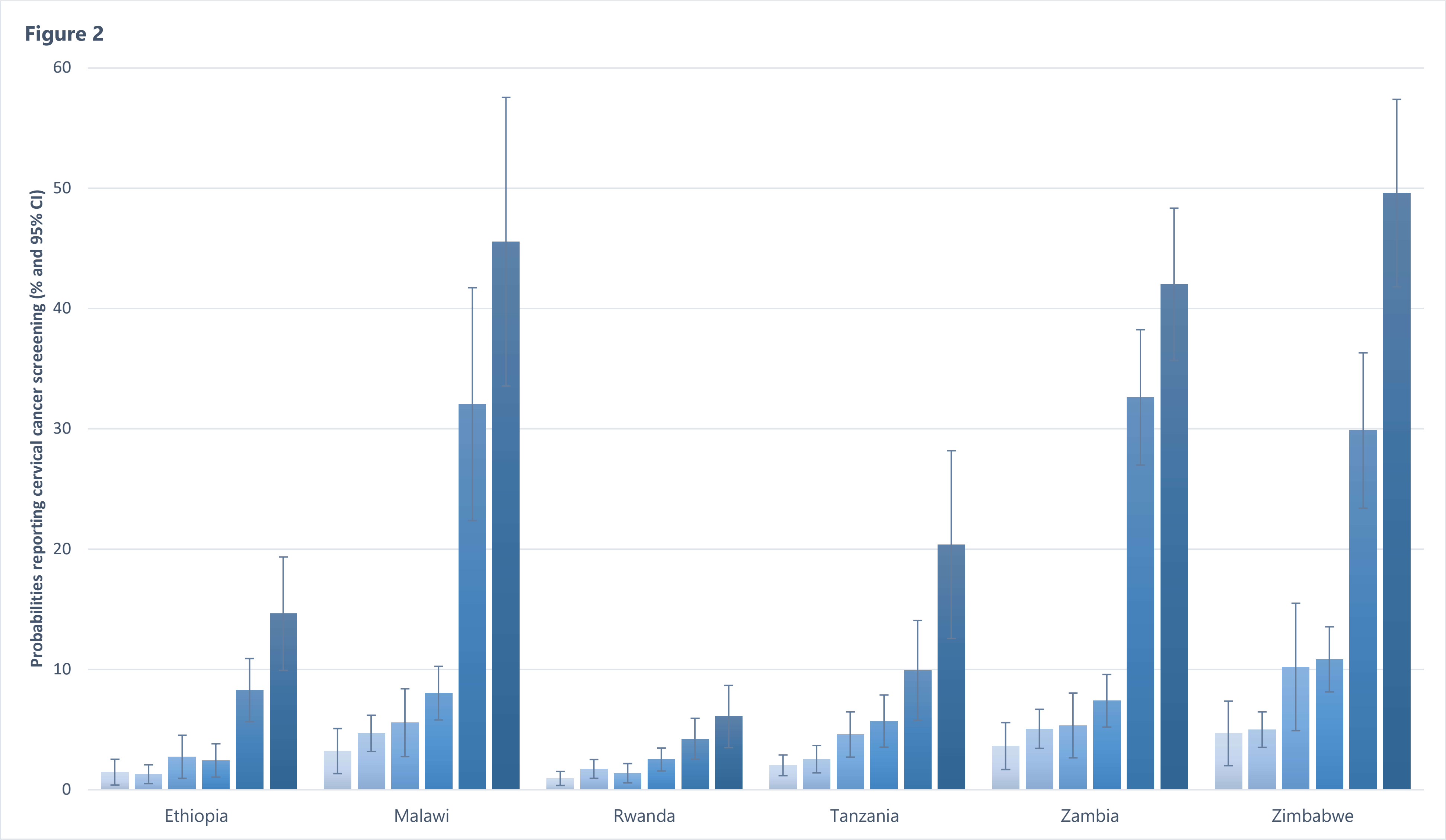
Figure 2
Probabilities reporting cervical cancer screeening (% and 95% CI)
60
50
40
30
20
10
0
Ethiopia
Malawi
Rwanda
Tanzania
Zambia
Zimbabwe

Supplementary Table 1. Variable descriptions.

Variable name	Description	Measure
Cervical pre-cancer screening	Self-reported from the adult interview question on ever testing for cervical pre-cancer, coded no, yes.	Binary
Sex	Self-reported sex (i.e., male, female).	Binary
Age	Self-reported linear years categorized in years (25–34, 35–49).	Categorical
Location, rural, urban	Self-reported residence (i.e., rural, urban), from the household interview. The data from Ethiopia had only urban variables. For Ethiopia, we coded size of area <=50,000 as rural = 0, and >50k =1; urban, from the household data set.	Binary
Ever married	Was derived from the question 'Have you ever been married or lived together with a (man/woman) as if married?' coded no, yes.	Binary
Ever tested for HIV	From the question 'Has been tested for HIV', coded never tested, ever tested.	Binary
HIV prevalence	The proportion of people 15 years or older testing HIV positive using laboratory-confirmed HIV test results from the adult biomarker questionnaire.	Binary
Educational status	Level of school respondent ever attended (i.e., not educated, primary school education, secondary school education, or higher education, from the adult interview. Tanzania's educations variables were standardized as follows: No education; Primary combined pre-primary, primary and post-primary; Secondary combined secondary, post-secondary training, secondary A); More than secondary (included post-secondary A levels and university).	Categorical
Wealth	Assessed household wealth with the wealth quintiles variable, which was derived from a wealth index. Wealth quintiles ranked households depending on their household characteristics and asset ownership from Q1 representing the poorest households to Q5 representing the wealthiest. (i.e., poorest, poor, rich, richer, and richest). The variable is provided in the PHIA household data set.	Ordered ranking
Regions, province, or zone	Defined as a political area, which is the primary sampling unit. It was province for Rwanda, Zambia, and Zimbabwe; region for Ethiopia, zone for Malawi, and mainlandzanziba for Tanzania.	Categorical

Note: Unless indicated the variables were from the adult interview data set. The answer options 'didn't know' or 'refused to answer' were coded as missing for all variables.

For peer review only - http://bmjopen.bmj.com/site/about/guidelines.xhtml

Reporting checklist for quality improvement in health care.

Based on the SQUIRE guidelines.

Instructions to authors

Complete this checklist by entering the page numbers from your manuscript where readers will find each of the items listed below.

Your article may not currently address all the items on the checklist. Please modify your text to include the missing information. If you are certain that an item does not apply, please write "n/a" and provide a short explanation.

Upload your completed checklist as an extra file when you submit to a journal.

In your methods section, say that you used the SQUIREreporting guidelines, and cite them as:

Ogrinc G, Davies L, Goodman D, Batalden P, Davidoff F, Stevens D. SQUIRE 2.0 (Standards for QUality Improvement Reporting Excellence): revised publication guidelines from a detailed consensus process

		Reporting Item	Page Number
**Title**			
	#1	Indicate that the manuscript concerns an initiative to improve healthcare (broadly defined to include the quality, safety,	1

For peer review only

		effectiveness, patientcenteredness, timeliness, cost, efficiency, and equity of healthcare)	
**Abstract**			
	#02a	Provide adequate information to aid in searching and indexing	2
	#02b	Summarize all key information from various sections of the text using the abstract format of the intended publication or a structured summary such as: background, local problem, methods, interventions, results, conclusions	2
**Introduction**			
Problem description	#3	Nature and significance of the local problem	3
Available knowledge	#4	Summary of what is currently known about the problem, including relevant previous studies	3
Rationale	#5	Informal or formal frameworks, models, concepts, and / or theories used to explain the problem, any reasons or assumptions that were used to develop the intervention(s), and reasons why the intervention(s) was expected to work	3
Specific aims	#6	Purpose of the project and of this report	4
**Methods**			
Context	#7	Contextual elements considered important at the outset of introducing the intervention(s)	5

For peer review only

Intervention(s)	#08a	Description of the intervention(s) in sufficient detail that others could reproduce it	N/A
Intervention(s)	#08b	Specifics of the team involved in the work	N/A
Study of the Intervention(s)	#09a	Approach chosen for assessing the impact of the intervention(s)	5
Study of the Intervention(s)	#09b	Approach used to establish whether the observed outcomes were due to the intervention(s)	6
Measures	#10a	Measures chosen for studying processes and outcomes of the intervention(s), including rationale for choosing them, their operational definitions, and their validity and reliability	6
Measures	#10b	Description of the approach to the ongoing assessment of contextual elements that contributed to the success, failure, efficiency, and cost	N/A
Measures	#10c	Methods employed for assessing completeness and accuracy of data	6
Analysis	#11a	Qualitative and quantitative methods used to draw inferences from the data	6
Analysis	#11b	Methods for understanding variation within the data, including the effects of time as a variable	N/A
Ethical considerations	#12	Ethical aspects of implementing and studying the intervention(s) and how they were addressed, including, but	20

For peer review only

		not limited to, formal ethics review and potential conflict(s) of interest	
**Results**			
	#13a	Initial steps of the intervention(s) and their evolution over time (e.g., time-line diagram, flow chart, or table), including modifications made to the intervention during the project	N/A
	#13b	Details of the process measures and outcome	6-9
	#13c	Contextual elements that interacted with the intervention(s)	6-9
	#13d	Observed associations between outcomes, interventions, and relevant contextual elements	6-9
	#13e	Unintended consequences such as unexpected benefits, problems, failures, or costs associated with the intervention(s).	N/A
	#13f	Details about missing data	6
**Discussion**			
Summary	#14a	Key findings, including relevance to the rationale and specific aims	9
Summary	#14b	Particular strengths of the project	14
Interpretation	#15a	Nature of the association between the intervention(s) and the outcomes	N/A
Interpretation	#15b	Comparison of results with findings from other publications	10-11

For peer review only

Interpretation	#15c	Impact of the project on people and systems	N/A
Interpretation	#15d	Reasons for any differences between observed and anticipated outcomes, including the influence of context	12 - 14
Interpretation	#15e	Costs and strategic trade-offs, including opportunity costs	N/A
Limitations	#16a	Limits to the generalizability of the work	14
Limitations	#16b	Factors that might have limited internal validity such as confounding, bias, or imprecision in the design, methods, measurement, or analysis	14
Limitations	#16c	Efforts made to minimize and adjust for limitations	14
Conclusion	#17a	Usefulness of the work	14
Conclusion	#17b	Sustainability	N/A
Conclusion	#17c	Potential for spread to other contexts	14
Conclusion	#17d	Implications for practice and for further study in the field	14
Conclusion	#17e	Suggested next steps	14
**Other information**			
Funding	#18	Sources of funding that supported this work. Role, if any, of the funding organization in the design, implementation, interpretation, and reporting	2

For peer review only

None The SQUIRE 2.0 checklist is distributed under the terms of the Creative Commons Attribution License CC BY-NC 4.0. This checklist can be completed online using https://www.goodreports.org/, a tool made by the EQUATOR Network in collaboration with Penelope.ai

For peer review only
