# Supplementary material for: Socioeconomic inequalities in cervical precancer screening among women in Ethiopia, Malawi, Rwanda, Tanzania, Zambia and Zimbabwe: analysis of Population-Based HIV Impact Assessment surveys
Source: BMJ Open. 2023 Jun 20;13(6):e067948. doi: 10.1136/bmjopen-2022-067948 (PMC10314495; doi:10.1136/bmjopen-2022-067948)
Supplement: Reviewer comments [file bmjopen-2022-067948.reviewer_comments.pdf]

PEER REVIEW HISTORY

BMJ Open publishes all reviews undertaken for accepted manuscripts. Reviewers are asked to complete a checklist review form (**http://bmjopen.bmj.com/site/about/resources/checklist.pdf**) and are provided with free text boxes to elaborate on their assessment. These free text comments are reproduced below.

ARTICLE DETAILS

**TITLE (PROVISIONAL)**	Socioeconomic inequalities in cervical pre-cancer screening among women in Ethiopia, Malawi, Rwanda, Tanzania, Zambia, and Zimbabwe – Analysis of Population-Based HIV Impact Assessment surveys.
**AUTHORS**	Chipanta, David; Kapambwe, Sharon; Nyondo-Mipando, Alinane Linda; Pascoe, Margaret; Amo-Agyei, Silas; Bohlius, Julia; Estill, Janne; Keiser, Olivia

VERSION 1 – REVIEW

**REVIEWER**	Habtu, Yitagesu<br>Addis Ababa University, School of Public health
**REVIEW RETURNED**	16-Sep-2022

**GENERAL COMMENTS**	Title: “Socioeconomic inequalities in cervical pre-cancer screening among women in Ethiopia, Malawi, Rwanda, Tanzania, Zambia, and Zimbabwe – Analysis of Population-Based HIV Impact Assessment surveys”<br>I would like to thank the editor for inviting me to review the manuscript. The article examined the association between selected socioeconomic variables and cervical pre-cancer screening for women. The paper may provide additional insights and benefit the policy makers and scientific community. However, I believe the authors are required to address few comments and /or otherwise clarified more to improve the manuscript.<br>Abstract<br>• Would you please make the sentence “Inequalities in cervical pre-cancer screening were low based on covariates” clear? (line 68). The same would apply to “Combining the inequalities led to significant inequalities in self-reported cervical pre-cancer screening” (line 70).<br>Methods<br>• “This is a cross-sectional study of a secondary data analysis of available PHIA data for countries that included cervical pre-cancer screening variables.” The data are extracted from different cross-sectional studies (the PHIA surveys) or at different time points; Ethiopia (2017–2018), Malawi (2015–2016), Rwanda (2018), Tanzania (2016–2017), Zambia (2016) and Zimbabwe (2015–2016). The point here is how could significant inequalities in cervical pre-cancer screening rates (different time points) favour older women, women who resided in urban areas, and more educated and wealthy women? Can we reach on our conclusion from “cervical pre-cancer screening rates” at different time points? Availability and accessibility of screening services?<br>• “…to evaluate HIV the impact of HIV programs in countries supported by United States President's Emergency Plan for AIDS Relief (PEPFAR)” (line 150)

	• I am not sure whether "Patient and Public Involvement" is placed in the appropriate section. Is that the journal's requirement?<br>• …"primary outcome" …(line 169), "primary", "secondary" apply to intervention studies as we have two types of variables there but, here, it would have been mentioned simply as "outcome variable"<br>• What was the justification for coding Ethiopia's data set as rural if the population size was smaller than 50,000 people? Would you please mention the source for this categorization?<br>• Regarding age, once the WHO created a deliberate inequalities in age (25-49) and have been providing the pre-cervical cancer screening, what is the need to estimate of the inequalities among the already established age categories? What was unknown about this since it was an established fact, hence WHO took measures on this specific issue?<br>• Do you think logistic regression demonstrates inequality among subgroups of the population with regard to pre-cervical cancer screening services?<br>• "In step 3,...each inequality alone and combined with other inequalities,…" (Lines, 187-188) Where is the combined one? Is it for the multiple logistic regression? How you analysed the differences using multivariable logistics regression after determining the associations between screening for cervical pre-cancer and each dimension of inequality? What is a common denominator to compare those inequalities than simply performing separate logistic regression for each dataset of each country?<br>• How you did the sharpened Qs and why?

**REVIEWER**	Ayanto, Samuel Yohannes<br>Jimma University
**REVIEW RETURNED**	27-Sep-2022

**GENERAL COMMENTS**	Please leave your comments for the authors below:<br>The research work addresses a very important public health problem and guides policy makers with its important findings. There are only few comments that would help the authors to revise their work before the manuscript can be considered for publication.<br>1. In the main outcomes section of the abstract you indicated the two potential outcomes of your study. In addition you have defined the way how you used the outcome variables which should actually be described under "operational definition" section or under "variables and outcome descriptions" section in your case where you actually described it. Therefore, it is better to remove it from the outcome section of the abstract.<br>2. In the conclusion under the abstract it says "None of the countries achieved one-third of the World Health Organisation target of screening 70% of eligible women for cervical pre-cancer by 2030." When is that achievement? Is it during your study period? Specify!<br>3. Unless the journal's requirements for manuscript preparation dictate otherwise, I would prefer the limitation section to be rewritten in the paragraph style than bullet format. Also you indicated recall bias as a limitation of the study as it was actually happened. Of course, it might have happened and hampered your findings negatively but still you cannot be 100% sure of it. Therefore, you can put the possibility of the introduction of recall bias in to your study, but you cannot be sure of it as if it was actually there.<br>4. On page 5 line 150-152, check for the grammatical correctness of the statement "The PHIA surveys collected a range of health and socio-demographic data to evaluate HIV the impact of HIV

	programs in countries supported by United States President's Emergency Plan for AIDS Relief (PEPFAR).”<br>5. On page 5 I recognized two methodological approaches. The first one indicates that you used secondary data analysis. The second one demonstrates the collection of primary data from household members. Was your research a mix of primary and secondary data collection and management approaches? I think this needs additional clarification of the methods in that for which variables you used secondary data and for which you used primary data.<br>6. On page 5-6 line168-169, you declared that “these dimensions of inequalities are familiar sources of disadvantage or discrimination.” If so, why did you repeat such investigation, despite the establishment of such well-known scientific facts before? It is also what your findings have confirmed. Would you justify this adequately!<br>7. On page 7 line 208-210 it says “More than half of the respondents resided in rural areas, excluding Ethiopia, where all respondents resided in urban areas, 54% of them in areas with <50,000 residents.” But on page 6 line 174-175 it says “Ethiopia’s data set had an urban variable, which we coded as rural if the population size was smaller than 50,000 people, and urban otherwise.” Also on page 9 line 226-227 it says “More urban residents than rural residents reported screening for cervical pre-cancer in Ethiopia, Tanzania, Zambia, and Zimbabwe before adjusting for covariates (Figure 1).” Are they all in alignment? If so try to simplify the statements!<br>8. Page 12 line 267-268 says “None of the countries reached a third of 70% WHO cervical pre-cancer screening target, eight years away from the 2030 deadline.” Is the eight years interval the same for all countries since the survey periods you used for each country were different?<br>9. Your discussion is majorly a repetition of the study findings in your results section. You have also demonstrated inadequate efforts to make comparisons of your findings with what is already there in the world of scientific literature. Also, your recommendations following the discussions usually used the obligatory word “should” which can preferably be expressed in the other way round.<br>10. There existed a colinearity problem in the analysis. But you did not mention any measure you have taken as a solution in your manuscript about the observed multicolinearity effect between wealth and residence.<br>11. You used very long paragraphs in your manuscript which seem uninteresting and difficult to comprehend its contents easily.<br>12. Some acronyms like PHIA and the like have not been addressed fully.

VERSION 1 – AUTHOR RESPONSE

Reviewer comments:

Comments	Response	What we changed to the manuscript and on which pages
Reviewer: 1<br>Dr. Yitagesu Habtu, Addis Ababa University, Hossana College of Health Sciences		
Comments to the Author:<br>Title: "Socioeconomic inequalities in cervical pre-cancer screening among women in Ethiopia, Malawi, Rwanda, Tanzania, Zambia, and Zimbabwe – Analysis of Population-Based HIV Impact Assessment surveys"<br>I would like to thank the editor for inviting me to review the manuscript. The article examined the association between selected socioeconomic variables and cervical pre-cancer screening for women. The paper may provide additional insights and benefit the policy makers and scientific community. However,<br>I believe the authors are required to address few comments and /or otherwise clarified more to improve the manuscript.	Many thanks Dr Habtu for your time and insightful comments which have substantially helped to improve the manuscript.	We have made specific changes to the manuscript responding to your questions as highlighted in the manuscript.
Abstract<br>• Would you please make the sentence "Inequalities in cervical pre-cancer screening were low based on covariates" clear? (line 68). The same would apply to "Combining the inequalities led to	Thank you for suggestion. We have made the change in the background and on line 68 and 70.<br><br>The covariates include the predictors which are age, residence, residence, education and wealth. We have specified the covariates by indicating predictors.	We write:<br><br>"This study examined inequalities in cervical pre-cancer screening within and between age-groups, residence, education and

significant inequalities in self-reported cervical pre-cancer screening” (line 70).		wealth of women in Ethiopia, Malawi, Rwanda, Tanzania…”<br><br>And<br><br>“Inequalities in cervical pre-cancer screening within and between sub-groups of women were low”<br><br>“Combining the inequalities led to significant inequalities between sub-groups of women”
Methods<br>• “This is a cross-sectional study of a secondary data analysis of available PHIA data for countries that included cervical pre-cancer screening variables.” The data are extracted from different cross-sectional studies (the PHIA surveys) or at different time points; Ethiopia (2017–2018), Malawi (2015–2016), Rwanda (2018), Tanzania (2016–2017), Zambia (2016) and Zimbabwe (2015–2016). The point here is how could significant inequalities in cervical pre-cancer screening rates (different time points) favour older women, women who resided in urban areas, and more educated and wealthy women? Can we reach on our conclusion from “cervical pre-cancer screening rates” at different time points? Availability and accessibility of screening services?	Thank you for your comments. We agree that the timelines and context are different by country and that this is a limitation of the study.<br><br>However, because the PHIA are standardized we can compare performance in pre-cancer screening across countries and variables in general.<br><br>We added as a limitation that the timelines and context differ. At the point of the analysis none of the countries had met a third of the WHO cervical cancer screening target	We write<br><br>The data were collected at different time points; Ethiopia (2017–2018), Malawi (2015–2016), Rwanda (2018), Tanzania (2016–2017), Zambia (2016) and Zimbabwe (2015–2016), and their milestones for reaching the WHO target to screen 70% of eligible women by 2030 differ.

• “…to evaluate HIV the impact of HIV programs in countries supported by United States President's Emergency Plan for AIDS Relief (PEPFAR)” (line 150)	Thank you for catching this error. We have corrected it on line 150	We deleted HIV (“…to evaluate HIV the impact of HIV programs in countries supported by United States President's Emergency Plan for AIDS Relief (PEPFAR)”
• I am not sure whether “Patient and Public Involvement” is placed in the appropriate section. Is that the journal’s requirement?		Yes, the patient and public involvement statement is required to be at the end of the methods section
• …”primary outcome” …(line 169), “primary”, “secondary” apply to intervention studies as we have two types of variables there but, here, it would have been mentioned simply as “outcome variable”	Thank you for your comments. We have replaced primary outcome with the outcome variable	We deleted primary
• What was the justification for coding Ethiopia’s data set as rural if the population size was smaller than 50,000 people? Would you please mention the source for this categorization?	Thank you for your comments.<br>The categorisation for Ethiopia’s dataset as rural or urban was to standardize the presentation and analysis of the data.<br><br>There is no standard way to define rural or urban.<br><br>The Ethiopia Population-Based HIV Impact Assessment EPHIA 2017 – 2018, Final Report August 2020 defined an large urban area as having a population size greater than 50,000 people and a small areas as having a population size less than 50,000 people.<br><br>In the discussion and presentation of the results we keep the distinction between large and small urban areas.	We add to the text on age 6 as follows:<br><br>“to standardize the presentation and analysis of the data. However, the interpretation of the data regarding Ethiopia is urban variable is linked to the size of the population, not rural or urban category”
• Regarding age, once the WHO created a deliberate inequalities in age (25-49) and have been	Thank you for comments. The rationale for us to examine inequalities in the age-group already targeted by the WHO is that this is the group that is supposed to receive the	We write on page 5: “Examining inequalities in a population not recommended to

providing the pre-cervical cancer screening, what is the need to estimate of the inequalities among the already established age categories? What was unknown about this since it was an established fact, hence WHO took measures on this specific issue?	services, and we wanted to see if women in these countries were receiving the services equitably.<br><br>It would not be correct to examine inequalities in a population that is not recommended to receive the intervention (and may not be receiving the intervention), unless one wants to do a different analysis such as advocating with the WHO to change their guidelines and extend the analysis to a different population group. That is outside the scope of this paper.<br><br>We clarify this point in the manuscript	receive the intervention may be incorrect"
• Do you think logistic regression demonstrates inequality among subgroups of the population with regard to pre-cervical cancer screening services?	Thank you for your question:<br><br>Logistic regression is one step.<br>We performed the post command margins stata command in the marginal effects modelling to determine the predicted probabilities of reporting screening by sub-groups.<br><br>Depending on the sub-groups analysed we calculated the absolute percentage points differences between the groups	We clarify step 3 of the methods section as follows:<br><br>"In step 3, after each logistic regression we used the stata margins post command (marginal effects models) to estimate the probabilities of reporting cervical pre-cancer screening for each predictor-related inequality alone and combined with other predictor-related inequalities, holding the other predictor-related inequalities constant at their mean values"
• "In step 3,...each inequality alone and combined with other inequalities,…" (Lines, 187-188) Where is the combined one? Is it for the multiple logistic regression?	The individual and combined inequalities were the results of the marginal effects model.	See above
How you analysed the differences using multivariable logistics regression after	After each regression we used the Stata margins post commands	See above

determining the associations between screening for cervical pre-cancer and each dimension of inequality?		
What is a common denominator to compare those inequalities than simply performing separate logistic regression for each dataset of each country?	The denominator depends on the predicted values of each sub-group.<br><br>For example, after the logistic regression we used the margins stata command to predict the probabilities of rural, not educated women aged 24 – 34 in lowest wealth quintile to report cervical cancer screening.<br><br>And we do the same prediction for urban, highest educated women aged 35 – 49 in the highest wealth quintiles.<br><br>We then subtract these two figures to find the absolute inequalities in percentage points and compared these with the WHO recommended thresholds for high, medium and lowe inequalities.<br><br>We clarified in the text in line 181	See above and also the following text in line 181<br><br>"We used percentages to measure the magnitude of inequality between subgroups. We denoted a difference of 20% or more between two subgroups as high inequality, less than 20% but greater than 5% as medium inequality and below 5% as low inequality, as used by the WHO"
• How you did the sharpened Qs and why?	We used the sharpened qs to correct for multiple hypothesis testing because it is easier to use and is more powerful than other methods.<br><br>Please see David McKenzie' post<br><br>An overview of multiple hypothesis testing commands in Stata (worldbank.org)	We add to the text that<br><br>"…because it is easier to use and more robust than other method."

Reviewer: 2

Mr. Samuel Yohannes Ayanto, Jimma University, Hosanna College of Health Sciences<br>Comments to the Author:<br>Please leave your comments for the authors below:<br>The research work	Thank you for carefully reviewing our manuscript and your thoughtful questions which have helped to substantially improve the manuscript.	We have made several changes in the manuscript based on your inputs which has substantially improved the manuscript.

addresses a very important public health problem and guides policy makers with its important findings. There are only few comments that would help the authors to revise their work before the manuscript can be considered for publication.		
1. In the main outcomes section of the abstract you indicated the two potential outcomes of your study. In addition you have defined the way how you used the outcome variables which should actually be described under “operational definition” section or under “variables and outcome descriptions” section in your case where you actually described it. Therefore, it is better to remove it from the outcome section of the abstract.		We are not clear what you mean with your querry.
2. In the conclusion under the abstract it says “None of the countries achieved one-third of the World Health Organisation target of screening 70% of eligible women for cervical pre-cancer by 2030.” When is that achievement? Is it during your study period? Specify!	Thank you for your comments. Yes, it is at the time of this study.	We write:<br><br>“As of November 2020, the time of this study”
3. Unless the journal’s requirements for manuscript preparation dictate otherwise, I would prefer the limitation section to be rewritten in the paragraph style than bullet format.	Thank you for your comments. The journal prefers that the limitation section comes in bullet points immediately after the abstract.	We moved the limitation section to below the abstract and changed it into bullet points as suggested by the journal

Also you indicated recall bias as a limitation of the study as it was actually happened. Of course, it might have happened and hampered your findings negatively but still you cannot be 100% sure of it.<br><br>Therefore, you can put the possibility of the introduction of recall bias in to your study, but you cannot be sure of it as if it was actually there.	We agree with you that we are not 100% sure that bias was present. We changed the text to reflect this nuance.<br><br>We wrote<br><br>Recall bias may have been present as cervical pre-cancer screening is self-reported.	To limit to only five bullet points we deleted this limitation
4. On page 5 line 150-152, check for the grammatical correctness of the statement “The PHIA surveys collected a range of health and socio-demographic data to evaluate HIV the impact of HIV programs in countries supported by United States President's Emergency Plan for AIDS Relief (PEPFAR).”	Thank you catching this typo. We have changed the text.	We deleted HIV (“…to evaluate HIV the impact of HIV programs in countries supported by United States President's Emergency Plan for…”
5. On page 5 I recognized two methodological approaches. The first one indicates that you used secondary data analysis. The second one demonstrates the collection of primary data from household members. Was your research a mix of primary and secondary data collection and management approaches? I think this needs additional clarification of the methods in that for which variables you used secondary data and for which you used primary data.	Thank you for your insights. This was a secondary data analysis, i.e. we used existing data collected in the PHIA surveys.<br><br>The second section you refer to clarifies how the PHIA surveys collected the data.<br>We clarify these points on line 160 and 161	We write:<br><br>“We used the Household, and Adult data sets of the **PHIA data**. **In the PHIA surveys,** in participating households, a household questionnaire was administered…”

6. On page 5-6 line168-169, you declared that “these dimensions of inequalities are familiar sources of disadvantage or discrimination.” If so, why did you repeat such investigation, despite the establishment of such well-known scientific facts before? It is also what your findings have confirmed. Would you justify this adequately!	Thank you for comments: We did the analysis considering common sources of ineq ualities to investigate the existence and magnitude of the inequalities in cervical pre-cancer screening in out settings.<br><br>Our findings will support policymakers in setting priorities where interventions will be most urgently needed	
7. On page 7 line 208-210 it says “More than half of the respondents resided in rural areas, excluding Ethiopia, where all respondents resided in urban areas, 54% of them in areas with <50,000 residents.” But on page 6 line 174-175 it says “Ethiopia’s data set had an urban variable, which we coded as rural if the population size was smaller than 50,000 people, and urban otherwise.” Also on page 9 line 226-227 it says “More urban residents than rural residents reported screening for cervical pre-cancer in Ethiopia, Tanzania, Zambia, and Zimbabwe before adjusting for covariates (Figure 1).” Are they all in alignment? If so try to simplify the statements!	Thank you for your insights.	We make the statement consistent on line 235 by starting that<br><br>… “In Ethiopia that data was collected only among urban resident.”<br><br>On page 6 line 187 we state that:<br><br>“However, the interpretation of the data regarding Ethiopia’s urban variable is linked to the size of the population, not to rural or urban category”
8. Page 12 line 267-268 says “None of the countries reached a third of 70% WHO cervical pre-cancer screening target, eight years away from the 2030 deadline.” Is the eight years interval the same for all countries since the survey periods you used for each country were different?	Thank you for your inputs. We agree with you that the survey timeliness differ. However, we use as cut off point the time of this study but also note that one limitation is that the starting points are different.	We state in the limitation section below the abstract that:<br><br>“The countries collected the data at different time points; Ethiopia (2017–2018), Malawi (2015–

		2016), Rwanda (2018), Tanzania (2016–2017), Zambia (2016) and Zimbabwe (2015–2016), and their milestones for reaching the target differ”
9. Your discussion is majorly a repetition of the study findings in your results section. You have also demonstrated inadequate efforts to make comparisons of your findings with what is already there in the world of scientific literature. Also, your recommendations following the discussions usually used the obligatory word “should” which can preferably be expressed in the other way round.	Thank you for your comments:<br><br>We have reviewed our findings in response to you changes and made the several changes	We have rewritten the discussion section, drawing on existing literature, comparing out findings with evidence and arguing for relevant policy change. See our inputs on page 16 starting from line 317 to 454.<br><br>Many thanks for your observations which have helped us, shorten and improve the manuscript.
10. There existed a collinearity problem in the analysis. But you did not mention any measure you have taken as a solution in your manuscript about the observed multicollinearity ef fect between wealth and residence.	Thank you for your comments. We clarified that although collinearity was present, it was not problematic.<br><br>We state this in the limitation section	We write:<br><br>• Collinearity was present, especially between the wealth and residence variables which tend to correlate but was not problematic accordi ng to the variance inflation factor (VIF), which measure multicollinearity in the models. It was less than 2 in the surveyed countries indicating that multicollinearity was not severe in our models.
11. You used very long paragraphs in your	Thank you for your observations which has substantially helped improve the	

manuscript which seem uninteresting and difficult to comprehend its contents easily.	manuscript. We have rewritten the manuscript and shortened it to improve its brevity and readability. We hope it is clear now.	
12. Some acronyms like PHIA and the like have not been addressed fully.	We have defined all abbreviations	

Reviewer: 1
Competing interests of Reviewer: I have no competing interests to declare.

Reviewer: 2
Competing interests of Reviewer: No competing interests

VERSION 2 – REVIEW

**REVIEWER**	Ayanto, Samuel Yohannes<br>Jimma University
**REVIEW RETURNED**	25-Nov-2022

**GENERAL COMMENTS**	The comments have been effectively and adequately addressed at this stage of the manuscript. Therefore, it can be considered for publication in my view.

VERSION 2 – AUTHOR RESPONSE

Reviewer: 2: Mr. Samuel Yohannes Ayanto, Jimma University, Hosanna College of Health Sciences
Comments to the Author:

The comments have been effectively and adequately addressed at this stage of the manuscript. Therefore, it can be considered for publication in my view.

Response to the reviewer:
Many thanks for the comprehensive review which has substantially improved the manuscript.
